# Development and Validation of a SYBR Green Real Time PCR Protocol for Detection and Quantification of Nervous Necrosis Virus (NNV) Using Different Standards

**DOI:** 10.3390/ani11041100

**Published:** 2021-04-12

**Authors:** José G. Olveira, Sandra Souto, Isabel Bandín, Carlos P. Dopazo

**Affiliations:** Unidad de Ictiopatología, Instituto de Acuicultura y Departamento de Microbiología, Universidade de Santiago de Compostela, 15782 Santiago de Compostela, Spain; jose.olveira@usc.es (J.G.O.); sandra.souto@usc.es (S.S.); isabel.bandin@usc.es (I.B.)

**Keywords:** diagnosis, RT-qPCR, quantification standards, fish virus

## Abstract

**Simple Summary:**

In the present report, we designed a reverse transcription real-time quantitative polymerase chain reaction (RT-qPCR) for the detection and quantification of the nervous necrosis virus (NNV), a fish virus of great importance in Mediterranean aquaculture. The advantage of this procedure with respect to others is that it ensures the correct diagnosis of any viral type, at extremely low viral loads, thanks to a limit of detection lower than in previous methods, and demonstrates a better diagnostic sensitivity and specificity than PCR and cell culture isolation. Furthermore, this is the first time that an RT-qPCR procedure has been validated for the quantification of NNV, ensuring the reliability of the quantification, regardless of the calibration standard chosen. Therefore, its use would enable the comparison of data between different laboratories.

**Abstract:**

The nervous necrosis virus (NNV) is a threat to fish aquaculture worldwide, especially in Mediterranean countries. Fast and accurate diagnosis is essential to control it, and viral quantification is required to predict the level of risk of new viral detections in field samples. For both, reverse transcription real-time quantitative polymerase chain reaction (RT-qPCR) is used by diagnostic laboratories. In the present study, we developed an RT-qPCR procedure for the diagnosis and simultaneous quantification of NNV isolates from any of the four genotypes. The method proved to be highly sensitive in terms of crude virus titer: 5.56–9.88 TCID_50_/mL (tissue culture infectious dose per mL), depending on the viral strain, and averaging 8.8 TCID_50_/mL or 0.08 TCID_50_/reaction. Other standards also yielded very low detection limits: 16.3 genome copies (cps) of purified virus per mL, 2.36 plasmid cps/mL, 7.86 in vitro synthetized RNA cps/mL, and 3.16 TCID_50_/mL of virus from infected tissues. The diagnostic parameters evaluated in fish samples were much higher in comparison to cell culture isolation and nested PCR. In addition, the high repeatability and reproducibility of the procedure, as well as the high coefficient of determination (R^2^) of all the calibration curves with any type of standard tested, ensure the high reliability of the quantification of NNV using this RT-qPCR procedure, regardless of the viral type detected and from the type of standard chosen.

## 1. Introduction

Viral encephalopathy and retinopathy (VER), caused by nervous necrosis virus (NNV), is one of the most threatening viral diseases affecting marine and, in a lesser extent, freshwater farmed fish worldwide. Furthermore, a high number of wild fish species has been reported to be susceptible to the virus and disease outbreaks have also been recorded mainly in the Mediterranean area [1]. The main target of NNV is the central nervous system, including the brain, spinal cord, and retina. Therefore, clinical signs of the disease include neurological symptoms such as uncoordinated swimming behavior and lethargy, as well as anorexia, particularly during larvae and juvenile stages.

NNV is a member of the family *Nodaviridae*, genus *Betanodavirus*, which includes non-enveloped virions of about 25 nm in diameter with icosahedral symmetry. The viral genome consists of two single-stranded (ssRNA) segments, RNA 1 and RNA2. The first (3.1 Kb) encodes the RNA-dependent RNA polymerase (RdRp), whereas RNA2 (1.4 Kb) encodes the capsid protein [2,3,4]. In addition, a subgenomic RNA3 is synthesized during RNA replication from the 3′ terminus of RNA1 and encodes two non-structural proteins B1 and B2 [5,6]. Based on the comparison of an RNA2 variable region, the betanodaviruses are classified into four genotypes: striped jack nervous necrosis virus (SJNNV), tiger puffer nervous necrosis virus (TPNNV), red-spotted grouper nervous necrosis virus (RGNNV), and barfin flounder nervous necrosis virus (BFNNV) [7]. Moreover, in recent years reassortants between RGNNV and SJNNV genotypes (in both forms RGNNV/SJNNV and SJNNV/RGNNV (RNA1/RNA2)) have been isolated from farmed and wild fish in Southern Europe [8,9,10,11].

Several diagnostic methods have been developed since the disease was first recorded, including histopathology, serological/immunological, and molecular methods such as PCR-based and other genome amplification tools [1]. However, at present cell culture isolation followed by immunostaining or molecular identification is still considered to be the gold standard for diagnostic purposes by the World Organization for Animal Health (OIE [12]). Despite this, PCR-based techniques have been gaining ground in NNV detection because of their speed, specificity, and sensitivity, and in recent years real-time RT-PCR or RT-qPCR protocols are the most commonly used, most of them targeted to RNA2 and based on TaqMan^®^ probes [13,14,15,16,17]. In most of them the method was confirmed to detect strains from the four viral types, however, quantification was evaluated against only one of them. Therefore, since the reliability of viral quantification must be ensured for any detected strains, we thought it necessary to develop a method to ensure the detection and reliable quantification of isolates from any of the four viral nervous necrosis virus (VNNV) types. Although RNA2 was the most frequent target, we carried out several preliminary attempts, testing primer sets for both segments, but only one of them—located in RNA1—achieved promising results.

In this report we present an SYBR Green RT-qPCR protocol targeted to RNA1 capable of detecting all four NNV genotypes, which was evaluated in terms of analytical sensitivity and specificity, repeatability, and reproducibility, not only for detection, but also for quantification. The evaluation was accomplished using crude and purified virus, a DNA plasmid, in vitro transcribed RNA, and infected fish tissues. The analytical properties of the protocol indicated that it shows a high sensitivity and can be useful for detection and assessment of NNV load in both cell culture and fish samples. In addition, its evaluation in infected fish tissues from both experimentally and naturally infected fish validates the procedure to be used as a reliable method of diagnosis and simultaneous quantification of the virus.

## 2. Materials and Methods

### 2.1. Cells, Virus, and Viral Titration

Reference strains belonging to the 4 NNV genotypes were used in this study: SJNag93 (SJNNV), SGWak97 (RGNNV), JFIWa 98 (BFNNV), and TPKag 93 (TPNNV) (Table 1). The viruses were propagated in semiconfluent E-11 cells (ECACC #01110916) cultured at 25 °C in Leibovitz culture medium (L-15; Lonza, Porriño, Spain), supplemented with 5% fetal bovine serum (FBS; Lonza). Inoculated cells were incubated at 20 °C (BFNNV and TPNNV) or 25 °C (RGNNV and SJNNV) for up to 10 days. Culture fluids were recovered when the cytopathic effect (CPE) became extensive, and the cell debris was removed by centrifugation at 3000× *g* for 15 min at 4 °C. Aliquots of the viral suspension were frozen (−80 °C) and stored until use. The viral titrations were performed in 96-well plates using the end-point dilution method, applying 3 replicas per dilution, from dilutions 10^−1^ to 10^−9^, and the titers expressed as the tissue culture 50% infectious dose (TCID_50_) calculated as described by Reed and Müench [18].

### 2.2. Virus Purification

The viruses were cultured in 10 flasks of 150 cm^2^ with semiconfluent E-11 cells, and crude virus was collected when the monolayers displayed an extensive CPE. The viral suspensions were subjected to centrifugation at 4000× *g* for 20 min and the pellet was resuspended in 250 µL SSC 1X buffer (0.15 M NaCl, 0.015 M sodium citrate, pH 7.0), sonicated for 30 s (20 kHz, in 3 steps of 10 s), and subjected to 4000× *g* for 20 min. The virus was recovered by ultracentrifugation at 100,000× *g* for 90 min at 4 °C, loaded onto a layer of 30% sucrose (wt/vol, in SSC 1X buffer), and pelleted again at 120,000× *g* for 1 h at 4 °C. The virus pellet was resuspended in 200 µL of SSC 1X buffer and subjected to fractionating by equilibrium sucrose density-gradient centrifugation, using discontinuous gradients prepared by layering 2 mL volumes of 60, 50, 40, 30, and 20% (wt/vol) sucrose in SSC 1X buffer into 17 mL polyallomer tubes (Beckman). After ultracentrifugation at 100,000× *g* for 1 h at 4 °C, the visualized band was removed and washed by centrifugation at 100,000× *g* 90 min in 30% sucrose. The final pellet was resuspended in 200 µL of SSC 1X and stored at −20 °C until use.

### 2.3. RNA Extraction

Total RNA was extracted directly from viral suspensions using the RNeasy Mini kit (Qiagen, Madrid, Spain) according to the manufacturer’s instructions. RNA samples were resuspended in nuclease-free water (Promega, Madrid, Spain), quantified by absorbance measurement at 260 nm using a NanoDrop ND 1000 Spectrophotometer (NanoDrop Technologies, Wilmington, NC, USA), and stored at −20 °C until use. The quality of the extracted RNA was evaluated from the ratio A260/A280 [20].

### 2.4. Primers Design

An in silico test, using the software AlleleID v7.74 (PREMIER Biosoft, San Francisco, CA, USA), was performed to select primers for the detection of strains from the four NNV genotypes. Multiple sequence alignment using Clustal X (2.0) included both genomic sequences of NNV strains representative of the 4 genotypes (SGWak97, Acc no. NC_008040 and NC_008041; JFIwa98, Acc no. NC_013458 and NC_013459; TPKag93, Acc no. NC_013460 and NC_013461; SJNag93, Acc no. AB056571 and AB056572) (Table 1). Fifteen primer sets putatively amplifying amplicons of 78–178 bp were pre-selected (Supplementary Table S1) and tested by PCR using SYBR Green I detection chemistry, as described below, against the 4 NNV type strains, as well as an RGNNV/SJNNV reassortant strain (SpSs-IAusc160.03; [9]). Additionally, the sets providing the best results with the four strains were tested against a series of field samples belonging to our isolates library (Supplementary Table S2), and some obtained from other researchers. Finally, the following primer set—which provided the best results in the previous assays—was selected for thorough testing in the present study: NodR1_1619 (5′-TCCAAAAGAAAGAAGCATAC-3′) and NodR1_1758 (5′-TGGCATGTACCACGGAAC-3′) between nucleotide positions 1619 and 1758 (139 bp) of strain SGwak97 RNA 1.

### 2.5. cDNA Synthesis

The synthesis of cDNA from the extracted viral RNA was performed in a MyCycler thermal cycler (Bio-Rad, Madrid, Spain). An initial mixture of 2.5 ng/µL random primers (Promega, Madrid, Spain) and 9 µL (approximately 0.15–2 ng/µL, depending on the standard) of viral RNA in nuclease-free water (Bio-Rad, Madrid, Spain), was heated at 95 °C for 5 min and cooled down to 4 °C for at least 1 min. A reverse transcription mixture containing Superscript™ III reverse transcriptase (10 U/mL; Invitrogen, Madrid, Spain), RNaseOUT™ Recombinant RNase inhibitor (2 U/mL; Invitrogen), dNTPs (0.5 mM each), and dithiothreitol (0.05 M DTT) in First Strand buffer (Invitrogen) was added and the final solution (20 µL) was incubated at 25 °C for 10 min, followed by 50 min at 50 °C. Then the enzyme was inactivated by heating at 85 °C for 5 min.

### 2.6. SYBR Green qPCR

The PCR reactions were carried out in a final volume of 20 μL, containing 100 nM of each primer and 2 μL of cDNA (synthetized in the previous step) in iQ™ SYBR^®^ Green Supermix (Bio-Rad, Madrid, Spain). Following an initial 3 min denaturation/activation step at 95 °C, the mixture was subjected to 45 cycles of amplification (denaturation for 15 s at 95 °C; annealing and extension for 15 s at 58 °C) in a CFX Connect™ Thermal Cycler (Bio-Rad, Madrid, Spain). The generation of PCR products was monitored after each extension step at 58 °C by measuring the fluorescence of double-stranded DNA binding SYBR Green dye. In order to determine the melting temperatures of the amplified products after SYBR Green qPCR, the temperature was raised from 55 °C to 95 °C and the fluorescence was detected for 10 s after each 0.2 °C. From each reaction, the threshold cycle value (Ct) was established as the cycle number at which fluorescence was detectable over the threshold value calculated by the iCycler iQ™ software (Bio-Rad, Madrid, Spain) for cycles 2–10.

### 2.7. Analytical Sensitivity and Specificity

To assess the sensitivity of detection of the designed RT-qPCR diagnostic and quantification procedure, the limit of detection (LOD) was evaluated against several standards used as reference (prepared as described in Section 2.9): viral titer of crude virus, RNA copies from crude and purified virus, and copies of plasmid DNA (pDNA) and in vitro transcribed RNA (*iv*RNA). To this end, serial dilutions of each standard were prepared, RT-qPCR was applied to each dilution (in triplicate), and the LOD was deduced from the lowest dilution providing effective amplification with the three replicas (“The last dilution showing 100% response may be accepted as a conservative estimate of the lower limit of detection,” as stated by the OIE for the validation of diagnostic assays for infectious diseases [12]). To assess the specificity, the 4 NNV strains described before were tested (intra-group specificity). In addition, the procedure was tested against infectious pancreatic necrosis virus (IPNV), Spajarup (Sp) strain, and viral hemorrhagic septicemia virus (VHSV), VHSV_Ssp11 strain (genogroup III) (inter-group specificity).

### 2.8. Repeatability and Reproducibility (R&R)

Repeatability is known as the precision of a measurement simultaneously applied on a minimum of 3 replicas of a sample, by the same operator using the same protocol, materials, and equipment. Reproducibility is the precision changing any or several of those parameters: day, operator, procedure, materials, and/or equipment. Repeatability and reproducibility were evaluated as the intra-assay and inter-assay (respectively) coefficient of variation (CV = standard deviation/average Ct × 100). The CV was calculated from the Ct values of the 3 replicas (intra-assay), or from the average values (from the 3 replicas) in three runs performed on non-consecutive days (inter-assay). Values of CV ≤ 10% were considered high repeatability and reproducibility, and CV ≤ 5% were indicative of very high R&R.

### 2.9. Reference Standards for Quantification

As indicated above, five types of templates were tested as standards of quantification:

1- Viral titers and RNA copies from crude virus.-The 4 reference strains were propagated in E-11 monolayers and, when CPE was extensive, the cell debris was removed by low speed centrifugation (2000× *g*, 10 °C, 15 min). Then the clarified virus in the supernatant was subjected to viral titration and RNA extraction as previously described. The number of copies corresponding to the quantity of RNA extracted was calculated from the formula γ = n/N × GL × NcMw, where γ is the amount (in grams) of viral RNA, n is the number of genomic RNA molecules (genome copies), N is the Avogadro number (6.022 × 10^23^), GL is the genome length in nucleotides, and NcMw is the average molecular weight of a nucleotide (350.5 Da). From Okinaka and Nakai [21], the GL values (length in nucleotides of RNA1/RNA2) for the different NNV genotypes are 3107/1421, 3105/1434, 3101/1433, 3112/1422, corresponding to SJNNV, RGNNV, BFNNV, and TPNNV, respectively. With these data, the average weight of a NNV genome is 2.410 ag for the SJNNV type, 2.416 ag for RGNNV, and 2.413 ag for BFNNV and TPNNV.
2- Purified virus. After purification of crude virus from 10 flasks of 150 cm^2^ as described above, the RNA was extracted and quantified, and 20-fold dilutions (from 5.12 × 10^9^ to 4.07 × 10^0^) were subjected to RT-qPCR as described.
3- Plasmid DNA. pDNA was constructed with a 3067 pb nucleotide fragment from genomic RNA 1 of the SGWak97 strain (RGNNV). AccuPrime™ Taq DNA Polymerase High Fidelity (Life technologies, Thermo Fisher, Madrid, Spain) was used according to the manufacturer’s specifications, employing specific primers (VNNR1_1F, 3′-CGCAAGGTTACCGTTTAGC-5′, and VNNR1_5L, 3′-GCCGAAGCGTAGGACAGCATAAAG-5′) designed to amplify that fragment. The PCR products were purified using the QIAquick gel extraction kit (Qiagen, Valencia, CA, USA) and ligated into the pGEM^®^-T vector using the pGEM^®^-T Vector System I (Promega, Madrid, Spain). Plasmids were transformed into DH5α competent cells (Life Technologies, Thermo Fisher, Madrid, Spain) and cultured under appropriate antibiotic selection, as indicated by the manufacturer. The cloned DNA plasmids were extracted and purified using the GeneJET™ Plasmid Miniprep Kit (Fermentas, Thermo Fisher, Madrid, Spain). Plasmid copies were estimated based on spectrophotometric analysis. The mass of a single pDNA molecule was calculated using the same formula for γ described above, where GL = 3067 nc, and NcMw (the average molecular weight of each pair of nucleotides) was estimated as 660 g/mol. The concentration of the resulting plasmid solution was around 50 ng/μL (corresponding to 7.50 × 10^9^ copies/μL); 20-fold serial dilutions were prepared and subjected to qPCR amplification as described.
4- In vitro transcribed RNA. Approximately 400 ng of the previously constructed plasmid were linearized by digestion with Sal I in a 20 µL reaction according to the enzyme manufacturer’s instructions (FastDigest^®^SalI, Fermentas, Thermo Fisher, Madrid, Spain). The reaction mix was incubated 15 min at 37 °C, followed by a heat inactivation at 65 °C for 10 min. The linearized DNA was purified using the QIAquick PCR purification kit (Qiagen, Valencia, CA, USA) following the manufacturer’s instructions. RNA transcripts were generated using the MEGAscript^®^ Kit (Ambion, Thermo Fisher, Madrid, Spain). Briefly, approximately 200 ng of linearized plasmid were added to 20 µL of transcription reaction containing 5 mM of each ribonucleotide and 2 µL of enzyme mix in 10X reaction buffer and incubated at 37 °C for 4 h. A DNase treatment was conducted using Ambion’s TURBO™ DNase Kit (Ambion, Thermo Fisher, Madrid, Spain) for 30 min at 37 °C. The RNA was recovered using the MEGAclear™ Kit (Ambion, Thermo Fisher, Madrid, Spain) following the manufacturer’s instructions. RNA concentration and copy number were estimated as above. From the formula, 1 ng of RNA 1 *iv*RNA was estimated to contain 5.76 × 10^8^ copies. Twenty-fold serial dilutions were prepared and subjected to RT-qPCR as above.

### 2.10. Reliability of the Viral Quantification in Infected Fish Tissues

A volume of 1 mL of titrated crude virus (SGWak97 strain) was mixed with 1 g of non-infected gilthead seabream brain (the fish were obtained from a fish market and checked by RT-qPCR before use). The mixture was homogenized using a Tissue Master 125 homogenizer (OMNI International, Kennesaw, USA) and incubated at 25 °C for 24 h. Afterwards, the tissue was pelleted at 3000× *g* for 20 min and both phases, supernatant and pelleted tissue, were titrated separately as described above. In parallel, non-infected seabream brain was similarly processed (suspended in one volume of L-15 medium) to prepare the serial *w/w* dilutions of the infected pelleted tissue, starting with a mixture of 400 µL of infected pellet + 400 µL of non-infected tissue, and continuing with dilutions of 1/5, 1/10, 1/100, and 1/50 using non-infected tissues). From each dilution, 200 µL were subjected to RNA extraction and RT-qPCR amplification as described above.

### 2.11. Assessment of the RT-qPCR on Experimentally and Naturally Infected Fish

1- Fish Challenge. Senegalese sole (*Solea senegalensis*) (35 fish averaging 1 g) and sea bass (*Dicentrarchus labrax*) (45 fish averaging 7–8 g) were obtained from commercial fish farms. Prior to experimental infection, fish were acclimatized for 10 days at the aquarium facilities of the University of Santiago de Compostela at a low density (1 fish/L) and the experimental temperature (23 °C for Senegalese sole and 25 °C for sea bass). During this period, 5 individuals from each species were sacrificed with an anesthetic overdose (MS-222, Sigma-Aldrich, St. Louis, MO, USA) and tested for the presence of NNV.

The fish were handled according to the guidelines for good practices in laboratory animals of the European Union (directive 2010/63/EU). For the challenge, protocols approved by the Galician committee for experimental animal welfare (Xunta de Galicia, Permit ID 15010/2020/004) were followed to minimize fish suffering.

Sole (N = 22) and sea bass (N = 29) individuals were infected by immersion with a viral concentration of 10^5^ TCID_50_/_mL_ of RG/SJ (SpSs-IAusc160.03 strain) or RG/RG (SGWak97 strain) strains (respectively) (Supplementary Table S2), as previously described [22]. A control group corresponding to each species (N = 5) was handled like the infected groups and mock infected with L-15 medium. The dead fish were immediately removed and frozen at −20 °C until subjected to viral detection by the RT-qPCR procedure described here, with cell culture isolation and nested PCR (nPCR) [22]. After 3 weeks, surviving fish were killed by an overdose of MS-222 and stored at −20 °C until viral detection.

2- Evaluation of the diagnostic reliability. For the evaluation of the diagnostic reliability, the following parameters were assessed [23]: (i) Diagnostic sensitivity (DSs): the frequency of infected fish detected as positive by the diagnostic procedure; (ii) diagnostic specificity (DSp): the frequency of non-infected fish correctly diagnosed as negative; (iii) predictive positive value (PPV): frequency of true positives yielded by the procedure; (iv) predictive negative value (PNV): frequency of true negatives yielded by the procedure.
3- Assessment of the RT-qPCR on field samples. Additionally, the RT-qPCR procedure was evaluated in field samples from farmed and wild fish.
  a- Assessment on samples from fish farms. A total of 406 fish from 3 species (9 turbot, *Scophthalmus maximus*; 99 gilt-head seabream, *Sparus aurata*; 298 Senegalese sole, *Solea senegalensis*) received from several European fish farms (mainly from the Iberian Peninsula) in 2019 and 2020 were subjected to viral detection applied with the three procedures cited above. Three types of tissues and organs were employed, depending on the age of the fish: blood, from breeders, or brain and/or a pool of kidney, spleen, and heart from fish of other ages.
  b- Surveillance of fish from coastal populations. In an epidemiological campaign during 2019 and 2020, a total of 433 fish from 24 commercial species were bought in fresh fish markets in different coastal location of Galicia (NW Spain), and pools of heart, spleen, kidney, and brain were subjected to viral detection using the 3 methods indicated above.

## 3. Results

### 3.1. Sensitivity and Specificity

Regarding the sensitivity (Table 2), the limit of detection of RNA from crude virus averaged 0.46 fg/µL of extracted RNA, corresponding to around 1.91 × 10^5^ copies/mL. However, we must point out that although the lowest concentrations (26.5, 25.0, 23.5, and 23.0 ag/µL with SJ, RG, BF, and TPNNV types, respectively) provided no amplification (results not shown), due to the 20-fold serial dilutions applied (higher than the usual 10-fold dilutions), a LOD slightly lower than the observed cannot be ruled out. In terms of viral titer, the LOD of crude virus averaged 8.8 TCID_50_/mL, which is close to 10^4^ times lower than the LOD as RNA copies per mL. Furthermore, using purified virus, the LOD value was 1.63 × 10^1^, which is close to the LOD using crude virus. The LOD values with pDNA (2.36 copies/mL) and *iv*RNA (7.86 copies/mL), as well as with infected fish tissues (3.16 TCID_50_/mL), were also closer to the value obtained with viral titer than with RNA copies from crude virus.

### 3.2. Validation of the Procedure for Quantification

Quantification using crude virus as standard. Two types of standard curves were obtained using crude virus: curves employing the viral concentration measured by a traditional titration procedure (TCID_50_/_mL_ in this study) as standard, and those employing the number of RNA copies calculated from the RNA concentration of the same crude virus sample.

The data (as Ct values) obtained in all the assays (three repeats, three replicas each) are shown in Supplementary Table S3. To ensure that the viral concentrations tested were the same for the four viral types, the initial viral titers were adjusted to 3.16 × 10^7^ TCID_50_/mL for three of the types (SJNNV, RGNNV, and TPNNV), and were slightly lower (1.78 × 10^7^ TCID_50_/mL) with BFNNV. Those titers corresponded to initial RNA concentrations of 1.7, 1.6, 1.5, and 1.1 ng/µL (SJNNV, RGNNV, BFNNV, and TPNNV, respectively), and matched to 7.05, 6.62, 6.22, and 4.56 × 10^11^ copies/mL (respectively) by the formula described above. These data reveal a difference of around 4 log_10_ between both ways of measuring the concentration.

Table S3 shows the results only for dilutions providing amplification (Ct < 40) with the three replicas and in all the repeats. Thus, the dynamic range (DR) with this procedure was 6 Log_10_ (Table 2). The coefficient of variation was in most cases below 5% (always below 10%) in all replicas, which denotes a very high repeatability. Analyzing the repeats, the CV values were again below 5%, also demonstrating a very high reproducibility using the same equipment. In addition, using the data from the four types, the CV values still were below 5% (Supplementary Table S3E), which demonstrates the high repeatability and reproducibility of the procedure, regardless of the viral type.

**Figure 1:**
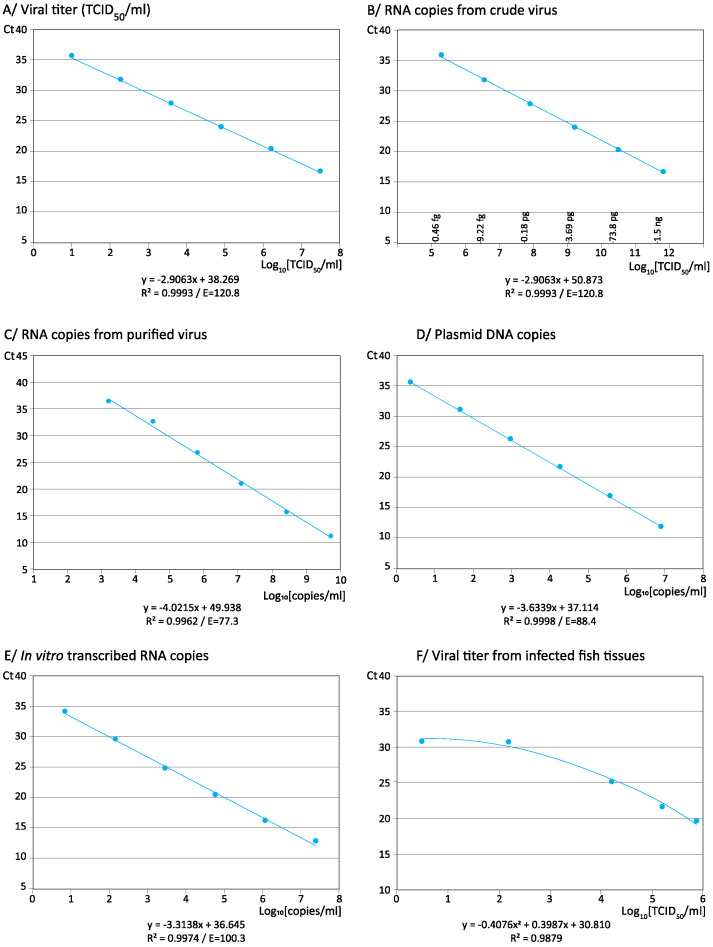
Calibration curves using different standards: averaging data from the four strain types. All the data from replicas and repeats were averaged to obtain a general calibration curve for each standard: viral titer (**A**), RNA copies from crude virus (**B**) or from purified virus (**C**), pDNA copies (**D**), *iv*RNA (**E**), or for viral quantification from infected tissues (**F**).

To simplify the interpretation of the results, the equations, reliability, and efficiency of viral titer and RNA copy standard curves are shown in Table 3 (all data and the corresponding curves are in Supplementary Table S3 and Supplementary Figures S1 and S2). Regardless of the strain, all the curves were reliable: R^2^ > 0.95 in all replicas and repeats, and in most cases were higher than 0.99. In addition, the procedure showed high reliability across all four viral genotypes (average standard curve R^2^ = 0.9993; Figure 1A,B). As expected, the differences between line equations using viral titer and RNA copies were just in the intersection value; this is because the slopes must necessarily be the same given that both share the same Ct values.

On the other hand, the efficiency of the RT-qPCR amplification, deduced from the slopes, were in all cases over 100%, with values closer to 100 in the case of SJNNV and RGNNV strains (Table 3).

Quantification using purified virus as standard.-Only purified virus from SJNNV and RGNNV strains was used (Supplementary Table S4). From the volume of purified virus subjected to RNA extraction (100 µL), concentrations of viral RNA of 56.9 and 34 ng/µL (RGNNV and SJNNV, respectively) were obtained. RNA samples from both viruses were adjusted to initial concentrations of around 13.75 ng/mL, which approximately corresponded to 5.21 × 10^9^ copies/mL. Serial 20-fold dilutions were applied until a final concentration of 8.15 × 10^1^ copies/mL was achieved. As observed, with the SJNNV type no positive amplification was obtained with the lowest RNA concentration (Ct ≥ 40, or—in most cases—no amplification at all), which gave a DR of 6 Log_10_. However, with the RGNNV strain the DR was 7, since positive amplification was attained at the lowest RNA concentration (Ct around 38 in the three replicas and repeats). Repeatability and reproducibility (R&R) were also very high since CV values below 5% were obtained in all cases (average CV = 1.7%; result not shown).

The reliability of each standard curve was always high (R^2^ > 0.99) with any viral type (Table 4 and Supplementary Figure S3). The general standard curve with purified virus (averaging data from both strains) was also demonstrated to be reliable (R^2^ = 0.9962; Figure 1C). Unlike those observed with crude virus, the RNA amplification efficiencies were below 100% in all cases, obtaining average E > 84% with SJNNV, and lower values (under 80%) with RGNNV.

Quantification using plasmid DNA as standard. A plasmid DNA carrying a 3067 bp cDNA fragment corresponding to the RGNNV strain was constructed and used as standard for NNV quantification. Amplification was reached at concentrations from 7.55 × 10^6^ to 2.36 × 10^0^ copies/mL, which meant a DR of 6 Log_10_ (Supplementary Table S5); no amplification was obtained at the lowest concentration tested (1.18 × 10^−1^ copies/mL). Again, R&R was revealed to be very high (CV values ranging from 0.18 to 3.28). Regression curves were demonstrated to be highly reliable: R^2^ > 0.999 in the three repeats (Table 5; Supplementary Figure S4), and close to 1 in the general standard curve (Figure 1D). As with purified virus, the efficiency of the amplification was below 100 (E = 88.4 for the general curve).

Quantification using in vitro transcribed RNA as standard. In vitro transcription was applied on the previously described plasmid to obtain a transcribed RNA corresponding to the RNA 1 of the RGNNV type strain. As with the plasmid, only six concentrations (from 2.52 × 10^7^ to 7.86 × 10^0^ copies/mL) rendered positive amplification (DR = 6) (Supplementary Table S6 and Table 6). All the CV values were below 5% (ranging from 0.13 to 3.93), denoting high R&R. The high reliability of this standard was demonstrated by the high R^2^ (>0.99) of all the regression lines (Table 6; Supplementary Figure S5, including the general curve for *iv*RNA (R^2^ = 0.9974) (Figure 1E). Regarding the amplification efficiency, this standard was the only one providing E values close to 100: ranging from 96.1 to 104.7 considering all replicas, and from 99.6 to 101.4 considering the repeats, and obtaining a general standard curve with E = 100.3.

Quantification using virus from infected fish tissues as standard. Fish tissue infected in vitro with a known concentration of virus was diluted with non-infected tissue and subjected to RNA extraction and RT-qPCR as described. In a first assay, three sets of extraction were applied to the same tissue sample, and three replicas in three repeats of RT-qPCR amplification were performed within each one. It is worth mentioning the demonstrated repeatability and reproducibility of the assays, since in most cases the CV values were below 5% (and always below 10%) (Supplementary Table S7). More importantly, CV values were also below 10 even considering the Ct values from all the extractions, denoting the high R&R of this type of standard.

To analyze the reliability of such a standard, three types of curves were drawn. As shown in Supplementary Figure S6 and summarized in Table 7, the linear regression applied to the data yielded curves of low reliability (R^2^ < 0.95) and extremely high E values (between 160 and 230). Suspecting that the failure could be due to the highest dilution, the regression analysis was repeated, eliminating the lowest viral titer, which made it possible to obtain highly reliable curves (R^2^ < 0.99) with lower E values (around 115%). This result pointed towards a low efficiency of the procedure at the lowest viral concentrations in fish tissues. Therefore, a second degree polynomial regression analysis was applied, yielding reliable curves in all cases (Supplementary Figure S6), including the general curve (Figure 1F).

In a second assay, a wider range of viral concentrations was tested (from 3.16 × 10^0^ to 2.51 × 10^7^ TCID_50_/gr), and the same RT-qPCR procedure was applied as described, but without replicas or repetitions (Supplementary Table S7-Assay II). Similar to Assay I, the linear regression using all the concentrations was not reliable (R^2^ = 9372), but adjusted to a second degree polynomial line (R^2^ = 0.9975). The regression removing the lowest concentration (calculated between concentrations 6.31 × 10^1^ and 2.51 × 10^7^) was reliable (R^2^ = 0.9611), and was not significantly different from that obtained in Assay I (results not shown).

### 3.3. Comparison Among All the Standards

**Figure 2:**
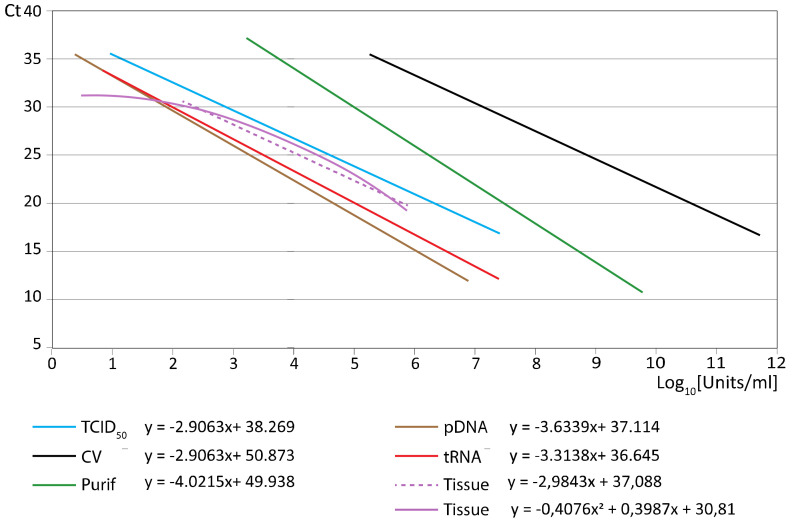
Comparison of standard curves. The regression lines with each type of standard averaging the data from the 4 strain types assessed are compared.

The general curves (averaging data from all replicas, repeats, and strains tested) for each type of standard are compared in Figure 2. As observed, depending on the type of standard employed, a same Ct may correspond to different concentrations of viral units (TCID_50_ or copies). The curves with purified virus and with crude virus RNA copies (green and black lines, respectively) are those that provided the highest concentration values: around 3–4 and 5–6 Log_10_ (respectively), at the highest Ct values, and close to 10 and 12 at the lowest, respectively. Standards pDNA and *iv*RNA provided the lowest quantification values: less than 1 Log_10_ at the highest Ct, and around 7 at the lowest. In between both groups, although closer to pDNA and ivRNA standards, was the quantification curve using viral titer standard as reference (blue line). The quantification curve corresponding to fish samples was also close to that of viral titer. It is important to remark that when drawing each standard curve averaged from all replicas and repeats, the reliable maximum Ct was reduced from around 40 to 36–37.

### 3.4. Assessment of the Procedure for VNNV Detection and Quantification in Field Samples

1- Validation of the procedure in challenged fish. Sea bass and sole fish were infected with RG/RG and RG/SJNNV strains as described. One week after the challenge, the individuals of both fish species began to show anorexia and abnormal swimming behavior. Skin darkening was more frequent among sea bass than sole. No clinical signs or mortality were observed among the mock fish. The virus was detected by RT-qPCR in all infected individuals of both species, at Ct values ranging from 18.22 to 35.11 with sole samples, and from 4.35 to 34.33 with sea bass tissues (Table 8). Among the 22 positive sole fish, nPCR yielded a positive result in 19 (corresponding to RT-qPCR Ct values of 18.22–32.83), and the virus was isolated only from 13 of the infected sole. Regarding the sea bass, the virus was isolated from 25 out from the 29 infected individuals by isolation in cell culture, and the nPCR failed in two fish, those corresponding to Ct > 32. The corresponding diagnostic parameters are also shown in Table 8. Since all inoculated fish showed some clinical sign, we considered as the gold standard that all the inoculated fish should have been really infected. Regardless of the fish species, the only diagnostic procedure providing the maximum DSs, DSp, PPV, and PNV values was the RT-qPCR validated in this study. The diagnostic specificity and the negative predictive values were also maximum with isolation in cell culture, nPCR, or considering both in a parallel test. However, both the diagnostic sensitivity and negative predictive values were lower than with RT-qPCR.

In Table 8, the observed viral titers, calculated using the standard curve for crude virus titer, are shown to be higher in sea bass than sole. That type of standard curve was employed after demonstrating that no significant differences existed between the titer data extracted from the linear standard curves by crude virus and fish tissue viral titer, as well as with the fish tissue second degree polynomial curve in the Ct range of 11–5 (Supplementary Table S8).

2- Evaluation of the procedure in farmed and wild fish

During 2019 and 2020, the RT-qPCR procedure was evaluated in fish samples from turbot, gilt-head seabream, and Senegalese sole farms, against the traditional cell culture isolation and the nPCR procedure previously used in our laboratory [22]. Among the more than 400 samples, 37 resulted positive by RT-qPCR, with Ct values between 25.02 and 37; among those, only 10 were positive with nPCR, those with Ct values by RT-qPCR below 33, and only in four of those samples could the virus be isolated (Table 9).

In the same period, 433 fish from 24 species, obtained from coastal fish markets, were sampled and subjected to viral detection by the same three methods. The virus was not isolated in any case, and was detected in just 11 cases by nPCR (those yielding Ct 28.75–32.7 by RT-qPCR) and in 48 fish by RT-qPCR (with Ct values from 28.75 to 33.2) (data not shown). Positive fish were mainly from three species: European hake (*Merlucius merlucius*), sea bass (*D. labrax*), and megrim (*Lepidorhombus boscii*). These results are part of a further report on the presence of VNNV in the Northwest Spain.

## 4. Discussion

Molecular detection of viruses is of unquestionable importance in virology. This fact is demonstrated by the large number of publications on the design, optimization and validation of PCR-derived procedures, which in the last few years has been most focused on real-time PCR. This technique, in addition, has made it possible to simplify viral quantification, which is currently gaining relevance as a basic complement in research. Concerning fish nodaviruses, RT-PCR and real-time PCR procedures have been widely reported for detection and identification of the virus [1], but very few publications deal with the design and validation of RT-qPCR for quantification of this virus. Among the latest, three were designed with a similar strategy to that reported here, namely, absolute quantification, using external standards for constructing calibration curves [14,15,17]. A fourth one used a less common approach: competitive real-time RT-PCR [24]. Most of them focused the validation assays on the RGNNV type, probably because it is the most widely spread genotype [1], although some used different genotypes and a large number of strains for susceptibility analysis [15,17].

The detection and quantification procedure described here was initially thought to be tested against RGNNV, the most frequently isolated in the Iberian Peninsula [1]. However, the primers were designed to fit four NNV strains representing the four genotypes. The 14 primer pairs pre-selected in silico for sequences in RNA-1 and RNA-2 were tested in vitro with the four strain types. Many of them failed in repeatability and reproducibility and/or in specificity; among these, some were even more appropriate for typing (and will be part of a future report). Two sets, those providing the best results in specificity and R&R (NodR1 and NodR2), were also tested against field isolates. Among them, the one designed for RNA 2 was rejected for not providing the expected repeatability against some recombinant isolates (data not shown). Therefore, the NodR1_1619/NodR1_1758 set was selected to optimize and validate the procedure for detection and quantification. This first result does not agree with the strategy followed by Panzarin et al. [15], who used a primer set corresponding to RNA 2, or by Hick and Whittington [14], who obtained better results with sets also located in that segment. However, in a more recent report [17], Baud et al. opted for RNA 1, expecting to obtain better results with any of the viral genotypes. This was also our objective. Therefore, although in some specific assays of our study (using pDNA, *iv*RNA, and fish tissues) the reliability of the procedure was assessed only with one viral type (RGNNV), SJNNV and RGNNV were tested with purified virus as standard, and the four genotypes with crude virus.

Crude virus was precisely the first type of standard tested as a reference of quantification in this study, and the results demonstrated that procedure was highly sensitive. In fact, the limit of detection, in terms of viral titer, averaged 8.8 TCID_50_/mL (average from the four strain types). However, to compare with similar reported studies, we needed to use the result obtained with the RGNNV type (9.88 TCID_50_/_mL_), which was similar to the 10 units reported by Panzarin et al. [15]. In addition, the level of sensitivity of our procedure exceeded by 30 or 60 times the one reported by Baud et al. [17] (LOD: 10^2.5^–10^2.8^ TCID_50_/mL). On the other hand, the data reported by Hick and Whittington [14] seemed to be much lower (LOD = 0.4 TCID_50_) than those obtained with our procedure. Nevertheless, it must be noted that the value reported by those authors was absolute, corresponding to units per reaction, and it was four times higher than the LOD per reaction showed by our procedure (LOD = 8.99 × 10^−2^ TCID_50_).

In terms of RNA concentration, the limit of detection averaged 0.46 fg/µL, which was much lower than that reported by Toubanaki et al. [25] for the RGNNV (10 fg/µL) and SJNNV (100 fg/µL) types. If the corresponding genome copy numbers were deduced, that value of LOD in weight would correspond to 1.91 × 10^5^ copies/mL (1.71 × 10^2^ cps/reaction), which seems to be a little higher with respect to the results obtained for other RNA viruses such as VHSV and infectious hematopoietic necrosis virus (IHNV) [26,27]. However, in terms of pDNA and *iv*RNA copies, the LODs obtained with this procedure (2.36 and 7.86 copies/mL, respectively) were clearly below those reported for both RNA [26,28,29,30,31] and DNA [32,33,34,35,36] fish viruses. This discrepancy is related to the large difference observed between the LOD value as crude virus RNA copies and that of the rest of standards, which was over 4 Log_10_ (from 1.17 × 10^4^ to 8.09 × 10^4^ times higher; data extracted from Table 2). This could be due to a high presence of cellular RNA, and partially to the quantity of RNA 2 that is present, which is not detected with this procedure. Moreover, this could also explain the significantly high efficiency values observed (average E = 121.2 ± 6.94).

Regarding the specificity of the procedure, both inter-group and intra-group specificity were evaluated. For the first, IPNV and VHSV were tested in this study, and no nonspecific reactions were observed at any time. The intra-group specificity, on the other hand, was demonstrated from the results obtained with crude virus as standard: similar LOD and DR, as well as reliable curves, regardless of the viral type. The reliability of the results in terms of repeatability and reproducibility was demonstrated by the very low CV values—in most cases below 5%--in all replicas and repeats (repeatability and reproducibility, respectively). Regarding reproducibility, we must say that although in this report we only present the results obtained in three different days with the same operator, other assays were performed by a second operator with lower experience, also providing CV values below 10%. These results are not shown here because three replicas were not always applied with all standards.

The amplification efficiencies (E) were quite different depending on the standard used: 120.8, 77.3, 88.4, 100.3, and 115.1 for crude and purified virus RNA (cvRNA and pvRNA, respectively), pDNA, *iv*RNA, and tissue, respectively. That is, the E values were close to or within the range considered acceptable (90–110%) using only pDNA and *iv*RNA. In similar studies with NNV, the use of only two standards was reported, providing better E values for cvRNA (E = 93.9; [20] and pDNA (E = 95−100; [14]), and similar to our results with *iv*RNA [15,24]. With other RNA viruses, better results have also been reported with pDNA [30,37,38,39], but not with *iv*RNA [29].

Several parameters can affect the efficiency of RT-qPCR reactions. One of them is the purity of the RNA template, including the presence of protein or DNA carry-over, and the template RNA/total RNA ratio. Although any of them could be involved in the high E values observed when cvRNA was used as standard, due to our experience with the method of extraction, we are extremely confident in its effectiveness. Therefore, we suspect that in the total RNA extracted there must be a large proportion of cellular RNA. Another factor is the pipet accuracy, but the low CV values obtained in our study make us reject this as a cause for efficiency failures. Finally, a poor optimization of the primer set could be an explanation to the low E values obtained with pvRNA and pDNA, and this is actually the reason for those low values. In fact, because one of our objectives was to design a universal method of quantification appropriate for any NNV type, in the previous primer pair selection assays we had to reject some sets that provided better efficiencies with some strains but that were not reliable with others. Therefore, in the end we had to condemn efficiency in favor of group specificity.

One of the main objectives of this study was to ensure the reliability of the quantification of this virus of any type, and to decide which would be the best choice of a standard to construct the calibration curve. Therefore, special emphasis was placed on evaluating the reliability of each curve for all replicas and repeats, and with all the viral types tested, and the results demonstrated the method to be reliable with any standard. Even when the results obtained with all the NNV strains tested were averaged, the standard curves were still reliable. This indicates that any standard can actually be employed.

However, it is important to point out that in some cases the comparison of quantitative results using different standards is not possible. These differences are barely shown by other authors. Considering NNV, Hick and Whittington [14] reported a difference of 2 Log_10_ in the LOD between the use of cvRNA or pDNA as standard, and Nerland et al. [40] reported a difference of 3 Log_10_ in the results using viral titer and *iv*RNA. However, there are no studies comparing quantification using different standard curves for NNV, and for other viruses they are very scarce. With VHSV, a certain parallelism between the different standard curves (using the same standards as in the present study) was observed [41]. This differs with the results obtained with NNV in the present study, since in some cases the curves intersected in low concentrations and in others in high concentrations. This is more similar to the results observed by Vázquez et al. [37], who applied RT-qPCR for the quantification of IPNV. In that case, they only tested two standards, cvRNA and *iv*RNA, and observed an intersection between curves at the lowest concentrations.

Regarding the viral quantification in infected fish tissues, and with respect to the way of evaluating this standard, other approaches than spiked tissues could have been used, such as using naturally infected fish tissues. However, a standard must be something that can be repeated in similar conditions, and using tissue from naturally infected fish would make the assay impossible to reproduce exactly in the same way. From the results obtained, it seems obvious that the dynamic range considering linear regression is shortened at the side corresponding to the lowest viral titers. To this regard, if low titers are expected, it must be considered that the quantification might be compromised if the data is extrapolated from a regression line; therefore, the use of a second degree polynomial regression curve is advised. However, as shown with the tissues from challenged fish, within the Ct range 11–35 the crude virus titer standard can be used with no significant differences in the deduced titers.

The RT-qPCR procedure was also tested in challenged fish, and all the diagnostic parameters (diagnostic sensitivity and specificity, and the predictive negative and positive values) showed the highest values possible, always higher that with virus isolation, nested PCR, or a combination of both. Furthermore, RT-qPCR was also assessed in tissues from cultured and wild fish, providing a higher number of positives than with the other detection methods.

## 5. Conclusions

The main conclusion of this study is that the RT-qPCR procedure proposed was validated for diagnosis of the disease and the simultaneous quantification of the virus. In addition, taking into account the reliability of the general standard curves (a representation of many replicas and repeats, maintaining a high level of repeatability and reproducibility), we consider that these results support the use of those general curves as reference for the quantification of any genotype of NNV.

## Figures and Tables

**Table 1:** Betanodavirus strains and genome sequences used in this study.

			Accession Number	
Strain (Genotype) *	Source	Reference	RNA 1	RNA 2
SJ93Nag (SJNNV)	Striped jack	[19]	AB056571	AB056572
SGWak97 (RGNNV)	Sevenband grouper	[19]	NC_008040	NC_008041
JFIWa98 (BFNNV)	Japanese flounder	[19]	NC_013458	NC_013459
TPKag93 (TPNNV)	Tiger puffer	[19]	NC_013460	NC_013461

* Betanodavirus strains obtained from Giusepe Bovo, Fish and Shellfish Pathology Department, OIE Reference Laboratory for Viral Encephalopathy and Retinopathy, Istituto Zooprofilattico Sperimentale delle Venezie, Viale dell’Università 10, 35020 Legnaro, PD, Italy. Viral strains are from each of the 4 genotypes: striped jack nervous necrosis virus (SJNNV), tiger puffer nervous necrosis virus (TPNNV), red-spotted grouper nervous necrosis virus (RGNNV), and barfin flounder nervous necrosis virus (BFNNV).

**Table 2:** Sensitivity and dynamic range.

LOD using as standard ^1^			**SJNNV**		**RGNNV**		**BFNNV**		**TPNNV**		**Average ^9^**
**Crude virus (RNA) ^2^**	*DR* **^8^** *:*		6		6		6		6		6
by RNA concentration (fg/µL)**^a^**			0.53		0.50		0.47		0.34		0.46 ± 0.08
As copies/mL **^b^**			2.20 × 10^5^		2.07 × 10^5^		1.94 × 10^5^		1.42 × 10^5^		1.91 × 10^5^
											
**Crude virus (Viral titer) ^3^**	*DR:*		6		6		6		6		6
As TCID_50_/mL**^c^**			9.88 × 10^0^		9.88 × 10^0^		5.56 × 10^0^		9.88 × 10^0^		8.80 ± 2.16
TCID_50_ per reaction ^d^			8.89 × 10^−2^		8.89 × 10^−2^		5.00 × 10^−2^		8.89 × 10^−2^		7.92 × 10^−2^
											
**Purified virus ^4^**	*DR:*		6		6						6
As copies/mL**^b^**			1.63 × 10^1^		1.63 × 10^1^						1.63 × 10^1^
											
**pDNA ^5^**	*DR:*				6						
As copies/mL**^b^**					2.36 × 10^0^						
											
***iv*RNA ^6^**	*DR:*				6						
As copies/mL**^b^**					7.86 × 10^0^						
											
**Virus from tissue (Viral titer) ^7^**	*DR:*				6						
As TCID_50_/g**^c^**					3.16 × 10^0^						

(**^1^**) LOD: limit of detection using as standard crude virus RNA (**^2^**) or viral titer (**^3^**), purified virus (**^4^**), plasmid DNA (**^5^**), in vitro transcribed RNA (**^6^**), or virus from infected fish tissues (**^7^**). The data are expressed as RNA concentration (fg/µL) (**^a^**), RNA, pDNA (plasmid DNA), or *iv*RNA (in vitro transcribed RNA) copies/mL (**^b^**), or TCID_50_ (per ml or per g) (**^c^**), as well as total TCID_50_ per RT-qPCR reaction (**^d^**); the copies/mL were determined by the formula γ = n/N × GL × NcMw, where γ is the amount (in g) of nucleic acid, n is the number of copies, N is the Avogadro number (6.022 × 10^23^), GL is the nucleic acid length in nucleotides, and NcMw is the average molecular weight of a nucleotide. (**^8^**) DR: the dynamic range (number of serial 10-fold dilutions yielding a reliable linear regression). (**^9^**) Values averaged from the data obtained with the different viral types tested (if more than 1): striped jack nervous necrosis virus (SJNNV), tiger puffer nervous necrosis virus (TPNNV), red-spotted grouper nervous necrosis virus (RGNNV), and barfin flounder nervous necrosis virus (BFNNV).

**Table 3:** Reliability of the quantification using crude virus as standard.

		A/SJNNV				
		Repeat 1 ^1^		Repeat 2		Repeat 3
	Viral titer ^2^	y = −2.9077x + 38.653		y = −3.0961x + 39.296		y = −3.0191x + 39.095
	RNA cps ^3^	y = −2.9077x + 51.297		y = −3.0961x + 52.760		y = −3.0191x + 52.223
		R^2^ = 0.9994^4^		R^2^ = 0.9982		R^2^ = 0.9963
		E = 120.8 ^5^		E = 110.4		E = 114.4
		General curve: Averaged from all replicas and repeats				
	Viral titer ^2^			y = −3.0076x + 39.015		
	RNA cps ^3^			y = −3.0076x + 52.093		
				R^2^ = 0.9990 ^4^/E = 115.0 ^5^		
		**B/RGNNV**				
		**Repeat 1**		**Repeat 2**		**Repeat 3**
	Viral titer	y = −2.8715x + 38.044		y = −3.068x + 38.765		y = −2.9216x + 38.497
	RNA cps	y = −2.8715x + 50.452		y = −3.068x + 52.022		y = −2.9216x + 51.122
		R^2^ = 0.9998		R^2^ = 0.9977		R^2^ = 0.9896
		E = 123.0		E = 111.8		E = 119.9
		General curve: Averaged from all replicas and repeats				
	Viral titer			y = −2.9537x + 38.435		
	RNA cps			y = −2.9537x + 51.198		
				R^2^ = 0.9976/E = 118.0		
		**C/BFNNV**				
		**Repeat 1**		**Repeat 2**		**Repeat 3**
	Viral titer	y = −2.7064x + 37.007		y = −2.8921x + 38.521		y = −2.8662x + 38.509
	RNA cps	y = −2.7064x + 49.303		y = −2.8921x + 51.661		y = −2.8662x + 51.532
		R^2^ = 0.9987		R^2^ = 0.9997		R^2^ = 0.9962
		E = 134.1		E = 121.7		E = 123.3
		General curve: Averaged from all replicas and repeats				
	Viral titer			y = −2.8216x + 38.012		
	RNA cps			y = −2.8216x + 50.832		
						
				R^2^ = 0.9993/E = 126.2		
		**D/TPNNV**				
		**Repeat 1**		**Repeat 2**		**Repeat 3**
	Viral titer	y = −2.7827x + 36.610		y = −2.9409x + 38.448		y = −2.8028x + 37.426
	RNA cps	y = −2.7827x + 48.183		y = −2.9409x + 50.679		y = −2.8028x + 49.082
		R^2^ = 0.9996		R^2^ = 0.9996		R^2^ = 0.9971
		E = 128.8		E = 118.8		E = 127.4
		General curve: Averaged from all replicas and repeats				
	Viral titer			y = −2.8421x + 37.495		
	RNA cps			y = −2.8421x + 49.315		
				R^2^ = 0.9992/E = 124.8		
		**E/VNNV (Average from the 4 types)**				
		**Repeat 1**		**Repeat 2**		**Repeat 3**
	Viral titer	y = −2.8171x + 37.606		y = −2.9993x + 38.787		y = −2.9024x + 38.415
	RNA cps	y = −2.8171x + 49.822		y = −2.9993x + 51.794		y = −2.9024x + 51.001
		R^2^ = 0.9997		R^2^ = 0.9995		R^2^ = 0.9963
		E = 126.5		E = 115.5		E = 121.1
		General curve: Averaged from the all replicas and repeats				
	Viral titer			y = −2.9063x + 38.269		
	RNA cps			y = −2.9063x + 50.873		
				R^2^ = 0.9993/E = 120.8		

(**^1^**) Regression lines averaged from 3 replicas, determined for viral titers (TCID_50_/mL) (**^2^**) and RNA copies/mL (**^3^**) values. The reliability of the curves is given by the coefficient of determination, R^2^ (**^4^**), and the efficiency of the amplification, E (**^5^**); R^2^ and E are the same in both curves, because the slopes must necessarily be the same given that both share the same threshold (Ct) values. Parts **A** to **E** show the results obtained with each VNNV (viral nervous necrosis virus) type: striped jack nervous necrosis virus (SJNNV), tiger puffer nervous necrosis virus (TPNNV), red-spotted grouper nervous necrosis virus (RGNNV), and barfin flounder nervous necrosis virus (BFNNV).

**Table 4:** Reliability of the quantification using purified virus as standard.

SJNNV				
Repeat 1 ^1^		Repeat 2		Repeat 3
y = −4.1640x + 52.354 ^2^		y = −3.9732x + 49.753		y = −4.0359x + 50.384
R^2^ = 0.9974 ^3^		R^2^ = 0.9957		R^2^ = 0.9958
E = 84.2 ^4^		E = 85.0		E = 84.5
General curve: Averaged from all replicas and repeats				
		y = −4.0577x + 50.830		
		R^2^ = 0.9965		
		E = 84.5		
**RGNNV**				
**Repeat 1**		**Repeat 2**		**Repeat 3**
y = −3.7707x + 47.46		y = −3.7431x + 47.026		y = −3.7603x + 47.502
R^2^ = 0.9913		R^2^ = 0.9938		R^2^ = 0.9847
E = 73.8		E = 78.5		E = 76.9
General curve: Averaged from all replicas and repeats				
		y = −3.7580x + 47.329		
		R^2^ = 0.9905		
		E = 76.4		
**VNNV (Averaged from both genotypes)**				
**Repeat 1**		**Repeat 2**		**Repeat 3**
y = −4.0760x + 50.727		y = −3.9559x + 49.127		y = −4.0325x + 49.958
R^2^ = 0.9970		R^2^ = 0.9969		R^2^ = 0.9942
E = 75.9		E = 79.0		E = 77.0
General curve: Averaged from all replicas and repeats				
		y = −4.0215x + 49.938		
		R^2^ = 0.9962		
		E = 77.3		

(**^1^**) Regression lines (from 3 replicas) for RNA (copies/mL) from purified virus (**^2^**). The reliability of the lines is given by the coefficient of determination R^2^ (**^3^**) and the amplification efficiency E (**^4^**). The results obtained with two VNNV (viral nervous necrosis virus) type are shown: striped jack nervous necrosis virus (SJNNV) and red-spotted grouper nervous necrosis virus (RGNNV).

**Table 5:** Reliability of the quantification using pDNA as standard.

Repeat 1 ^1^	Repeat 2	Repeat 3
y = −3.541x + 36.904 ^2^	y = −3.6852x + 37,514	y = −3.6755x + 36.923
R^2^ = 0.9991 ^3^	R^2^ = 0.9999	R^2^ = 0.9997
E = 91.6 ^4^	E = 86.8	E = 87.1
General curve: averaged from all replicas and repeats		
	y = −3.6339x + 37.114	
	R^2^ = 0.9998	
	E = 88.4	

(**^1^**) Regression lines from 3 replicas for plasmid DNA (pDNA; copies/mL) (**^2^**) values. The reliability of the lines is given by the coefficient of determination R^2^ (**^3^**) and the amplification efficiency E (**^4^**).

**Table 6:** Reliability of the quantification using *iv*RNA as standard.

Repeat 1 ^1^	Repeat 2	Repeat 3
y = −3.2887x + 36.448 ^2^	y = −3.3311x + 36.865	y = −3.3214x + 36.621
R^2^ = 0.9978 ^3^	R^2^ = 0.9965	R^2^ = 0.9974
E = 101.4 ^4^	E = 99.6	E = 100.0
General curve: averaged from all replicas and repeats		
	y = −3.3138x + 36.645	
	R^2^ = 0.9974	
	E = 100.3	

(**^1^**) Regression lines averaged from 3 replicas, determined for *iv*RNA (in vitro transcribed RNA) copies/mL (**^2^**) values. The reliability of the lines is given by the coefficient of determination R^2^ (**^3^**) and the amplification efficiency E (**^4^**).

**Table 7:** Reliability of the quantification of viruses from infected fish tissues: Assay I.

	LR-All dilutions ^1^		LR-Except lowest ^2^		PR-All dilutions ^3^
Extraction A	y = −2.2507x + 33.827		y = −2.9876x + 37.419		y = −0.4073x^2^ + 0.3502x + 31.352
	R^2^ = 0.9332 ^4^		R^2^ = 0.9967		R^2^ = 0.9943
	E = 178.2 ^5^		E = 116.1		
Extraction B	y = −2.1408x + 32.695		y = −3.0252x + 37.006		y = −0.4498x^2^ + 0.7315x + 29.962
	R^2^ = 0.8994		R^2^ = 0.9982		R^2^ = 0.9788
	E = 193.2		E = 114.1		
Extraction C	y = −2.233x + 33.392		y = −2.9400x + 36.838		y = −0.3606x^2^ + 0.0699x + 31.200
	R^2^ = 0.9391		R^2^ = 0.9999		R^2^ = 0.9880
	E = 180.4		E = 118.9		
	General curve: from all replicas and repeats. and from the 3 extractions				
	y = −2.2047x + 33.288		y = −2.8843x + 37.088		y = −0.4076x^2^ + 0.3987x + 30.810
	R^2^ = 0.9247		R^2^ = 0.9992		R^2^ = 0.9879
	E = 186.3		E = 115.1		

LR: lineal regression using the results with all the dilutions (**^1^**) or excluding the lowest one (**^2^**). PR: second degree polynomial regression using the results with all the dilutions (**^3^**). The reliability of the lines is given by the coefficient of determination R^2^ (**^4^**) and the amplification efficiency E (**^5^**).

**Table 8:** Experimentally infected fish: diagnostic characteristics of the viral diagnosis by RT-qPCR, nPCR, and cell culture isolation.

	A/ Strain: RG/SJ ^1^												B/ Strain: RG/RG ^1^								
	Species: Senegalese sole (*Solea senegalensis*)												Species: Sea bass (*Dicentrarchus labrax*)								
			**RT-qPCR** ^2^												**RT-qPCR**						
	**Sample**		**Ct** ^3^		**Titer** ^4^		**CC** ^5^		**nPCR** ^6^				**Sample**		**Ct**		**Titer**		**CC**		**nPCR**
	1		18.22		6.90		+		+				1		4.35		11.66		+		+
	2		18.28		6.88		+		+				2		7.10		10.72		+		+
	3		20.25		6.20		+		+				3		9.53		9.88		+		+
	4		22.07		5.57		+		+				4		9.98		9.73		+		+
	5		22.12		5.56		+		+				5		10.18		9.66		+		+
	6		23.27		5.16		+		+				6		12.23		8.96		+		+
	7		23.34		5.14		+		+				7		14.55		8.16		+		+
	8		23.49		5.09		+		+				8		14.67		8.12		+		+
	9		24.02		4.90		+		+				9		14.73		8.10		+		+
	10		25.28		4.47		-		+				10		15.40		7.87		+		+
	11		25.29		4.47		+		+				11		16.10		7.63		+		+
	12		25.44		4.41		+		+				12		16.32		7.55		+		+
	13		29.12		3.15		+		+				13		16.69		7.42		+		+
	14		30.60		2.64		-		+				14		16.97		7.33		+		+
	15		30.61		2.64		+		+				15		17.36		7.19		+		+
	16		31.31		2.40		-		+				16		17.97		6.98		+		+
	17		31.77		2.24		-		+				17		18.16		6.92		+		+
	18		31.85		2.21		-		+				18		18.38		6.84		+		+
	19		32.83		1.87		-		+				19		18.92		6.66		+		+
	20		33.21		1.74		-		-				20		21.67		5.71		+		+
	21		33.61		1.60		-		-				21		22.11		5.56		+		+
	22		35.11		1.09		-		-				22		25.35		4.45		+		+
	C-		-		N/A		-		-				23		27.70		3.64		+		+
	C-		-		N/A		-		-				24		28.58		3.33		+		+
	C-		-		N/A		-		-				25		29.10		3.16		-		+
	C-		-		N/A		-		-				26		29.33		3.08		+		+
													27		30.04		2.83		-		+
													28		32.03		2.15		-		-
													29		34.33		1.36		-		-
													C-		-		N/A		-		-
													C-		-		N/A		-		-
													C-		-		N/A		-		-
													C-		-		N/A		-		-
													C-		-		N/A		-		-
					
					
A/	Gold Std: All infected									B/			Gold Std: All infected								
	RT-qPCR		nPCR		CC		CC+ nPCR						RT-qPCR		nPCR		CC				CC+ nPCR
DSs^7^	1		0.591		0.864		0.864			DSs			1		0.931		0.862				0.931
DSp^8^	1		1		1		1			DSp			1		1		1				1
PPV^9^	1		1		1		1			PPV			1		1		1				1
NPV^10^	1		0.308		0.571		0.571			NPV			1		0.714		0.556				0.714

The reverse transcription real-time quantitative polymerase chain reaction (RT-qPCR) (**^2^**) procedure evaluated here was tested on tisue samples of sole (A) and sea bass (B) experimentally infected with strains of types (**^1^**) RG/SJ (red-spotted grouper nervous necrosis virus (RGNNV]/striped jack nervous necrosis virus (SJNNV)) and RG/RG, respectively. The threshold (Ct) values observed (^3^) and the viral titers (as Log_10_(TCID_50_/ml)) (^4^) deduced from the line y = −0.3438x + 13.161 (extracted from the viral titer standard curve; Table 2) are shown in comparison with isolation in cell culture (**^5^**) (+, isolated; -, not isolated), and with a previously reported nested PCR procedure [22] (+, detected; -, not detected) (**^6^**); (**^7^**), diagnostic sensitivity; (**^8^**), diagnostic specificity; (**^9^**), predictive positive value; (**^10^**), predictive negative value.

**Table 9:** Assessment of the RT-qPCR procedure in tissues from cultured fish. Comparison with nPCR and cell culture isolation.

No. of Fish	Common Name	Scientific Name	Sample	RT-qPCR ^1^	nPCR ^2^	CC ^3^
9	Turbot	*Scophthalmus maximus*	Organs/Tissues ^4^	-	-	-
32	Gilt-head seabream	*Sparus aurata*	Organs/Tissues	9+ ^5^ (Ct: 31.02 ± 3.20) ^6^ (CT: 25.02−36.61) ^7^	6+ (Ct: 25−31) 3− (Ct 34−36.61)	1+ (Ct: 25.02) 8− (Ct >25.02)
67	Gilt-head seabream	*Sparus aurata*	Blood	1 (Ct: 33)	+	-
36	Senegalese sole	*Solea senegalensis*	Organs/Tissues	8+ (Ct: 26.13 ± 10.12) (Ct: 10−34)	3+ (Ct10−22) 5− (Ct ≥ 33)	3+ (Ct 10−22) 5− (Ct ≥ 32)
262	Senegalese sole	*Solea senegalensis*	Blood	19+ (Ct: 35.68 ± 0.89) (Ct: 34−37)	-	-

^1^ The RT-PCR procedure assayed in the present study; ^2^ the nested polymerase chain reaction (nPCR) evaluated in a previous report [22]; ^3^ cell culture isolation; ^4^ organs/tissues: brain and/or kidney, spleen, and heart; ^5^ number of VNNV positive or negative samples; ^6^ average threshold (Ct) ± standard deviation; ^7^ Ct range.

## Data Availability

The data presented in this study are available on request from the corresponding author.

## Supplementary Materials

The following are available online at https://www.mdpi.com/article/10.3390/ani11041100/s1, Figure S1: Standard curves using crude virus titers, Figure S2: Standard curves using RNA copies from crude virus, Figure S3: Standard curves using RNA copies from purified virus, Figure S4: Standard curves with plasmid DNA corresponding to the RGNNV type, Figure S5: Standard curves with in vitro transcribed RNA corresponding to the RGNNV type, Figure S6: Detection and quantification of VNNV from infected fish tissues, Table S1: Primer sets tested, Table S2: List of viral strains tested, Table S3: RT-qPCR amplification of viral RNA from crude virus, Table S4: RT-qPCR amplification of viral RNA from purified virus, Table S5: qPCR amplification of pDNA, Table S6: qPCR amplification of *iv*RNA, Table S7: Detection of VNNV RNA from infected fish tissues. Table S8: Statistical differences between viral titers in experimentally infected fish tissues deduced from the Ct values observed by RT-qPCR and the different standard curves.
