# Supplementary material for: Development and Validation of a SYBR Green Real Time PCR Protocol for Detection and Quantification of Nervous Necrosis Virus (NNV) Using Different Standards
Source: Animals (Basel). 2021 Apr 12;11(4):1100. doi: 10.3390/ani11041100 (PMC8069436; doi:10.3390/ani11041100)
Supplement: Supplementary file 1 [file animals-11-01100-s001.pdf]

Supplementary Table S1.- Primer sets tested

					Type				
**ID Primer**	**Sequence (5'-3')**	**Segm**	**Position in Ref Seq[1]**		**SJ**	**RG**	**BF**	**TP**	**Rec**
**NodR1_1619**	**TCCAAAAGAAAGAAGCATAC**	**RNA1**	**1619-1638**	**sense**	√[2]	√	√	√	√
**NodR1_1758**	**TGGCATGTACCACGGAAC**	**RNA1**	**1758-1741**	**antisense**					
NodR1_1706	GCTACACCTATGCTTTCA	RNA1	1703-1720	sense	HCt	HCt	HCt	HCt	NT
NodR1_1841	GTCAGTCTCAACCARCTC	RNA1	1832-1816	antisense					
NodR1_1711	ACACCTACGCTTTCAAAG	RNA1	1706-1723	sense	HCt	HCt	HCt	HCt	NT
NodR1_1841	GTCAGTCTCAACCARCTC	RNA1	1832-1816	antisense					
RG1P4-2239F	TCTCCAGTGACATTTCTGTCTCG	RNA1	2239-2261	sense	NR	√	HCt	HCt	NT
RG1P4-2384F	TGTGTCTTAGCCCAGCCAATG	RNA1	2384-2364	antisense					
BF1P8-2602	AGCTCCTCAAGCACATTGCG	RNA1	2600-2619	sense	HCt	HCt	HCt	HCt	NT
BF1P8-2702	GACAGACATCTTCGGTTCCAAATC	RNA1	2677-2700	antisense					
SJ1P9-1926	ACAGAATTGTCCACACTATTGGG	RNA1	1924-1946	sense	√	√	NA	√	NT
SJ1P9-2075	AAGATATGAGACGAAAGCATTTGC	RNA1	2073-2050	antisense					
TP1P7-765	ATCACTGGCATTACCGCTCTG	RNA1	763-783	sense	NA	√	NA	√	NT
TP1P7-898	GGGACAATCGACACAATGTTACG	RNA1	874-896	antisense					
SJ2_coat_f	GGATTTCGTTCCATTCTCTTGGG	RNA2	735-757	sense	√	NA	NA	NR	NT
SJ2_coat_r	AATCAATGGGCAACGGTTTGTC	RNA2	825-804	antisense					
NodR2_330F	TACGCTGTTGAAACACTG	RNA2	330-347	sense	HCt	HCt	HCt	HCt	NT
NodR2_430R	CGTTGTCAGTTGGATCAG	RNA2	429-412	antisense					
NodR2F	TYCAAGCRACTCGTGGTG	RNA2	448-465	sense	√	√	√	√	HCt
NodR2R	RACACAGGAGYATCAGC	RNA2	590-572	antisense					
NodR2_1037	AAACTAACCGGGTCATCC	RNA2	1037-1054	sense	√	UA	√	√	NT
NodR2_1214	CCTCCTTGGGTGCTTTGT	RNA2	1214-1196	antisense					
RG2P12-32	TACGCAAAGGTGAGAAGAAATTGG	RNA2	31-54	sense	NA	√	√	HCt	NT
RG2P12-128	CGATTACTACGCCGACGATTG	RNA2	127-107	antisense					
BF2P14-21	CATCACAATGGTACGCAAAGGG	RNA2	20-41	sense	HCt	HCt	HCt	NA	NT
BF2P14-142	AAGGTGCATCCGCTCTCATG	RNA2	141-122	antisense					

SJ2P8-244	GGAACAGATGGAAAGATTGTCGTC	RNA2	243-266	sense	√	HCt	NA	HCt	NT
SJ2P8-374	GGGCACATTGGCTGAATTTCG	RNA2	353-373	antisense					
TP2P7-249	GGACGGATATGTCGTGGTCAAC	RNA2	248-269	sense	NR	√	UA	HCt	NT
TP2P7-350	TCCAGTGTTTCAACAATGTATCGC	RNA2	326-349	antisense					

In bold, the primer set used in this study. [1], positions in RNA 1 or 2 of strain SGwak97 (GenBank Acc # NC_008040 or NC_008041, respectively). Results obtained with the 4 type strains (SJNNV, SJ; RGNNV, RG, BFNNV, BF and TPNNV, TP) and the reassortant (Rec): [2]√, positive amplification; HCt, amplification but at high Ct values; NR, amplification not repeatable and/or reproducible; NA, no amplification; UA, unspecific amplification (more than one band); NT, not tested.

Supplementary Table S2.- List of viral strains tested

Strain	year	Isolation Source	Country	Reference	Genotype
SGWak97	1997	Sevenband grouper	Japan	Iwamoto et al., 1999	RGNNV
JFIwa98	1993	Japanese flounder	Japan	Iwamoto et al., 1999	BFNNV
TPKag93	1993	Tiger puffer	Japan	Iwamoto et al., 1999	TPNNV
SJNag93	1993	Striped jack	Japan	Iwamoto et al., 1999	SJNNV
411/96/ERV	1995	Sea bass	Italy	Cutrín et al., 2007	RGNNV
SpSa-IAusc156.03	2003	Gilthead sea bream	Spain	Olveira et al., 2009	RG/SJ
SpSs-IAusc160.03	2003	Senegalese sole	Spain	Olveira et al., 2009	RG/SJ
PtSs-IAusc573.04	2004	Senegalese sole	Portugal	Olveira et al., 2009	RG/SJ
PtSa-IAusc61.05	2005	Gilthead sea bream	Portugal	Olveira et al., 2009	RG/SJ
PtSa-IAusc74.05	2005	Gilthead sea bream	Portugal	Olveira et al., 2009	RG/SJ
SpDl-IAisc1688.08	2008	Sea bass	Spain	Olveira et al., 2009	RGNNV
SpSs-IAusc1974.08	2008	Senegalese sole	Spain	Olveira et al., 2009	RG/SJ
SpDl_IAusc965.09	2008	Sea bass	Spain	Moreno et al., 2019	RGNNV
SpMb-IAusc1544.10	2010	Red mullet	Spain	Moreno et al., 2014	RGNNV
SpPm-IAusc1586.10	2010	Turbot	Spain	Olveira et al., 2013	RGNNV
SpDl_IAusc 1043.10	2010	Sea bass	Spain	Present study	RGNNV
SpSc-IAusc1556.10	2010	Black sea bream	Spain	Moreno et al., 2014	RGNNV

SpChl-IAusc1554.01	2010	Tub gurnard	Spain	Moreno et al., 2014	RGNNV
SpDv-IAusc1549.10	2010	Two banded sea bream	Spain	Moreno et al., 2014	RGNNV
SpHd-IAusc1547.10	2010	Lusitanian toadfish	Spain	Moreno et al., 2014	RGNNV
Spwt-IAusc 230.15	2015	Sea water	Spain	Present study	RG/SJ
Spwt- IAusc 212.15	2015	Sea water	Spain	Present study	RG/SJ
Spst-IAusc 892.16	2016	Sediment	Spain	Present study	RG/SJ
SpSs-IAusc 689.16	2016	Senegalese sole	Portugal	Present study	RG/SJ
SpSa-IAusc 382.17	2017	Gilthead sea bream	Spain	Vázquez-Salgado et al, 2020	RG/SJ
SpDl-IAusc 389/I96	1996	Sea bass	Italy	Vendramin et al., 2014	RG/SJ
SpDl-IAusc 283.2009	2009	Sea bass	Italy	Panzarin et al., 2012	RGNNV
SpSs-IAus484.2.2009	2009	Senegalese sole	Spain	Panzarin et al., 2012	SJNNV
SpDl-IAus367.2.2005	2005	Sea bass	Italy	Panzarin et al., 2012	RG/SJ
VHSV Ssp11	2009	Senegalese sole	Spain	Cutrín et al., 2009	
IPNV Sp	1969	Rainbow trout	Denmark	Jørgensen y Bregnballe, 1969	

Cutrín, J.M; Dopazo, C.P.; Thiéry, R.; Leao. P.; Olveira, J.G.; Barja, J.L., Bandín, I. Emergence of pathogenic betanodaviruses belonging to the SJNNV genogroup in farmed fish species from the Iberian Peninsula. *J. Fish Dis.* **2007**, *230*, 225-232

Cutrín J.M.; Olveira, J.G.; Bandín, I.; Dopazo C.P. Validation of real time RT-PCR for diagnosis of any genotype of viral haemorrhagic septicaemia virus. *J. Virol. Meth*. **2009**, *162*, 155–162

Jørgensen, P. E. V.; Brengballe, F. Infectious pancreatic necrosis virus in rainbow trout (*Salmo gairdneri*) in Denmark. *Nord. Vet. Med* **1969**, *21*, 142-148

Iwamoto, T.; Mori, K.; Arimoto, M.; Nakai, T. High permissivity of the fish cell line SSN-1 for piscine nodaviruses. *Dis. Aquat. Organ*. **1999**, *39*, 37-47

Moreno, P.; Olveira, J.G.; Labella, A.; Cutrín, J.M.; Baro, J.C.; Borrego, J.J.; Dopazo, C.P. Surveillance of viruses in wild fish populations in areas around the Gulf of Cadiz (South Atlantic Iberian Peninsula). *Appl. Environ. Microbiol*. **2014**, *80*, 6560–6571

Moreno, P.; Souto, S.; Leiva-Rebollo, R.: Borrego, J.J.; Bandin, I.; Alonso M.C. Capsid amino acids at positions 247 and 270 are involved in the virulence of betanodaviruses to European sea bass. *Sci. Rep*. **2019**, *9*, 14068 DOI: 10.1038/s41598-019-50622-1

Olveira, J.G.; Souto, S.; Dopazo, C.P.; Thiéry, R.; Barja, J.L; Bandín, I. Comparative analysis of both genomic segments of betanodaviruses isolated from epizootic outbreaks in farmed fish species provides evidence for genetic reassortment. *J. Gen. Virol.* **2009**, *90*, 2940–2951

Olveira, J. G., Souto, S., Dopazo, C. P. & Bandín, I. Isolation of betanodavirus from farmed turbot Psetta maxima showing no signs of viral encephalopathy and retinopathy. *Aquaculture*, **2013**, *406*, 125–130

Panzarin, V.; Fusaro, A.; Monne, I.; Cappellozza, E.; Patarnello, P.; Bovo, G.; Capua, I.; Holmes, E.C.; Cattoli, G. Molecular epidemiology and evolutionary dynamics of betanodavirus in southern Europe. *Infect. Genet. Evol.* **2012,** *12*, 63–70

Vázquez-Salgado, L.; Olveira, J.G.; Dopazo, C.P.; Bandín, I. Role of rotifer (*Brachionus plicatilis*) and Artemia (*Artemia salina*) nauplii in the horizontal transmission of a natural nervous necrosis viru(NNV) reassortant strain to Senegalese sole (*Solea senegalensis*) larvae. *Vet. Quart*. **2020**, 40, 205-214, DOI: 10.1080/01652176. 2020.1810357

Vendramin, N.; Toffan, A.; Mancin, M.; Cappellozza, E.; Panzarin, V.; Bovo, G.; Cattoli, G.; Capua, I.; Terregino, C. Comparative pathogenicity study of ten different betanodavirus strains in experimentally infected European sea bass, *Dicentrarchus labrax* (L.). J. Fish Dis. **2014**, *37*, 371–383

Supplementary Table S3.- RT-qPCR amplification of viral RNA from crude virus

A/SJNNV												
**Extracted from crude virus[1]**					**$TCID_{50}$ measured**		**RT-qPCR results (data in Ct)**					
RNA/µl[2]	Abs-Extr[3]	Abs-RT[4]	Copies/ml[5]	Log ET	Viral Titer[6]	Log_{10}	Repl 1	Repl 2	Repl 3	Avg	StDev	CV
Repeat 1[7]												
1.7 ng	170.0 ng	15.3 ng	7.05×10^{11}	11.85	3.16×10^{7}	7.50	17.09	17.02	16.96	17.02	0.07	0.38
85.0 pg	8.5 ng	0.8 ng	3.53×10^{10}	10.55	1.58×10^{6}	6.20	20.50	20.23	20.40	20.38	0.14	0.67
4.3 pg	425.0 pg	38.3 pg	1.76×10^{9}	9.25	7.91×10^{4}	4.90	24.64	24.28	24.22	24.38	0.23	0.93
0.2 pg	22.0 pg	2.0 pg	8.82×10^{7}	7.95	3.95×10^{3}	3.60	28.41	28.45	28.21	28.36	0.13	0.45
10.6 fg	1.1 pg	95.4 fg	4.41×10^{6}	6.64	1.98×10^{2}	2.30	31.85	31.78	31.96	31.86	0.09	0.28
0.53 fg	53.0 fg	4.8 fg	2.20×10^{5}	5.34	9.88×10^{0}	0.99	35.05	36.28	36.12	35.82	0.67	1.87
Repeat 2												
1.7 ng	170.0 ng	15.3 ng	7.05×10^{11}	11.85	3.16×10^{7}	7.50	16.09	16.25	16.24	16.19	0.09	0.55
85.0 pg	8.5 ng	0.8 ng	3.53×10^{10}	10.55	1.58×10^{6}	6.20	20.31	20.29	20.25	20.28	0.03	0.15
4.3 pg	425.0 pg	38.3 pg	1.76×10^{9}	9.25	7.91×10^{4}	4.90	24.05	23.96	23.79	23.93	0.13	0.55
0.2 pg	22.0 pg	2.0 pg	8.82×10^{7}	7.95	3.95×10^{3}	3.60	27.50	27.59	27.79	27.63	0.15	0.54
10.6 fg	1.1 pg	95.4 fg	4.41×10^{6}	6.64	1.98×10^{2}	2.30	33.04	32.53	32.07	32.55	0.49	1.49
0.5 fg	53.0 fg	4.8 fg	2.20×10^{5}	5.34	9.88×10^{0}	0.99	35.70	37.12	36.06	36.29	0.74	2.03
Repeat 3												
1.7 ng	170.0 ng	15.3 ng	7.05×10^{11}	11.85	3.16×10^{7}	7.50	16.54	16.86	17.29	16.90	0.38	2.23
85.0 pg	8.5 ng	0.8 ng	3.53×10^{10}	10.55	1.58×10^{6}	6.20	20.14	20.74	20.63	20.50	0.32	1.56
4.3 pg	425.0 pg	38.3 pg	1.76×10^{9}	9.25	7.91×10^{4}	4.90	23.78	23.52	23.68	23.66	0.13	0.55
0.2 pg	22.0 pg	2.0 pg	8.82×10^{7}	7.95	3.95×10^{3}	3.60	27.88	27.59	28.11	27.86	0.26	0.94
10.6 fg	1.1 pg	95.4 fg	4.41×10^{6}	6.64	1.98×10^{2}	2.30	32.25	31.87	32.27	32.13	0.23	0.70
0.5 fg	53.0 fg	4.8 fg	2.20×10^{5}	5.34	9.88×10^{0}	0.99	35.18	35.68	38.87	36.58	2.00	*5.47*
Average data for SJNNV												
1.7 ng	170.0 ng	15.3 ng	7.05×10^{11}	11.85	3.16×10^{7}	7.50		16.70			0.43	2.60
85.0 pg	8.5 ng	0.8 ng	3.53×10^{10}	10.55	1.58×10^{6}	6.20		20.39			0.20	0.98
4.3 pg	425.0 pg	38.3 pg	1.76×10^{9}	9.25	7.91×10^{4}	4.90		23.99			0.35	1.45

0.2 pg	22.0 pg	2.0 pg	8.82×10^{7}	7.95	3.95×10^{3}	3.60	27.95	0.36	1.29
10.6 fg	1.1 pg	95.4 fg	4.41×10^{6}	6.64	1.98×10^{2}	2.30	32.18	0.40	1.25
0.5 fg	53.0 fg	4.8 fg	2.20×10^{5}	5.34	9.88×10^{0}	0.99	36.23	1.17	3.22

Supplementary Table 2 (Cont.)

B/RGNNV												
**Extracted from crude virus[1]**					**$TCID_{50}$ measured**		**RT-qPCR results (data in Ct)**					
RNA/µl[2]	Abs-Extr[3]	Abs-RT[4]	Copies/ml[5]	Log ET	Viral Titer[6]	Log_{10}	Repl 1	Repl 2	Repl 3	Avg	StDev	CV
Repeat 1[7]												
1.6 ng	160.0 ng	14.4 ng	6.62×10^{11}	11.82	3.16×10^{7}	7.50	16.56	16.61	16.69	16.62	0.07	0.39
80.0 pg	8.0 ng	0.7 ng	3.31×10^{10}	10.55	1.58×10^{6}	6.20	20.18	20.18	19.87	20.08	0.18	0.89
4.0 pg	400.0 pg	36.0 pg	1.66×10^{9}	9.22	7.91×10^{4}	4.90	23.98	24.11	23.8	23.96	0.16	0.65
0.2 pg	20.0 pg	1.8 pg	8.28×10^{7}	7.92	3.95×10^{3}	3.60	28.00	27.82	27.63	27.82	0.19	0.67
10.0 fg	1.0 pg	90.0 fg	4.14×10^{6}	6.62	1.98×10^{2}	2.30	31.57	31.32	31.31	31.40	0.15	0.47
0.5 fg	50.0 fg	4.5 fg	2.07×10^{5}	5.32	9.88×10^{0}	0.99	34.46	35.62	35.54	35.21	0.65	1.84
Repeat 2												
1.6 ng	160.0 ng	14.4 ng	6.62×10^{11}	11.82	3.16×10^{7}	7.50	16.01	15.95	16.31	16.09	0.19	1.20
80.0 pg	8.0 ng	0.7 ng	3.31×10^{10}	10.55	1.58×10^{6}	6.20	19.85	19.75	19.69	19.76	0.08	0.41
4.0 pg	400.0 pg	36.0 pg	1.66×10^{9}	9.22	7.91×10^{4}	4.90	23.44	23.71	23.43	23.53	0.16	0.68
0.2 pg	20.0 pg	1.8 pg	8.28×10^{7}	7.92	3.95×10^{3}	3.60	27.36	27.08	27.16	27.20	0.14	0.53
10.0 fg	1.0 pg	90.0 fg	4.14×10^{6}	6.62	1.98×10^{2}	2.30	31.46	31.90	31.63	31.66	0.22	0.70
0.5 fg	50.0 fg	4.5 fg	2.07×10^{5}	5.32	9.88×10^{0}	0.99	35.79	36.73	35.95	36.16	0.50	1.39
Repeat 3												
1.6 ng	160.0 ng	14.4 ng	6.62×10^{11}	11.82	3.16×10^{7}	7.50	18.27	16.53	16.4	17.07	1.04	6.12
80.0 pg	8.0 ng	0.7 ng	3.31×10^{10}	10.55	1.58×10^{6}	6.20	19.91	21.75	20.96	20.87	0.92	4.42
4.0 pg	400.0 pg	36.0 pg	1.66×10^{9}	9.22	7.91×10^{4}	4.90	23.29	23.51	23.25	23.35	0.14	0.60
0.2 pg	20.0 pg	1.8 pg	8.28×10^{7}	7.92	3.95×10^{3}	3.60	27.1	27.15	27.05	27.10	0.05	0.18
10.0 fg	1.0 pg	90.0 fg	4.14×10^{6}	6.62	1.98×10^{2}	2.30	31.24	32.2	31.72	31.72	0.48	1.51
0.5 fg	50.0 fg	4.5 fg	2.07×10^{5}	5.32	9.88×10^{0}	0.99	37.4	35.77	36.08	36.42	0.87	2.38
Average data for SJNNV												
1.6 ng	160.0 ng	14.4 ng	6.62×10^{11}	11.82	3.16×10^{7}	7.50		16.59			0.68	4.10
80.0 pg	8.0 ng	0.7 ng	3.31×10^{10}	10.55	1.58×10^{6}	6.20		20.24			0.68	3.38
4.0 pg	400.0 pg	36.0 pg	1.66×10^{9}	9.22	7.91×10^{4}	4.90		23.61			0.30	1.28
0.2 pg	20.0 pg	1.8 pg	8.28×10^{7}	7.92	3.95×10^{3}	3.60		27.37			0.36	1.30
10.0 fg	1.0 pg	90.0 fg	4.14×10^{6}	6.62	1.98×10^{2}	2.30		31.59			0.31	0.99
0.5 fg	50.0 fg	4.5 fg	2.07×10^{5}	5.32	9.88×10^{0}	0.99		35.93			0.81	2.26

Supplementary Table 2 (Cont.)

C/BFNNV												
**Extracted from crude virus[1]**					**$TCID_{50}$ measured**		**RT-qPCR results (data in Ct)**					
RNA/µl[2]	Abs-Extr[3]	Abs-RT[4]	Copies/ml[5]	Log ET	Viral Titer[6]	Log_{10}	Repl 1	Repl 2	Repl 3	Avg	StDev	CV
Repeat 1[7]												
1.5 ng	150.0 ng	13.5 ng	6.22×10^{11}	11.79	1.78×10^{7}	7.25	17.7	17.33	17.38	17.47	0.20	1.15
75 pg	7.5 ng	0.7 ng	3.11×10^{10}	10.49	8.89×10^{5}	5.95	20.63	20.66	20.59	20.63	0.04	0.17
3.75 pg	375.0 pg	33.8 pg	1.55×10^{9}	9.19	4.45×10^{4}	4.65	24.43	24.47	24.46	24.45	0.02	0.09
0.18 pg	18.0 pg	1.6 pg	7.77×10^{7}	7.89	2.22×10^{3}	3.35	28.22	28.04	28.08	28.11	0.09	0.34
9.38 fg	938.0 fg	84.4 fg	3.89×10^{6}	6.59	1.11×10^{2}	2.05	31.91	31.58	31.77	31.75	0.17	0.52
0.47 fg	47.0 fg	4.2 fg	1.94×10^{5}	5.29	5.56×10^{0}	0.74	34.89	33.93	35.31	34.71	0.71	2.04
Repeat 2												
1.5 ng	150.0 ng	13.5 ng	6.22×10^{11}	11.79	1.78×10^{7}	7.25	18.05	17.49	17.51	17.68	0.32	1.80
75 pg	7.5 ng	0.7 ng	3.11×10^{10}	10.49	8.89×10^{5}	5.95	21.29	21.29	21.23	21.27	0.03	0.16
3.75 pg	375.0 pg	33.8 pg	1.55×10^{9}	9.19	4.45×10^{4}	4.65	24.97	25.12	25.05	25.05	0.08	0.30
0.18 pg	18.0 pg	1.6 pg	7.77×10^{7}	7.89	2.22×10^{3}	3.35	28.84	28.66	28.56	28.69	0.14	0.49
9.38 fg	938.0 fg	84.4 fg	3.89×10^{6}	6.59	1.11×10^{2}	2.05	32.46	32.09	33.05	32.53	0.48	1.49
0.47 fg	47.0 fg	4.2 fg	1.94×10^{5}	5.29	5.56×10^{0}	0.74	36.04	35.67	37.9	36.54	1.20	3.27
Repeat 3												
1.5 ng	150.0 ng	13.5 ng	6.22×10^{11}	11.79	1.78×10^{7}	7.25	19.27	17.77	17.89	18.31	0.83	4.55
75 pg	7.5 ng	0.7 ng	3.11×10^{10}	10.49	8.89×10^{5}	5.95	21.23	20.91	21.71	21.28	0.40	1.89
3.75 pg	375.0 pg	33.8 pg	1.55×10^{9}	9.19	4.45×10^{4}	4.65	23.98	24.86	24.91	24.58	0.52	2.13
0.18 pg	18.0 pg	1.6 pg	7.77×10^{7}	7.89	2.22×10^{3}	3.35	28.56	28.52	29.02	28.70	0.28	0.97
9.38 fg	938.0 fg	84.4 fg	3.89×10^{6}	6.59	1.11×10^{2}	2.05	32.22	32.5	33.36	32.69	0.59	1.82
0.47 fg	47.0 fg	4.2 fg	1.94×10^{5}	5.29	5.56×10^{0}	0.74	36.3	37.54	36.39	36.74	0.69	1.88
Average data for SJNNV												
1.5 ng	150.0 ng	13.5 ng	6.22×10^{11}	11.79	1.78×10^{7}	7.25		17.82			0.59	3.33
75 pg	7.5 ng	0.7 ng	3.11×10^{10}	10.49	8.89×10^{5}	5.95		21.06			0.38	1.82
3.75 pg	375.0 pg	33.8 pg	1.55×10^{9}	9.19	4.45×10^{4}	4.65		24.69			0.38	1.53
0.18 pg	18.0 pg	1.6 pg	7.77×10^{7}	7.89	2.22×10^{3}	3.35		28.50			0.33	1.17
9.38 fg	938.0 fg	84.4 fg	3.89×10^{6}	6.59	1.11×10^{2}	2.05		32.33			0.59	1.81
0.47 fg	47.0 fg	4.2 fg	1.94×10^{5}	5.29	5.56×10^{0}	0.74		36.00			1.24	3.45

Supplementary Table 2 (Cont.)

D/TPNNV												
**Extracted from crude virus[1]**					**$TCID_{50}$ measured**		**RT-qPCR results (data in Ct)**					
RNA/µl[2]	Abs-Extr[3]	Abs-RT[4]	Copies/ml[5]	Log_{10}	Viral Titer[6]	Log CT	Repl 1	Repl 2	Repl 3	Avg	StDev	CV
Repeat 1[7]												
1.1 ng	110.0 ng	9.9 ng	4.56×10^{11}	11.66	3.16×10^{7}	7.50	15.83	15.84	15.54	15.74	0.17	1.08
55 pg	5.5 ng	0.5 ng	2.28×10^{10}	10.36	1.58×10^{6}	6.20	19.35	19.29	19.37	19.34	0.04	0.22
2.75 pg	275.0 pg	24.8 pg	1.14×10^{9}	9.06	7.91×10^{4}	4.90	22.97	22.98	22.74	22.90	0.14	0.59
0.14 pg	14.0 pg	1.3 pg	5.70×10^{7}	7.76	3.95×10^{3}	3.60	26.87	26.56	26.57	26.67	0.18	0.66
6.88 fg	688.0 fg	67.9 fg	2.85×10^{6}	6.45	1.98×10^{2}	2.30	30.17	30.64	30.56	30.46	0.25	0.83
0.34 fg	34.0 fg	3.1 fg	1.42×10^{5}	5.15	9.88×10^{0}	0.99	34.45	33.09	33.42	33.65	0.71	2.11
Repeat 2												
1.1 ng	110.0 ng	9.9 ng	4.56×10^{11}	11.66	3.16×10^{7}	7.50	16.05	16.48	16.4	16.31	0.23	1.40
55 pg	5.5 ng	0.5 ng	2.28×10^{10}	10.36	1.58×10^{6}	6.20	20.24	20.24	20.26	20.25	0.01	0.06
2.75 pg	275.0 pg	24.8 pg	1.14×10^{9}	9.06	7.91×10^{4}	4.90	23.92	24.12	24.06	24.03	0.10	0.43
0.14 pg	14.0 pg	1.3 pg	5.70×10^{7}	7.76	3.95×10^{3}	3.60	27.94	28.06	27.79	27.93	0.14	0.48
6.88 fg	688.0 fg	67.9 fg	2.85×10^{6}	6.45	1.98×10^{2}	2.30	31.86	31.78	32.07	31.90	0.15	0.47
0.34 fg	34.0 fg	3.1 fg	1.42×10^{5}	5.15	9.88×10^{0}	0.99	34.27	34.95	36.74	35.32	1.28	3.61
Repeat 3												
1.1 ng	110.0 ng	9.9 ng	4.56×10^{11}	11.66	3.16×10^{7}	7.50	17.28	16.45	16.31	16.68	0.52	3.14
55 pg	5.5 ng	0.5 ng	2.28×10^{10}	10.36	1.58×10^{6}	6.20	19.81	19.86	20.2	19.96	0.21	1.06
2.75 pg	275.0 pg	24.8 pg	1.14×10^{9}	9.06	7.91×10^{4}	4.90	23.31	23.31	23.34	23.32	0.02	0.07
0.14 pg	14.0 pg	1.3 pg	5.70×10^{7}	7.76	3.95×10^{3}	3.60	27.07	27.16	27.11	27.11	0.05	0.17
6.88 fg	688.0 fg	67.9 fg	2.85×10^{6}	6.45	1.98×10^{2}	2.30	31.44	31.51	31.82	31.59	0.20	0.64
0.34 fg	34.0 fg	3.1 fg	1.42×10^{5}	5.15	9.88×10^{0}	0.99	34.49	34.03	34.88	34.47	0.43	1.23
Average data for SJNNV												
1.1 ng	110.0 ng	9.9 ng	4.56×10^{11}	11.66	3.16×10^{7}	7.50		16.24			0.51	3.13
55 pg	5.5 ng	0.5 ng	2.28×10^{10}	10.36	1.58×10^{6}	6.20		19.85			0.42	2.10
2.75 pg	275.0 pg	24.8 pg	1.14×10^{9}	9.06	7.91×10^{4}	4.90		23.42			0.50	2.16
0.14 pg	14.0 pg	1.3 pg	5.70×10^{7}	7.76	3.95×10^{3}	3.60		27.24			0.57	2.08
6.88 fg	688.0 fg	67.9 fg	2.85×10^{6}	6.45	1.98×10^{2}	2.30		31.32			0.68	2.18
0.34 fg	34.0 fg	3.1 fg	1.42×10^{5}	5.15	9.88×10^{0}	0.99		34.48			1.05	3.04

Supplementary Table 2 (Cont.)

E/General Standard Curve (average of the 4 strains)												
**Extracted from crude virus[1]**					**$TCID_{50}$ measured**		**RT- qPCR results (data in Ct)**					
RNA/µl[2]	Abs-Extr[3]	Abs-RT[4]	Copies/ml[5]	Log ET	Viral Titer[6]	Log_{10}	Repl 1	Repl 2	Repl 3	Avg	StDev	CV
Repeat 1												
1.48 ng	148.0 ng	13.3 ng	6.11×10^{11}	11.79	2.82×10^{7}	7.45	16.57	16.57	16.43	16.52	0.08	0.51
73.75 pg	7.4 ng	0.7 ng	3.06×10^{10}	10.49	1.41×10^{6}	6.15	20.09	19.98	19.94	20.00	0.07	0.37
3.69 pg	369.0 pg	33.2 pg	1.53×10^{9}	9.18	7.04×10^{4}	4.85	23.86	23.75	23.63	23.75	0.12	0.49
0.18 pg	18.0 pg	1.6 pg	7.64×10^{7}	7.88	3.52×10^{3}	3.55	27.67	27.47	27.45	27.53	0.12	0.44
9.22 fg	922.0 fg	83.0 fg	3.82×10^{6}	6.58	1.76×10^{2}	2.25	31.13	31.05	31.21	31.13	0.08	0.25
0.46 fg	46.0 fg	4.1 fg	1.91×10^{5}	5.28	8.80×10^{0}	0.94	34.60	34.71	34.93	34.75	0.17	0.48
Repeat 2												
1.48 ng	148.0 ng	13.3 ng	6.11×10^{11}	11.79	2.82×10^{7}	7.45	16.32	16.41	16.33	16.36	0.05	0.29
73.75 pg	7.4 ng	0.7 ng	3.06×10^{10}	10.49	1.41×10^{6}	6.15	20.30	20.22	20.15	20.22	0.07	0.36
3.69 pg	369.0 pg	33.2 pg	1.53×10^{9}	9.18	7.04×10^{4}	4.85	23.96	24.00	23.93	23.96	0.04	0.15
0.18 pg	18.0 pg	1.6 pg	7.64×10^{7}	7.88	3.52×10^{3}	3.55	27.72	27.64	27.80	27.72	0.08	0.28
9.22 fg	922.0 fg	83.0 fg	3.82×10^{6}	6.58	1.76×10^{2}	2.25	32.01	31.92	32.06	32.00	0.07	0.23
0.46 fg	46.0 fg	4.1 fg	1.91×10^{5}	5.28	8.80×10^{0}	0.94	35.27	35.97	36.31	35.85	0.53	1.48
Repeat 3												
1.48 ng	148.0 ng	13.3 ng	6.11×10^{11}	11.79	2.82×10^{7}	7.45	17.39	16.57	16.77	16.91	0.43	2.53
73.75 pg	7.4 ng	0.7 ng	3.06×10^{10}	10.49	1.41×10^{6}	6.15	20.21	20.68	20.66	20.52	0.26	1.29
3.69 pg	369.0 pg	33.2 pg	1.53×10^{9}	9.18	7.04×10^{4}	4.85	23.72	23.66	23.72	23.70	0.03	0.15
0.18 pg	18.0 pg	1.6 pg	7.64×10^{7}	7.88	3.52×10^{3}	3.55	27.51	27.53	27.66	27.57	0.08	0.29
9.22 fg	922.0 fg	83.0 fg	3.82×10^{6}	6.58	1.76×10^{2}	2.25	31.61	31.93	31.98	31.84	0.20	0.64
0.46 fg	46.0 fg	4.1 fg	1.91×10^{5}	5.28	8.80×10^{0}	0.94	35.52	35.39	36.43	35.78	0.57	1.58
Average data for VNNV												
1.48 ng	148.0 ng	13.3 ng	6.11×10^{11}	11.79	2.82×10^{7}	7.45		16.60			0.33	2.00
73.75 pg	7.4 ng	0.7 ng	3.06×10^{10}	10.49	1.41×10^{6}	6.15		20.25			0.26	1.31
3.69 pg	369.0 pg	33.2 pg	1.53×10^{9}	9.18	7.04×10^{4}	4.85		23.80			0.14	0.57
0.18 pg	18.0 pg	1.6 pg	7.64×10^{7}	7.88	3.52×10^{3}	3.55		27.60			0.12	0.43
9.22 fg	922.0 fg	83.0 fg	3.82×10^{6}	6.58	1.76×10^{2}	2.25		31.66			0.42	1.32
0.46 fg	46.0 fg	4.1 fg	1.91×10^{5}	5.28	8.80×10^{0}	0.94		35.46			0.67	1.88

Crude virus was subjected to RNA extraction ([1]); the RNA concentration was measured and serial dilutions subjected to RT-qPCR as described. The quantity of RNA employed is expressed as RNA concentration per µl ([2]), and in absolute terms as the total RNA per volume subjected to extraction ([3]) and per volume subjected to the RT step ([4]). Additionally, the concentration in terms of copies/ml ([5]) was extrapolated from the formula $\gamma = n/N \times GL \times NcMw$, where γ is the amount (in g) of viral RNA, n is the number of genomic RNA molecules (genome copies), N is the Avogadro number (6.022×10^{23}), GL is the genome length in nucleotides, and NcMw is the average molecular weight of a nucleotide (350.5 Da).

Moreover, the same sample of crude virus was subjected to viral titration, and thus the corresponding viral titers are shown as $TCID_{50}$/ml ([6]) and Log_{10} of that value. The study was performed with the 4 type strains, SJNNV (A), RGNNV (B), BFNNV (C) and RPNNV (D). Table E shows the results obtained averaging the data from the 4 viral types. ([7]) Data for each repeat are the average from 3 replicas.

Supplementary Table S4.- RT-qPCR amplification of viral RNA from purified virus

A/SJNNV

**Purified virus RNA[1]**		**RT-qPCR results (data in Ct)**					
copies/ml[2]	Log	Repl 1	Repl 2	Repl 3	Avg	StDev	CV
			Repeat 1[3]				
5.21×10^{9}	9.72	12.15	12.14	12.50	12.26	0.20	1.67
2.61×10^{8}	8.42	17.02	16.83	17.02	16.96	0.11	0.67
1.30×10^{7}	7.12	22.39	22.27	22.34	22.33	0.06	0.28
6.52×10^{5}	5.81	28.07	28.08	28.11	28.09	0.02	0.07
3.26×10^{4}	4.51	33.99	34.65	34.65	34.43	0.38	1.10
1.63×10^{3}	3.21	38.38	39.02	38.25	38.55	0.41	1.07
			Repeat 2				
5.21×10^{9}	9.72	11.58	11.26	11.22	11.35	0.20	1.75
2.61×10^{8}	8.42	15.96	15.65	16.23	15.95	0.29	1.82
1.30×10^{7}	7.12	21.10	21.13	21.03	21.09	0.06	0.26
6.52×10^{5}	5.81	27.05	27.04	26.72	26.94	0.19	0.69
3.26×10^{4}	4.51	33.05	32.65	32.81	32.84	0.20	0.62
1.63×10^{3}	3.21	36.42	36.28	36.000	36.24	0.22	0.59
			Repeat 3				
5.21×10^{9}	9.72	11.14	11.93	12.04	11.70	0.49	4.17
2.61×10^{8}	8.42	15.44	16.06	16.19	15.89	0.40	2.52
1.30×10^{7}	7.12	20.97	21.05	21.17	21.06	0.10	0.49
6.52×10^{5}	5.81	28.18	26.46	26.57	27.07	0.96	3.55
3.26×10^{4}	4.51	32.69	33.52	33.10	33.10	0.42	1.26
1.63×10^{3}	3.21	37.64	36.74	36.42	36.93	0.63	1.72
			Total				
5.21×10^{9}	9.72		11.77			0.49	4.13
2.61×10^{8}	8.42		16.27			0.58	3.54
1.30×10^{7}	7.12		21.49			0.63	2.93
6.52×10^{5}	5.81		27.37			0.73	2.68
3.26×10^{4}	4.51		33.46			0.80	2.38
1.63×10^{3}	3.21		37.24			1.10	2.95

Supplementary Table S3 (Cont.)

		B/RGNNV					
**Purified virus RNA[1]**		**RT-qPCR results (data in Ct)**					
copies/ml[2]	Log	Repl 1	Repl 2	Repl 3	Avg	StDev	CV
			Repeat 1[3]				
5.21×10^{9}	9.72	10.54	10.69	10.16	10.47	0.27	2.59
2.61×10^{8}	8.42	14.89	15.03	14.83	14.92	0.10	0.70
1.30×10^{7}	7.12	20.94	20.44	20.34	20.58	0.32	1.56
6.52×10^{5}	5.81	27.27	26.52	26.53	26.77	0.43	1.59
3.26×10^{4}	4.51	32.00	31.86	31.26	31.71	0.39	1.23
1.63×10^{3}	3.21	35.33	36.27	34.82	35.47	0.74	2.07
8.15×10^{1}	1.91	39.03	39.03	38.45	38.84	0.34	0.87
			Repeat 2				
5.21×10^{9}	9.72	10.00	10.87	10.69	10.52	0.46	4.38
2.61×10^{8}	8.42	14.69	15.02	14.93	14.88	0.17	1.12
1.30×10^{7}	7.12	20.12	20.12	20.18	20.14	0.03	0.17
6.52×10^{5}	5.81	26.36	25.74	26.19	26.10	0.32	1.23
3.26×10^{4}	4.51	31.43	30.94	31.25	31.21	0.25	0.81
1.63×10^{3}	3.21	36.40	34.81	35.00	35.40	0.87	2.45
8.15×10^{1}	1.91	39.03	38.75	38.02	38.60	0.53	1.36
			Repeat 3				
5.21×10^{9}	9.72	10.28	10.38	10.11	10.26	0.14	1.32
2.61×10^{8}	8.42	15.13	15.29	15.06	15.16	0.12	0.77
1.30×10^{7}	7.12	20.33	20.62	20.72	20.56	0.20	0.97
6.52×10^{5}	5.81	27.02	26.99	27.68	27.23	0.39	1.43
3.26×10^{4}	4.51	32.81	32.39	32.19	32.46	0.32	0.98
1.63×10^{3}	3.21	36.71	34.31	34.68	35.24	1.29	3.67
8.15×10^{1}	1.91	39.20	38.00	38.50	38.57	0.60	1.56
			Average data from the 3 repeats				
5.21×10^{9}	9.72		10.41			0.30	2.88
2.61×10^{8}	8.42		14.99			0.17	1.16
1.30×10^{7}	7.12		20.42			0.29	1.40
6.52×10^{5}	5.81		26.70			0.60	2.23
3.26×10^{4}	4.51		31.79			0.62	1.94
1.63×10^{3}	3.21		35.37			0.87	2.45
8.15×10^{1}	1.91		38.67			0.45	1.17

Purified virus of types SJNNV (A) and RGNNV (B) was subjected to RNA extraction ([1]); the RNA concentration was measured and serial dilutions subjected to RT-qPCR as described. The concentration in terms of copies/ml ([2]) was extrapolated from the formula $\gamma = n/N \times GL \times NcMw$, where γ is the

amount (in g) of viral RNA, n is the number of genomic RNA molecules (genome copies), N is the Avogadro number (6.022×10^{23}), GL is the genome length in nucleotides, and NcMw is the average molecular weight of a nucleotide (350.5 Da). ([3]) Data for repeats are the average from 3 replicas.

Supplementary Table S5.- qPCR amplification of pDNA

pDNA[1]		qPCR results (data in Ct)					
copies/ml[2]	Log	Repl 1	Repl 2	Repl 3	Avg	StDev	CV
Repeat 1[3]							
7.55×10^{6}	6.88	12.17	12.56	12.13	12.29	0.24	1.93
3.77×10^{5}	5.58	17.27	17.25	17.13	17.22	0.08	0.44
1.89×10^{4}	4.28	21.86	21.91	22.2	21.99	0.18	0.83
9.43×10^{2}	2.97	26.34	26.49	26.47	26.43	0.08	0.31
4.72×10^{1}	1.67	31.18	31.41	31.16	31.25	0.14	0.44
2.36×10^{0}	0.37	36.16	34.76	34.76	35.23	0.81	2.29
Repeat 2							
7.55×10^{6}	6.88	12.06	12.12	12.03	12.07	0.05	0.38
3.77×10^{5}	5.58	17.05	17.23	17	17.09	0.12	0.71
1.89×10^{4}	4.28	22.38	21.38	21.66	21.81	0.52	2.37
9.43×10^{2}	2.97	26.4	26.62	26.58	26.53	0.12	0.44
4.72×10^{1}	1.67	31.16	30.98	31.49	31.21	0.26	0.83
2.36×10^{0}	0.37	36.25	36.14	36.26	36.22	0.07	0.18
Repeat 3							
7.55×10^{6}	6.88	11.39	11.49	11.5	11.46	0.06	0.53
3.77×10^{5}	5.58	16.77	16.34	16.59	16.57	0.22	1.30
1.89×10^{4}	4.28	21.21	21.65	21.29	21.38	0.23	1.10
9.43×10^{2}	2.97	25.62	26.05	25.98	25.88	0.23	0.89
4.72×10^{1}	1.67	30.88	30.64	30.9	30.81	0.14	0.47
2.36×10^{0}	0.37	36.42	34.73	35.32	35.49	0.86	2.42
Average data from the 3 repeats							
7.55×10^{6}	6.88		11.94			0.39	3.28
3.77×10^{5}	5.58		16.96			0.33	1.92
1.89×10^{4}	4.28		21.73			0.40	1.85
9.43×10^{2}	2.97		26.28			0.33	1.26
4.72×10^{1}	1.67		31.09			0.27	0.86
2.36×10^{0}	0.37		35.64			0.74	2.07

A plasmid DNA ([1]pDNA) constructed with a 3067 pb nucleotide fragment from genomic RNA 1 of strain SGWak97 (RGNNV) was subjected to serial dilutions and qPCR as described. The concentration in terms of copies/ml ([2]) was extrapolated from the formula $\gamma = n/N \times GL \times NcMw$, where γ is the amount (in g) of viral RNA, n

is the number of genomic RNA molecules (genome copies), N is the Avogadro number (6.022×10^{23}), and being GL=3067 nc, and NcMw (the average molecular weight of each pair of nucleotides) estimated as 660 g/mol. [3]Data for repeats are the average from 3 replicas.

Supplementary Table S6.- qPCR amplification of ivRNA

tRNA[1]		qPCR results (data in Ct)					
copies/ml[2]	Log	Repl 1	Repl 2	Repl 3	Avrg	StDev	CV
		Repeat 1[3]					
2.52×10^{7}	6.88	12.61	12.75	12.67	12.68	0.07	0.55
1.26×10^{6}	5.58	15.97	15.96	16.00	15.98	0.02	0.13
6.29×10^{4}	4.28	20.32	20.54	20.36	20.41	0.12	0.57
3.14×10^{3}	2.97	24.74	24.33	25.04	24.70	0.36	1.44
1.57×10^{2}	1.67	29.50	29.33	29.06	29.30	0.22	0.76
7.86×10^{0}	0.37	33.83	33.24	34.26	33.78	0.51	1.52
		Repeat 2					
2.52×10^{7}	7.40	12.92	13.02	12.84	12.93	0.09	0.70
1.26×10^{6}	6.10	16.06	16.16	16.09	16.10	0.05	0.32
6.29×10^{4}	4.80	20.68	20.31	20.52	20.50	0.19	0.91
3.14×10^{3}	3.50	25.01	24.80	24.94	24.92	0.11	0.43
1.57×10^{2}	2.20	29.52	29.53	29.40	29.48	0.07	0.25
7.86×10^{0}	0.90	34.14	34.50	34.42	34.35	0.19	0.55
		Repeat 3					
2.52×10^{7}	7.40	12.08	12.85	13.01	12.65	0.50	3.93
1.26×10^{6}	6.10	16.00	15.82	15.83	15.88	0.10	0.64
6.29×10^{4}	4.80	20.17	20.44	20.63	20.41	0.23	1.13
3.14×10^{3}	3.50	24.62	24.78	24.78	24.73	0.09	0.37
1.57×10^{2}	2.20	29.53	29.71	29.49	29.58	0.12	0.40
7.86×10^{0}	0.90	34.20	33.47	33.78	33.82	0.37	1.08
			Total				
2.52×10^{7}	7.40		12.75			0.29	2.26
1.26×10^{6}	6.10		15.99			0.11	0.70
6.29×10^{4}	4.80		20.44			0.17	0.81
3.14×10^{3}	3.50		24.78			0.22	0.87
1.57×10^{2}	2.20		29.45			0.18	0.61
7.86×10^{0}	0.90		33.98			0.43	1.27

A RNA transcript ([1]) (ivRNA was generated from the plasmid DNA, its concentration calculated ([2]copies/ml), and RT-qPCR applied as described. [3]Data for repeats are the average from 3 replicas.

Supplementary Table S7.- Detection and quantification of VNNV from infected fish tissues

Assay I: RNA extraction A[1]							
Viral titer[2]		RT-qPCR results (data in Ct)[3]					
$TCID_{50}$/gr	Log	Repl 1	Repl 2	Repl 3	Avrg[4]	StDev	CV
Repeat 1							
7.91×10^5	5.90	19.66	19.86	20.55	20.02	0.47	2.33
1.58×10^5	5.20	21.64	22.98	20.82	21.81	1.09	5.00
1.58×10^4	4.20	26.02	25.63	26.45	26.03	0.41	1.58
1.58×10^2	2.20	30.75	30.91	30.62	30.76	0.15	0.47
3.16×10^0	0.50	30.76	30.26	30.17	30.40	0.32	1.05
Repeat 2							
7.91×10^5	5.90	18.26	18.08	18.17	18.17	0.09	0.50
1.58×10^5	5.20	20.63	20.47	21.78	20.96	0.72	3.41
1.58×10^4	4.20	23.2	23.04	23.01	23.08	0.10	0.44
1.58×10^2	2.20	29.61	29.44	29.43	29.49	0.10	0.34
3.16×10^0	0.50	N/A	30.52	30.56	30.54	0.03	0.09
Repeat 3							
7.91×10^5	5.90	20.37	20.22	20.82	20.47	0.31	1.53
1.58×10^5	5.20	23.47	22.34	23.37	23.06	0.63	2.71
1.58×10^4	4.20	27.57	25.8	26.43	26.60	0.90	3.37
1.58×10^2	2.20	31.94	31.71	31.64	31.76	0.16	0.49
3.16×10^0	0.50	33.05	32.17	32.31	32.51	0.47	1.45
Total							
7.91×10^5	5.90		19.55			1.09	5.59
1.58×10^5	5.20		21.94			1.17	5.31
1.58×10^4	4.20		25.24			1.71	6.77
1.58×10^2	2.20		30.67			0.99	3.23
3.16×10^0	0.50		31.23			1.11	3.55

Supplementary Table S7.- (Cont)

		Assay I: RNA extraction B					
Viral titer		RT-qPCR results (data in Ct)					
TCID50/gr	Log	Repl 1	Repl 2	Repl 3	Avrg	StDev	CV
			Repeat 1				
7.91×10^5	5.90	18.26	20.1	18.23	18.86	1.07	5.68
1.58×10^5	5.20	20.79	20.82	20.89	20.83	0.05	0.25
1.58×10^4	4.20	24.75	23.45	23.53	23.91	0.73	3.05
1.58×10^2	2.20	29.76	29.55	29.91	29.74	0.18	0.61
3.16×10^0	0.50	30.46	30.9	31.26	30.87	0.40	1.30
			Repeat 2				
7.91×10^5	5.90	18.56	18.62	18.41	18.53	0.11	0.58
1.58×10^5	5.20	20.37	20.37	20.29	20.34	0.05	0.23
1.58×10^4	4.20	23.77	23.61	23.45	23.61	0.16	0.68
1.58×10^2	2.20	30.04	29.82	29.46	29.77	0.29	0.98
3.16×10^0	0.50	29.89	25.46	25.92	27.09	2.44	8.99
			Repeat 3				
7.91×10^5	5.90	20.46	20.53	20.35	20.45	0.09	0.44
1.58×10^5	5.20	21.77	21.98	21.78	21.84	0.12	0.54
1.58×10^4	4.20	26.15	26.08	25.69	25.97	0.25	0.95
1.58×10^2	2.20	31.57	31.57	31.18	31.44	0.23	0.72
3.16×10^0	0.50	32.6	32.76	32.42	32.59	0.17	0.52
			Total				
7.91×10^5	5.90		19.28			1.04	5.39
1.58×10^5	5.20		21.01			0.67	3.17
1.58×10^4	4.20		24.50			1.18	4.82
1.58×10^2	2.20		30.32			0.87	2.86
3.16×10^0	0.50		30.19			2.73	9.06

Supplementary Table S7.- (Cont)

		Assay I: RNA extraction C					
Viral titer		RT-qPCR results (data in Ct)					
TCID50/gr	Log	Repl 1	Repl 2	Repl 3	Avrg	StDev	CV
			Repeat 1[1]				
7.91×10⁵	5.90	18.99	20.01	19.65	19.55	0.52	2.65
1.58×10⁵	5.20	20.16	21.62	21.87	21.22	0.92	4.35
1.58×10⁴	4.20	24.4	24.73	25.57	24.90	0.60	2.42
1.58×10²	2.20	29.82	30.06	30.74	30.21	0.48	1.58
3.16×10⁰	0.50	30.16	31.03	30.89	30.69	0.47	1.52
			Repeat 2				
7.91×10⁵	5.90	17.92	18.38	18.93	18.41	0.51	2.75
1.58×10⁵	5.20	20.43	20.71	21.02	20.72	0.30	1.42
1.58×10⁴	4.20	22.89	23.29	23.66	23.28	0.39	1.65
1.58×10²	2.20	29.13	29.66	29.53	29.44	0.28	0.94
3.16×10⁰	0.50	28.11	29.57	30.49	29.39	1.20	4.08
			Repeat 3				
7.91×10⁵	5.90	20.08	20.58	20.94	20.53	0.43	2.10
1.58×10⁵	5.20	22.14	22.76	22.94	22.61	0.42	1.86
1.58×10⁴	4.20	24.98	25.29	26.14	25.47	0.60	2.36
1.58×10²	2.20	30.55	31.78	31.93	31.42	0.76	2.41
3.16×10⁰	0.50	31.74	32.69	33.05	32.49	0.68	2.08
			Total				
7.91×10⁵	5.90		19.50			1.01	5.19
1.58×10⁵	5.20		21.52			1.00	4.65
1.58×10⁴	4.20		24.55			1.09	4.44
1.58×10²	2.20		30.36			0.98	3.24
3.16×10⁰	0.50		30.86			1.53	4.97

Supplementary Table S7.- (Cont)

Assay I: Summary of results				
Viral titer		qPCR results (data in Ct)		
$TCID_{50}$/gr	Log	Avrg	StDev	CV
Extraction A				
7.91×10^5	5.90	19.48	0.81	4.18
1.58×10^5	5.20	21.29	0.83	3.91
1.58×10^4	4.20	24.95	1.06	4.23
1.58×10^2	2.20	30.24	0.52	1.71
3.16×10^0	0.50	30.65	0.40	1.32
Extraction B				
7.91×10^5	5.90	18.37	0.31	1.67
1.58×10^5	5.20	20.67	0.47	2.28
1.58×10^4	4.20	23.32	0.32	1.35
1.58×10^2	2.20	29.57	0.26	0.88
3.16×10^0	0.50	28.82	2.09	7.26
Extraction C				
7.91×10^5	5.90	20.48	0.27	1.33
1.58×10^5	5.20	22.51	0.66	2.91
1.58×10^4	4.20	26.01	0.74	2.84
1.58×10^2	2.20	31.54	0.44	1.38
3.16×10^0	0.50	32.53	0.42	1.30
Averaged from the 3 extractions				
7.91×10^5	5.90	19.42	1.04	5.38
1.58×10^5	5.20	21.48	1.04	4.84
1.58×10^4	4.20	24.87	1.48	5.93
1.58×10^2	2.20	30.50	0.92	3.02
3.16×10^0	0.50	30.68	2.14	6.96

Assay II: No replicas and no repeats		
Viral titer		qPCR results (data in Ct)
$TCID_{50}$/gr	Log	Ct
2.51×10^7	7.4	14.76
1.26×10^7	7.0	16.02
2.51×10^6	6.4	17.75
2.51×10^5	5.4	20.55
2.51×10^4	4.4	22.99
1.26×10^3	3.1	25.48
6.31×10^1	1.8	26.10
3.16×10^0	0.5	27.02

Assay I: RNA extracted (3 independent extractions: A to C[1]) from fish tissues infected *in vitro* with a known viral titer ([2]) was subjected to RT-qPCR amplification ([3]). Data for each repeat are the average from 3 replicas ([4]). Assay II: A new infected tissue was tested with no replicas and repeats.

Supplementary Table S8.- Statistical differences between viral titers in experimentally infected fish tissues deduced from the Ct values observed by RT-qPCR and the different standard curves

A/ Strain: RG/SJ; Species: Senegalese sole (*Solea senegalensis*)

			**Tissue**							
**Sample[1]**	**Ct[2]**	**Titer[3]**	**Lin. Rgr.[4]**	**P2 Rgr[5]**	**CV-RNA[6]**	**Purif virus[7]**	**pDNA[8]**	**ivRNA[9]**	**CC[10]**	**nPCR[11]**
1	18.22	6.90	6.59	6.09	11.24	7.88	5.20	5.56	+	+
2	18.28	6.88	6.56	6.08	11.21	7.87	5.18	5.54	+	+
3	20.25	6.20	5.74	5.66	10.54	7.38	4.64	4.94	+	+
4	22.07	5.57	4.98	5.15	9.91	6.93	4.14	4.40	+	+
5	22.12	5.56	4.95	5.14	9.89	6.92	4.13	4.38	+	+
6	23.27	5.16	4.47	4.76	9.50	6.63	3.81	4.04	+	+
7	23.34	5.14	4.44	4.74	9.47	6.61	3.79	4.01	+	+
8	23.49	5.09	4.38	4.68	9.42	6.58	3.75	3.97	+	+
9	24.02	4.90	4.16	4.49	9.24	6.45	3.61	3.81	+	+
10	25.28	4.47	3.63	4.00	8.81	6.13	3.26	3.43	-	+
11	25.29	4.47	3.63	4.00	8.80	6.13	3.26	3.43	+	+
12	25.44	4.41	3.56	3.93	8.75	6.09	3.21	3.38	+	+
13	29.12	3.15	2.02	2.17	7.49	5.18	2.20	2.27	+	+
14	30.60	2.64	1.40	1.34	6.98	4.81	1.79	1.83	-	+
15	30.61	2.64	1.39	1.33	6.97	4.81	1.79	1.83	+	+
16	31.31	2.40	1.10	0.91	6.73	4.64	1.60	1.62	-	+
17	31.77	2.24	0.91	0.63	6.58	4.53	1.47	1.48	-	+
18	31.85	2.21	0.87	0.58	6.55	4.51	1.45	1.45	-	+
19	32.83	1.87	0.46	-0.06	6.21	4.26	1.18	1.16	-	+
20	33.21	1.74	0.30	-0.31	6.08	4.17	1.08	1.04	-	-
21	33.61	1.60	0.13	-0.59	5.94	4.07	0.96	0.92	-	-
22	35.11	1.09	-0.49	-1.65	5.43	3.70	0.55	0.47	-	-
C-	-	N/A	N/A	N/A	N/A	N/A	N/A	N/A	-	-
C-	-	N/A	N/A	N/A	N/A	N/A	N/A	N/A	-	-
C-	-	N/A	N/A	N/A	N/A	N/A	N/A	N/A	-	-
C-	-	N/A	N/A	N/A	N/A	N/A	N/A	N/A	-	-

<u>Comparison by T student</u> <u>(P values are shown)</u>

	Titer	Lin Rgr	P2-Rgr	CV-RNA	Purif. V.	pDNA	ivRNA
Titer	-	0.1214	0.1108	<0.0001	0.0004	0.0308	0.065
Tiss-Lin Rgr	-	-	0.8912	<0.0001	<0.0001	0.8015	0.9863
Tiss-P2 Rgr	-	-	-	<0.0001	<0.0001	0.9394	0.8907
CV-RNA	-	-	-	-	<0.0001	<0.0001	<0.0001
Purif. Virus	-	-	-	-	-	<0.0001	<0.0001
pDNA	-	-	-	-	-	-	0.7738
ivRNA	-	-	-	-	-	-	-

B/ Strain: RG/RG; Species: Sea bass (Dicentrarchus labrax)

			**Tissue**							
**Sample**[1]	**Ct**[2]	**Titer**[3]	**Lin. Rgr.**[4]	**P2 Rgr**[5]	**CV-RNA**[6]	**Purif v.**[7]	**pDNA**[8]	**ivRNA**[9]	**CC**[10]	**nPC**
1	4.35	11.66	12.41	5.41	16.00	11.32	9.01	9.73	+	+
2	7.10	10.72	11.25	6.05	15.06	10.64	8.26	8.90	+	+
3	9.53	9.88	10.23	6.41	14.22	10.03	7.59	8.17	+	+
4	9.98	9.73	10.04	6.45	14.07	9.92	7.47	8.03	+	+
5	10.18	9.66	9.96	6.47	14.00	9.87	7.41	7.97	+	+
6	12.23	8.96	9.10	6.57	13.29	9.37	6.85	7.36	+	+
7	14.55	8.16	8.13	6.53	12.49	8.79	6.21	6.66	+	+
8	14.67	8.12	8.08	6.52	12.45	8.76	6.17	6.62	+	+
9	14.73	8.10	8.05	6.52	12.43	8.75	6.16	6.61	+	+
10	15.40	7.87	7.77	6.47	12.20	8.58	5.98	6.41	+	+
11	16.10	7.63	7.48	6.40	11.96	8.41	5.78	6.19	+	+
12	16.32	7.55	7.39	6.37	11.89	8.35	5.72	6.13	+	+
13	16.69	7.42	7.23	6.33	11.76	8.26	5.62	6.02	+	+
14	16.97	7.33	7.11	6.29	11.66	8.19	5.54	5.93	+	+
15	17.36	7.19	6.95	6.23	11.53	8.10	5.44	5.82	+	+
16	17.97	6.98	6.70	6.13	11.32	7.94	5.27	5.63	+	+
17	18.16	6.92	6.62	6.10	11.26	7.90	5.22	5.58	+	+
18	18.38	6.84	6.52	6.06	11.18	7.84	5.16	5.51	+	+
19	18.92	6.66	6.30	5.96	10.99	7.71	5.01	5.34	+	+
20	21.67	5.71	5.14	5.27	10.05	7.03	4.25	4.52	+	+
21	22.11	5.56	4.96	5.14	9.90	6.92	4.13	4.39	+	+
22	25.35	4.45	3.60	3.97	8.78	6.12	3.24	3.41	+	+
23	27.70	3.64	2.62	2.91	7.98	5.53	2.59	2.70	+	+
24	28.58	3.33	2.25	2.46	7.67	5.32	2.35	2.44	+	+
25	29.10	3.16	2.03	2.18	7.49	5.19	2.21	2.28	-	+
26	29.33	3.08	1.93	2.06	7.42	5.13	2.14	2.21	+	+

27	30.04	2.83	1.63	1.66	7.17	4.95	1.95	2.00	-	+
28	32.03	2.15	0.80	0.46	6.48	4.46	1.40	1.40	-	-
29	34.33	1.36	-0.17	-1.09	5.70	3.89	0.77	0.71	-	-
C-	-	N/A	N/A	N/A	N/A	N/A	N/A	N/A	-	-
C-	-	N/A	N/A	N/A	N/A	N/A	N/A	N/A	-	-
C-	-	N/A	N/A	N/A	N/A	N/A	N/A	N/A	-	-
C-	-	N/A	N/A	N/A	N/A	N/A	N/A	N/A	-	-
C-	-	N/A	N/A	N/A	N/A	N/A	N/A	N/A	-	-

Comparison by T student (P values are shown)

	Titer	Lin Rgr	P2 Rgr	CV-RNA	Purif. V.	pDNA	ivRNA
Titer	-	0.6426	0.0116[12]	<0.0001	0.0915	0.0126	0.0532
Tiss-Lin Rgr	-	-	0.0787	<0.0001	0.0495	0.0834	0.2117
Tiss-P2 Rgr	-	-	-	<0.0001	<0.0001	0.9704	0.5479
CV-RNA	-	-	-	-	<0.0001	<0.0001	<0.0001
Purif. Virus	-	-	-	-	-	<0.0001	0.0001
pDNA	-	-	-	-	-	-	0.5713
ivRNA	-	-	-	-	-	-	-

1, Sample no.; 2, Ct value observed by RT-qPCR; 3, Viral titer [$Log_{10}(TCID_{50}/ml)$] deduced from the line y = -0.3438x + 13.161 extracted from the viral titer standard curve (Table 2); 4, Viral titer [$Log_{10}(TCID_{50}/ml)$] deduced from the line y = -0.4194x + 14.232 extracted from the fish tissues linear standard curve (Table 6); 5, Viral titer [$Log_{10}(TCID_{50}/ml)$] deduced from the line $y = -0.0165x^2 + 0.4216x+3.8858$ extracted from the fish tissues second degree polynomial standard curve (Table 6); 6, Viral titer [Log_{10} (copies/ml)] deduced from the line y = -0.3438x + 17.498 extracted from the crude virus RNA standard curve (Table 2); 7, Viral titer [Log_{10} ($TCID_{50}$/ml)] deduced from the line y =- 0,2477x + 12,395 extracted from the purified virus standard curve (Table 3); 8, Viral titer [Log_{10} (copies/ml)] deduced from the line y = -0.2751x + 10.212 extracted from the pDNA standard curve (Table 4); 9, Viral titer [Log_{10} (copies/ml)] deduced from the line y = -0.3010x + 11.040 extracted from the ivRNA standard curve (Table 5); 10, isolation in cell culture (+, isolated); 11, result obtained with nPCR (+, detected); 12, No significative differences (P=0.0654) observed in the Ct range 11-35.

Supplementary Figure S1

A/ SJNNV type strain

a-Standard curves for each replica and repeat

Repeat 1 | Repeat 2 | Repeat 3

Replica 1

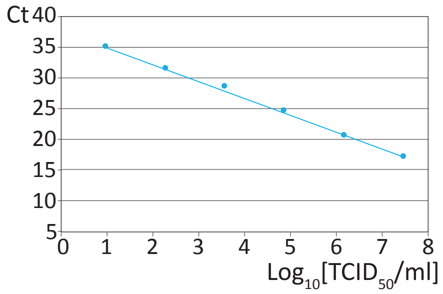

$y = -2.8026x + 38.161$
$R^2 = 0.9985$ / E=127.4

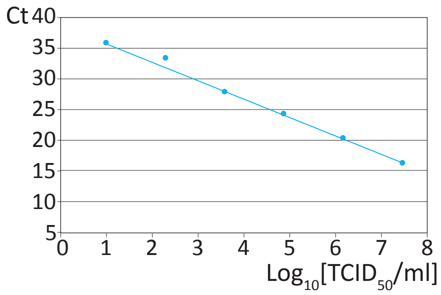

$y = -3.0677x + 39.145$
$R^2 = 0.9948$ / E=111.8

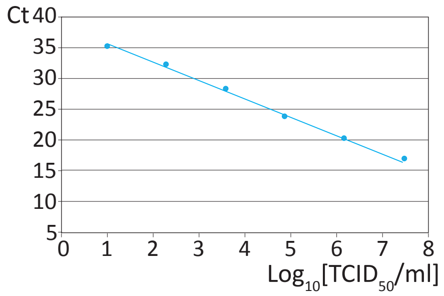

$y = -2.9346x + 38.426$
$R^2 = 0.9980$ / E=119.2

Replica 2

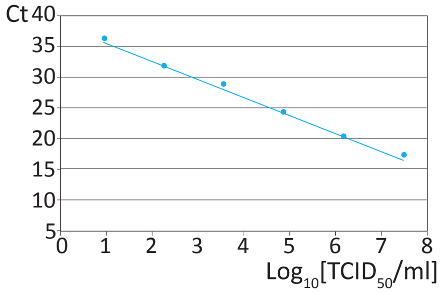

$y = -2.9673x + 38.943$
$R^2 = 0.9982$ / E=117.3

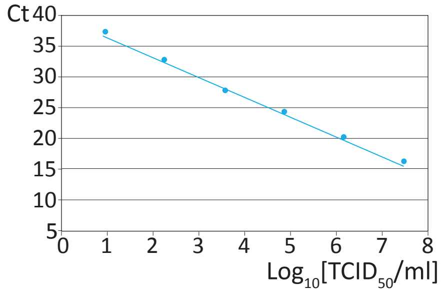

$y = -3.1777x + 39.787$
$R^2 = 0.9966$ / E=106.4

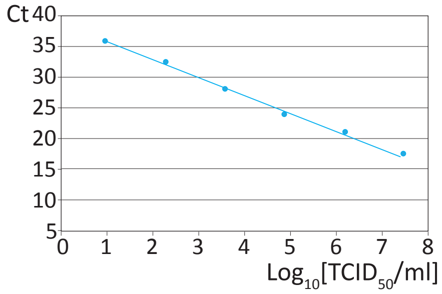

$y = -2.8891x + 38.315$
$R^2 = 0.9969$ / E=121.9

Replica 3

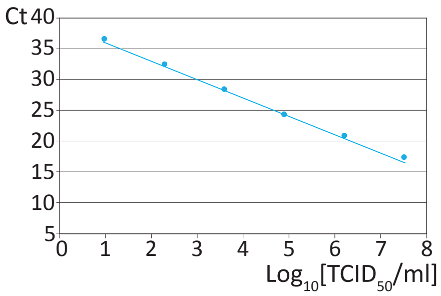

$y = -2.953x + 38.855$
$R^2 = 0.9993$ / E=118.1

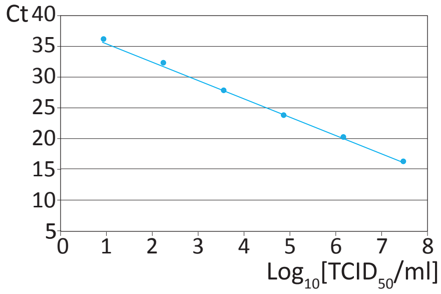

$y = -3.0429x + 38.958$
$R^2 = 0.9993$ / E=113.1

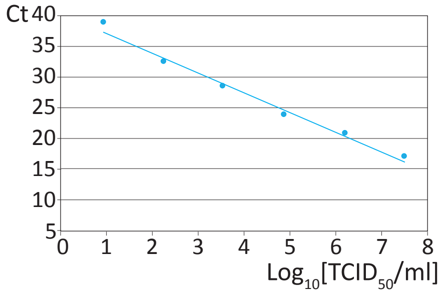

$y = -3.2337x + 40.543$
$R^2 = 0.9816$ / E=103.8

b-Standard curves for each repeat

Repeat 1

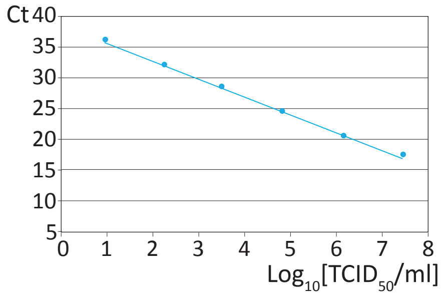

$y = -2.9077x + 38.653$
$R^2 = 0.9994$ / E=120.8

Repeat 2

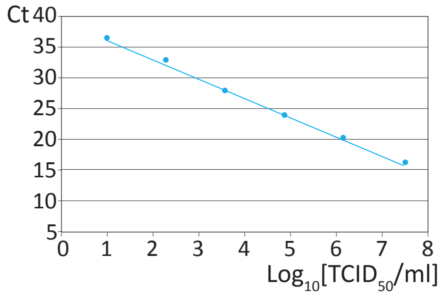

$y = -3.0961x + 39.296$
$R^2 = 0.9982$ / E=110.4

Repeat 3

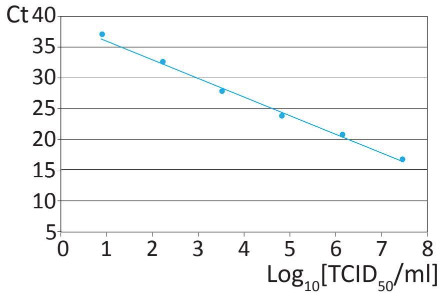

$y = -3.0191x + 39.095$
$R^2 = 0.9963$ / E=114.4

c-SJNNV viral titer general standard curve

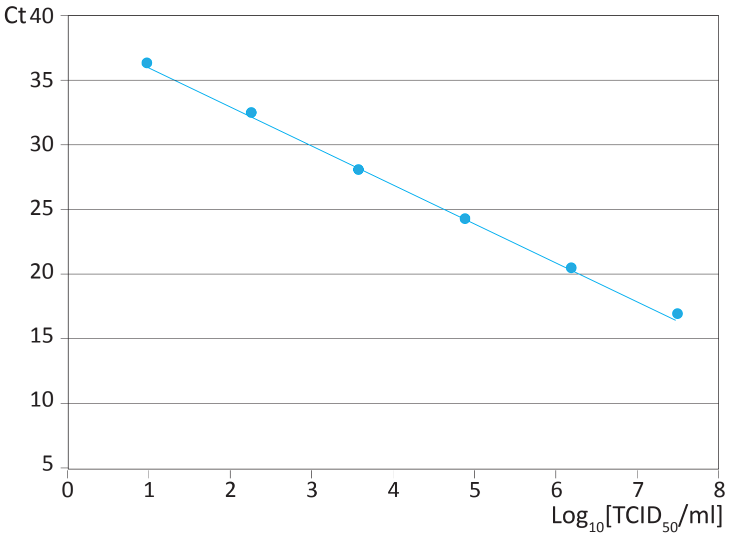

y = -3.0076x + 39.015
R^2 = 0.9990 / E=115.0

Supplementary Figure S1.- Cont.

B/ RGNNV type strain

a-Standard curves for each replica and repeat

Repeat 1 | Repeat 2 | Repeat 3

Replica 1

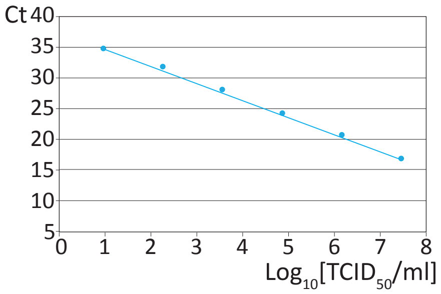

y = -2.8042x + 37.702
R^2 = 0.9980 / E=127.3

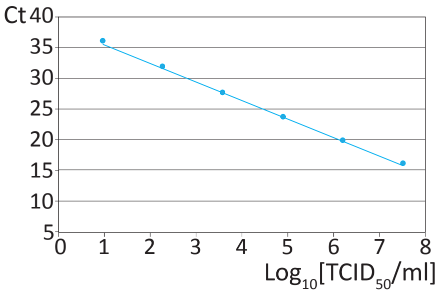

y = -3.0229x + 38.491
R^2 = 0.9990 / E=114.2

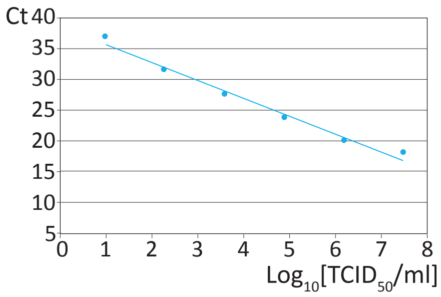

y = -2.9306x + 38.649
R^2 = 0.9689 / E=119.4

Replica 2

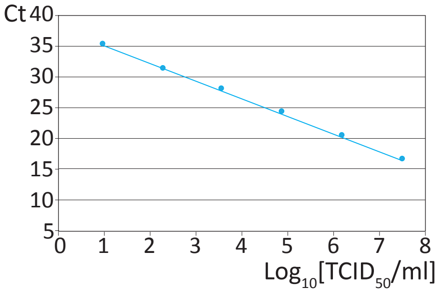

y = -2.9028x + 38.273
R^2 = 0.9993 / E=121.1

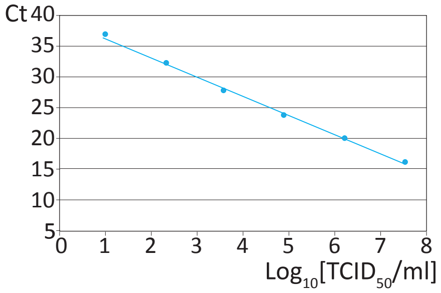

y = -3.1562x + 39.259
R^2 = 0.9959 / E=107.4

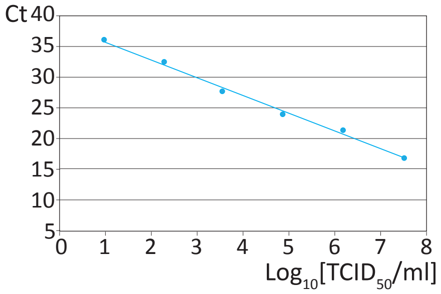

y = -2.881x + 38.389
R^2 = 0.9873 / E=122.4

Replica 3

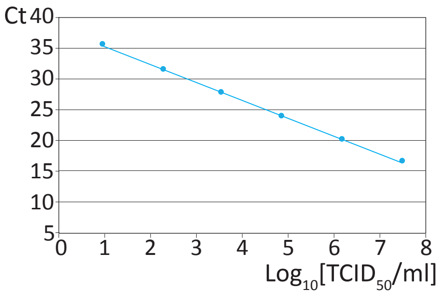

y = -2.9076x + 38.156
R^2 = 0.9988 / E=120.8

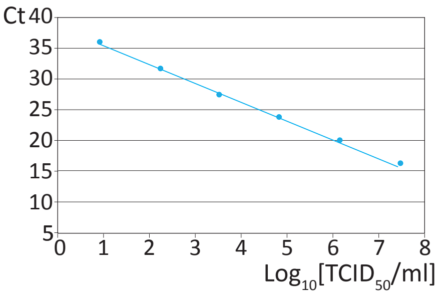

y = -3.0251x + 38.544
R^2 = 0.9973 / E=114.1

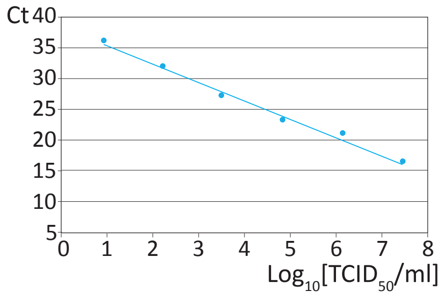

y = -2.9533x + 38.454
R^2 = 0.9918 / E=118.1

b-Standard curves for each repeat

Repeat 1 | Repeat 2 | Repeat 3

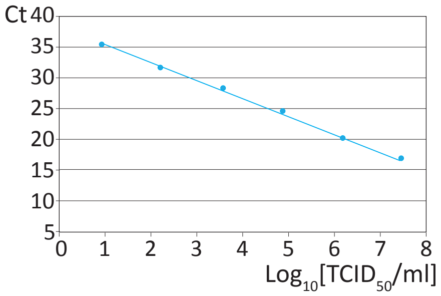

y = -2.8715x + 38.044
R^2 = 0.9998 / E=123.0

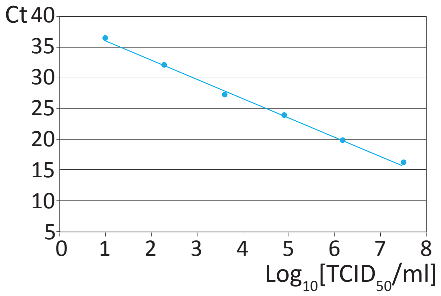

y = -3.068x + 38.765
R^2 = 0.9977 / E=118.8

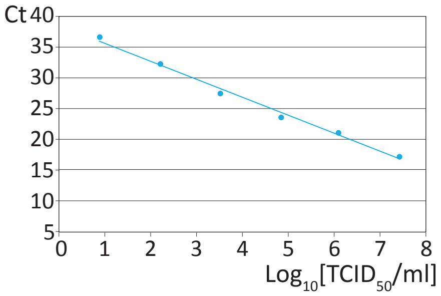

y = -2.9216x + 38.497
R^2 = 0.9896 / E=119.9

c-RGNNV viral titer general standard curve

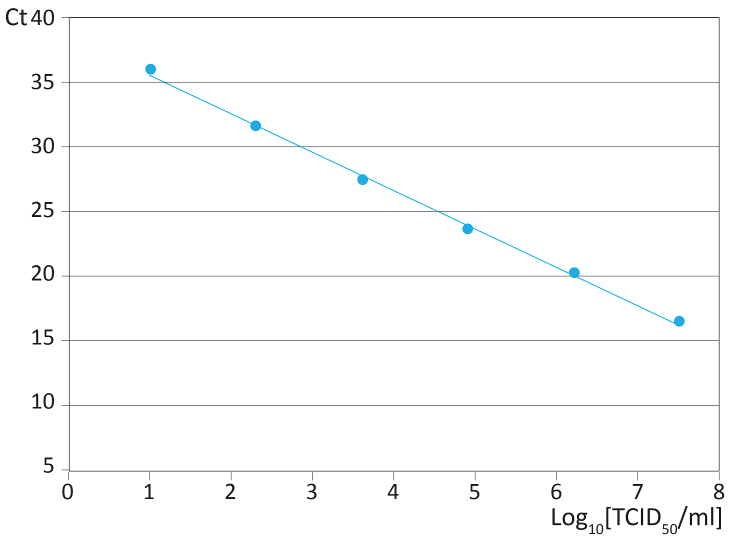

y = -2.9537x + 38.435
R^2 = 0.9976 / E=118.0

Supplementary Figure S1.- Cont.

C/ BFNNV type strain

a-Standard curves for each replica and repeat

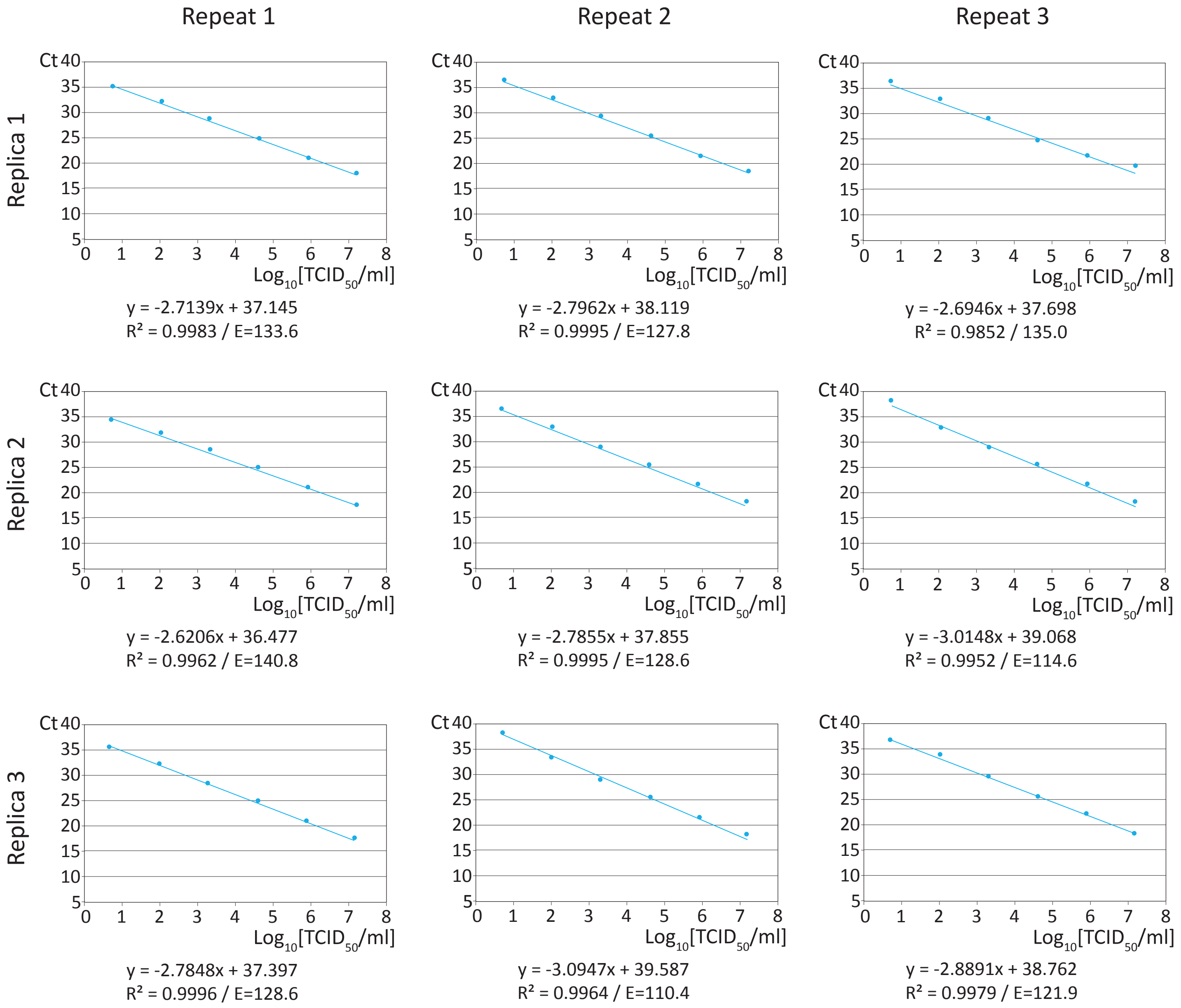

b-Standard curves for each repeat

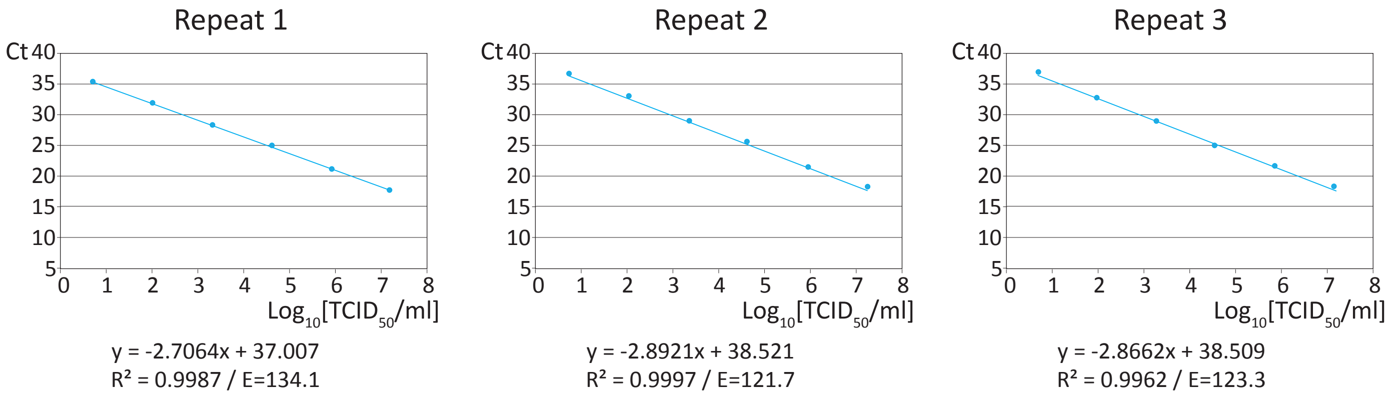

c-BFNNV viral titer general standard curve

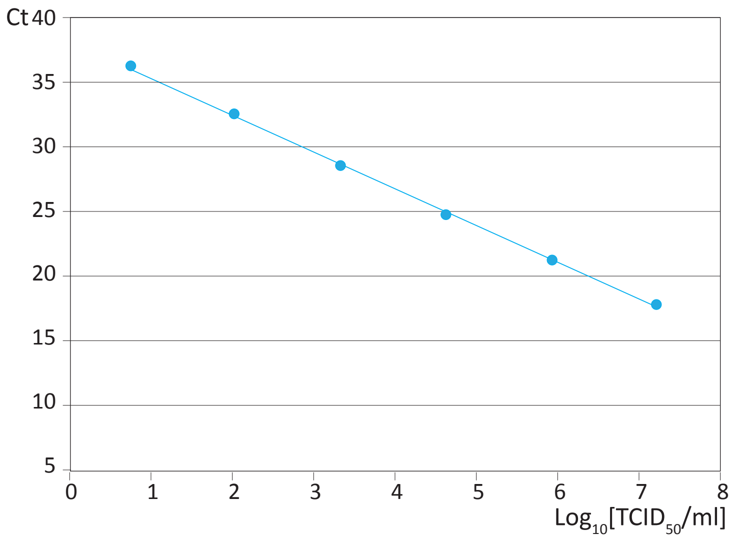

$y = -2.8216x + 38.012$
$R^2 = 0.9993$ / E=126.2

Supplementary Figure S1.- Cont.

D/ TPNNV type strain

a-Standard curves for each replica and repeat

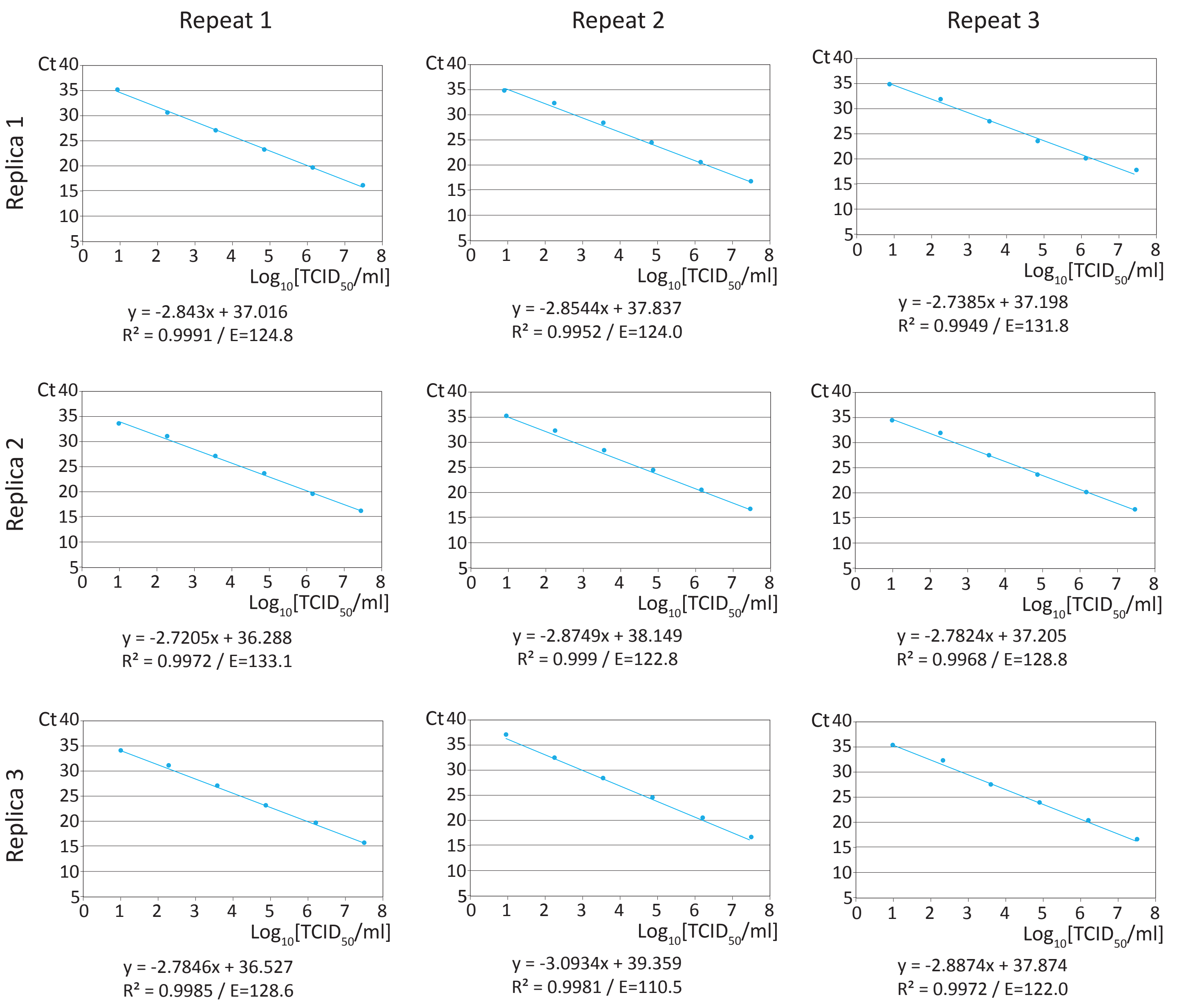

b-Standard curves for each repeat

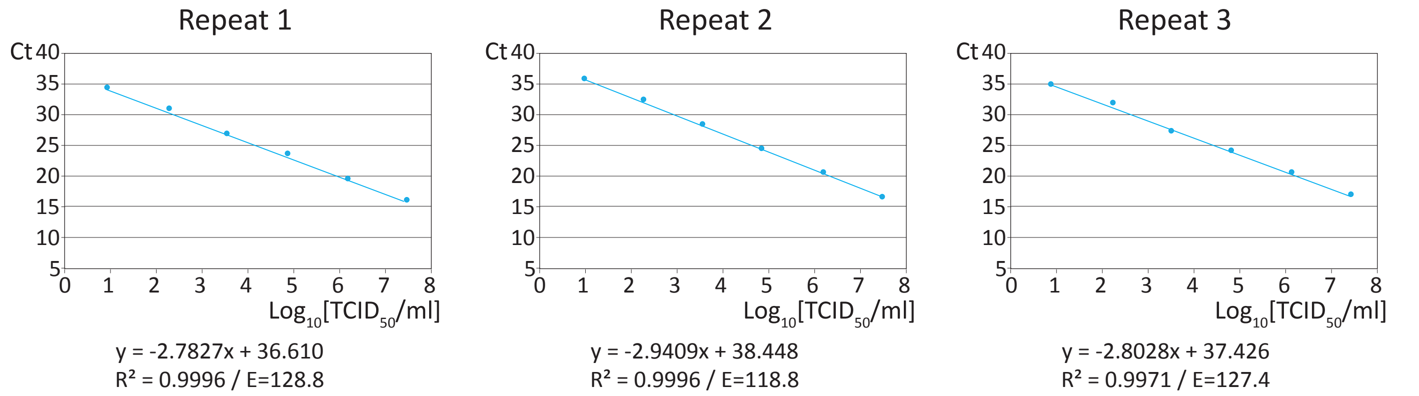

c-TPNNV viral titer general standard curve

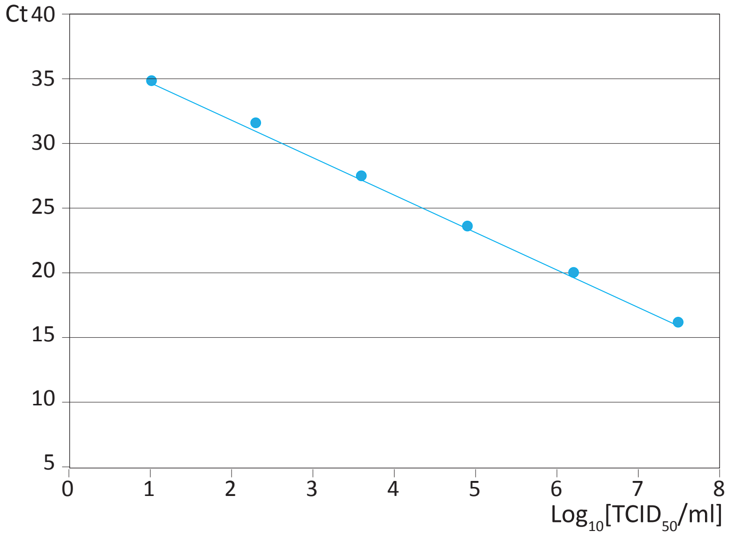

y = -2.8421x + 37.495
R^2 = 0.9992 / E=124.8

Supplementary Figure S1.- Cont.

E/ Standard curved averaged from the 4 strains

a-Standard curves for each replica and repeat

Repeat 1 | Repeat 2 | Repeat 3

Replica 1

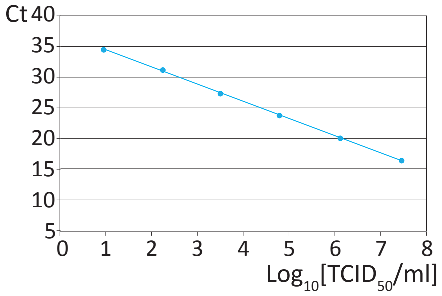

y = -2.7909x + 37.535
R^2 = 0.9994 / E=128.2

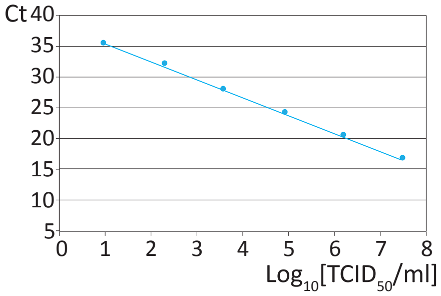

y = -2.9353x + 38.425
R^2 = 0.9992 / E=119.1

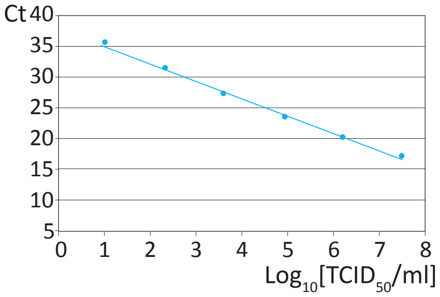

y = -2.8246x + 38.019
R^2 = 0.9924 / E=126.0

Replica 2

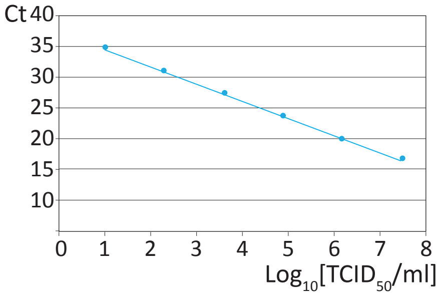

y = -2.8028x + 37.518
R^2 = 0.9996 / E=127.4

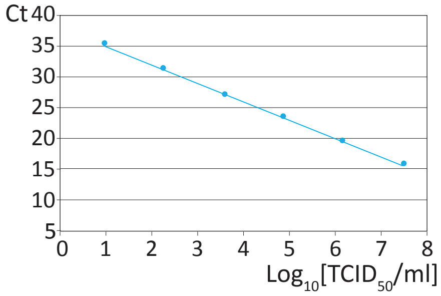

y = -2.9986x + 38.786
R^2 = 0.9995 / E=115.5

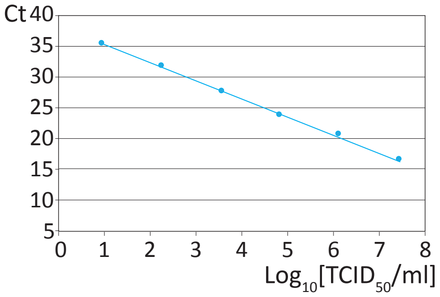

y = -2.8918x + 38.287
R^2 = 0.9975 / E=121.7

Replica 3

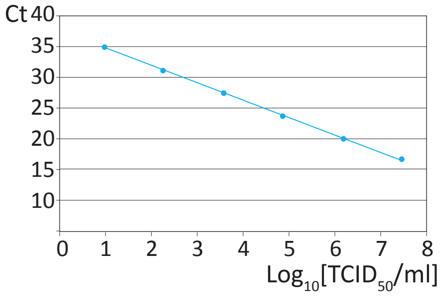

y = -2.8575x + 37.764
R^2 = 0.9997 / E=123.8

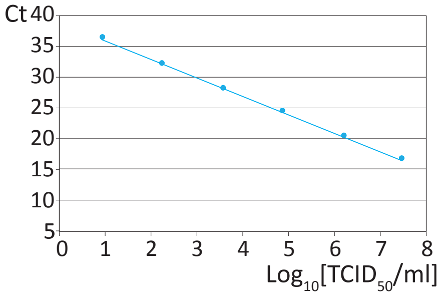

y = -3.064x + 39.151
R^2 = 0.9982 / E=112.0

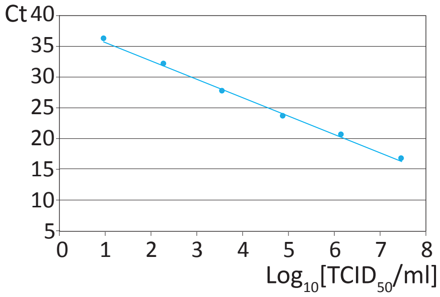

y = -2.9909x + 39.938
R^2 = 0.9959 / E=115.9

b-Standard curves for each repeat

Repeat 1

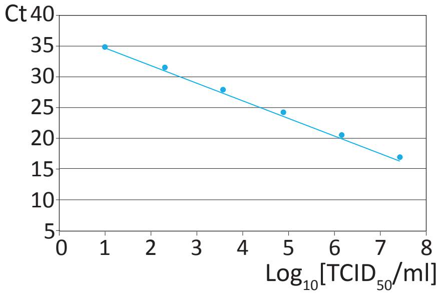

y = -2.8171x + 37.606
R^2 = 0.9997 / E=126.5

Repeat 2

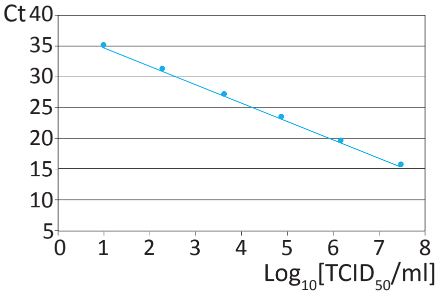

y = -2.9993x + 38.787
R^2 = 0.9995 / E=115.5

Repeat 3

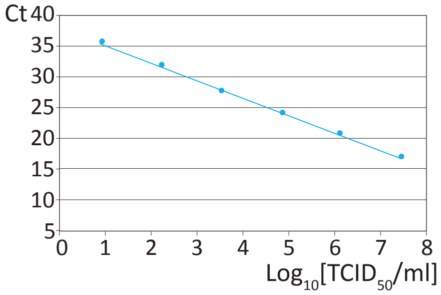

y = -2.9024x + 38.415
R^2 = 0.9963 / E=121.1

c-Viral titer (averaged from the 4 strains) general standard curve

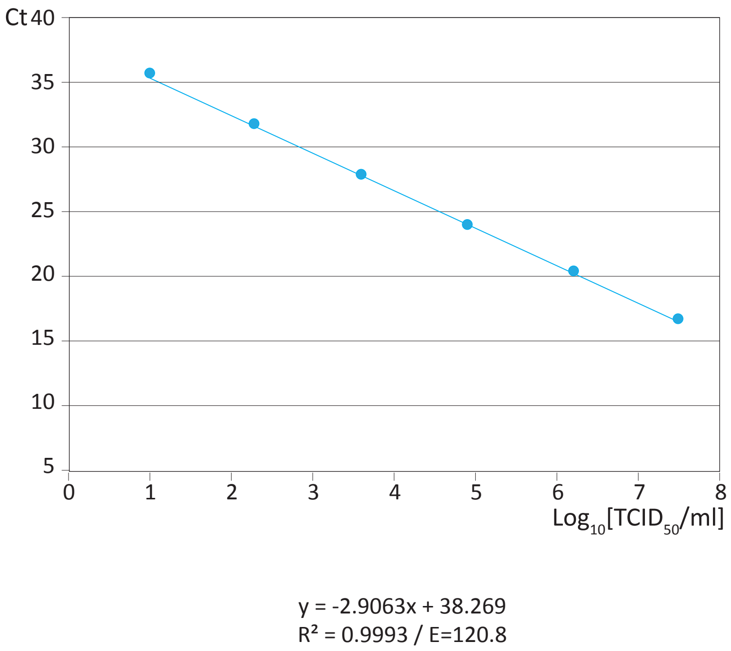

Figure S1.- Standard curves using crude virus titers.- For each curve, the equation and its reliability (given as the coefficient of determination (R^2) and the efficiency (E) of the amplification) is shown. The graphs are organized in 5 sections: sections **A** to **D** with the curves obtained with each type strain, and calibration curves averaging the data from the four strains are shown in section **E**. In each section, the curves are shown in 3 ways: **a**-All the curves obtained with the corresponding viral type in every replica and repeat; **b**- The standard curve for each repeat (averaging the 3 replicas), and **c**- the general standard curve (averaging data from all replicas and repeats).

Supplementary Figure S2

A/ SJNNV type strain

a- Standard curves for each replica and repeat

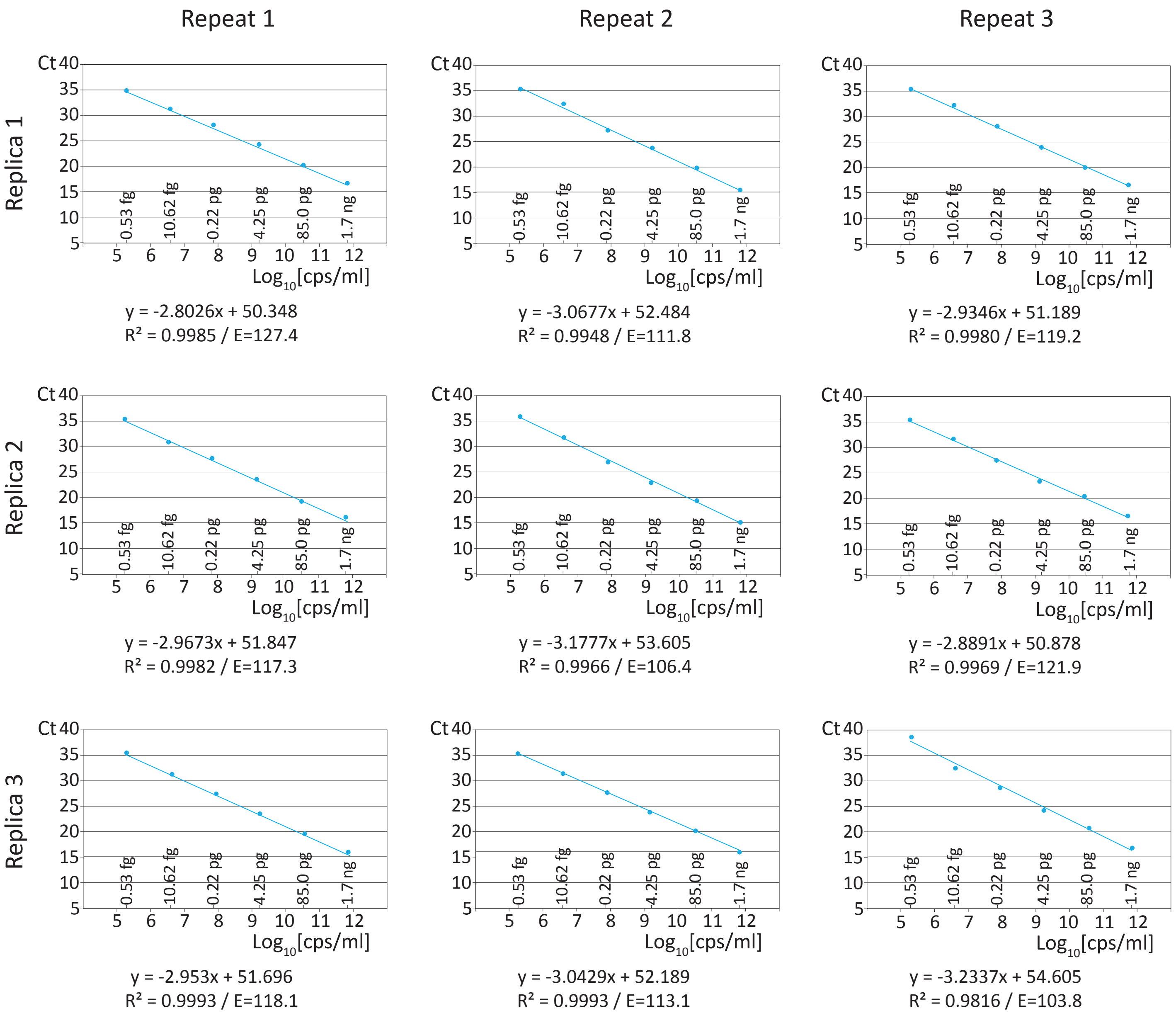

b- Standard curves for each repeat

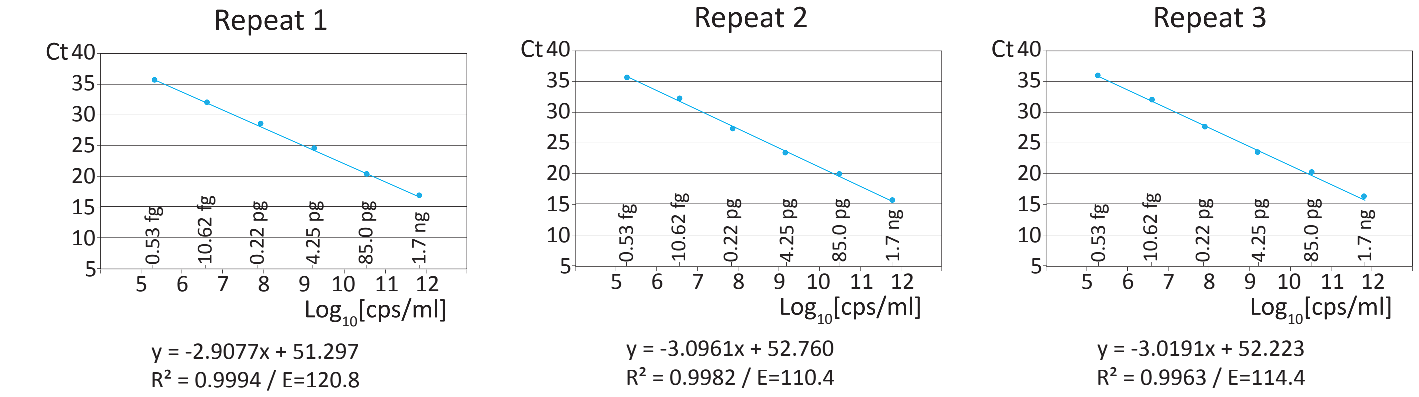

c- SJNNV RNA copies general standard curve

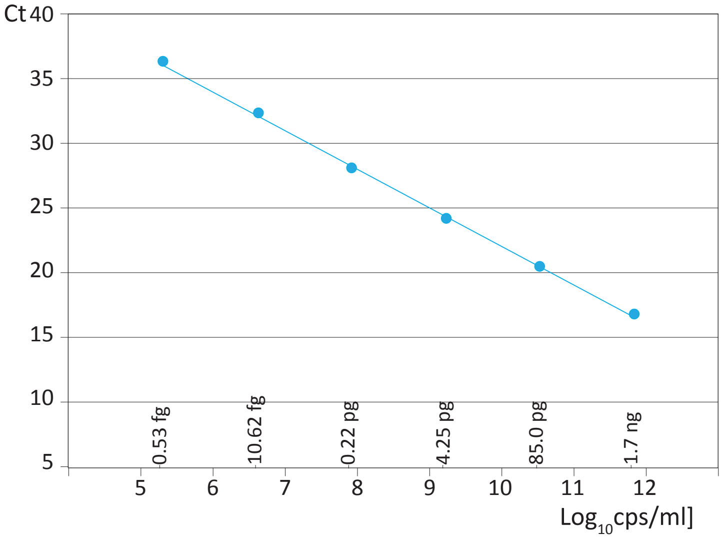

y = -3.0076x + 52.093

R^2 = 0.9990 / E=115.0

Supplementary Figure S2.- Cont.

B/ RGNNV type strain

a- Standard curves for each replica and repeat

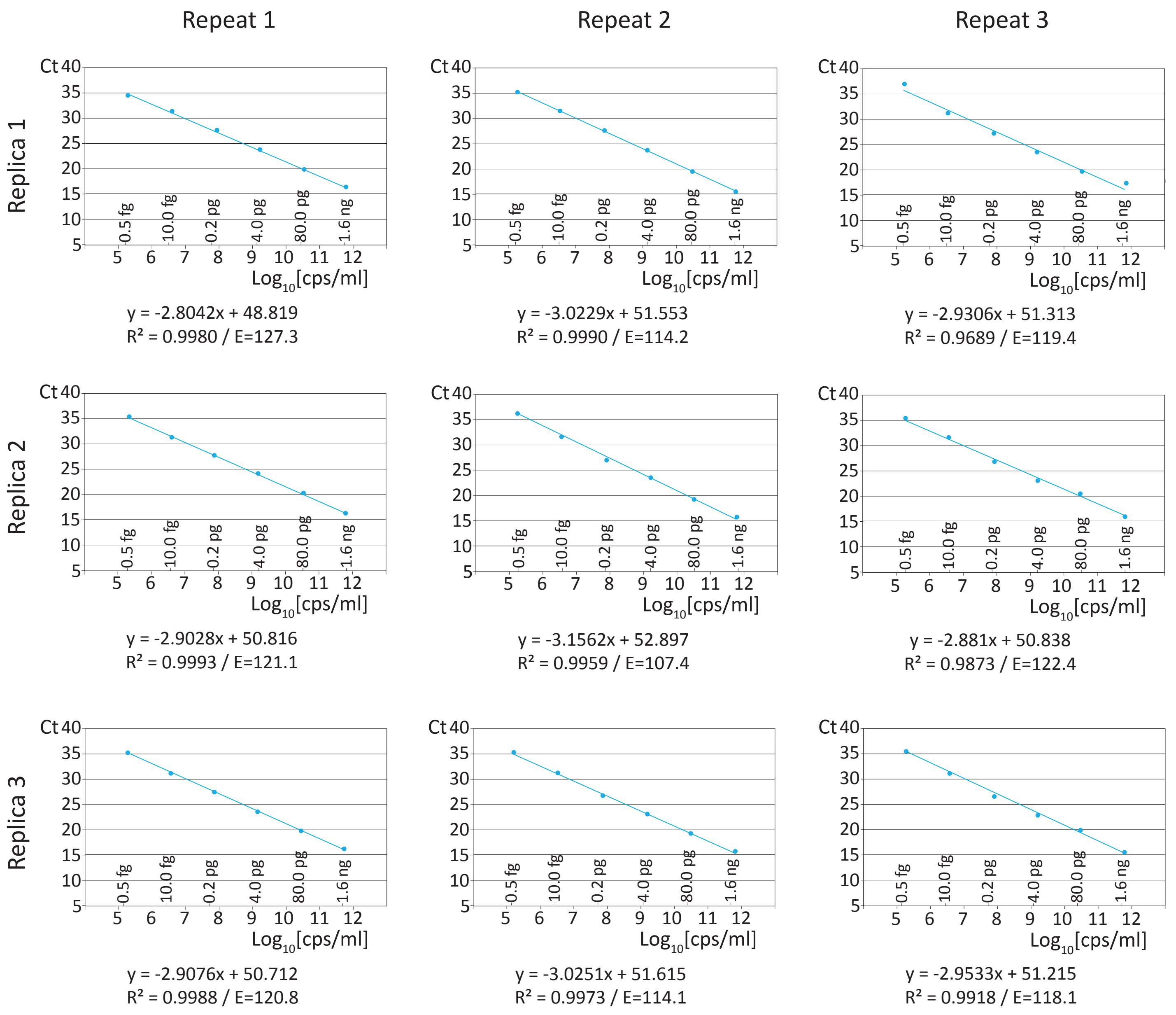

b- Standard curves for each repeat

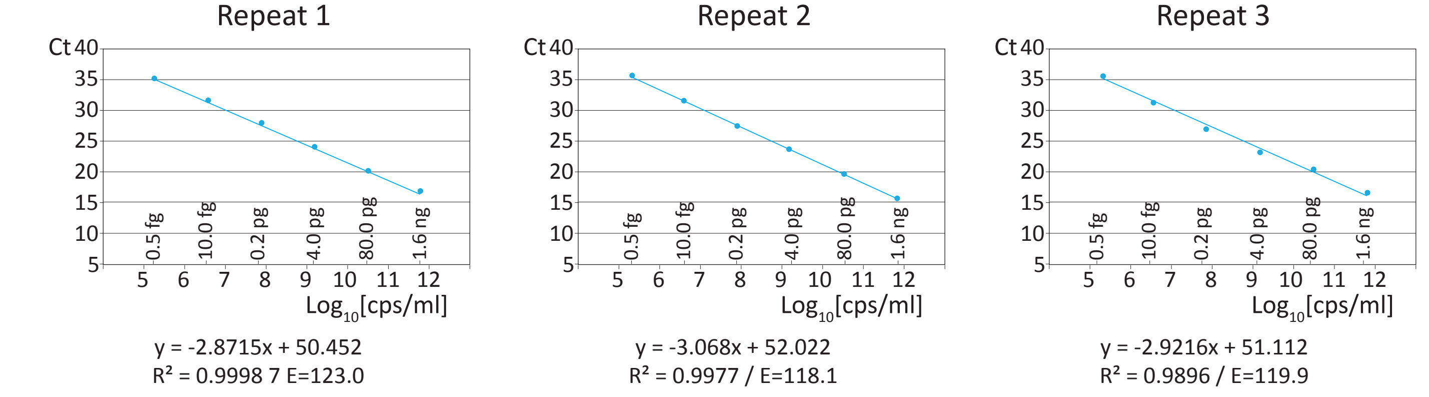

c- RGNNV RNA copies general standard curve

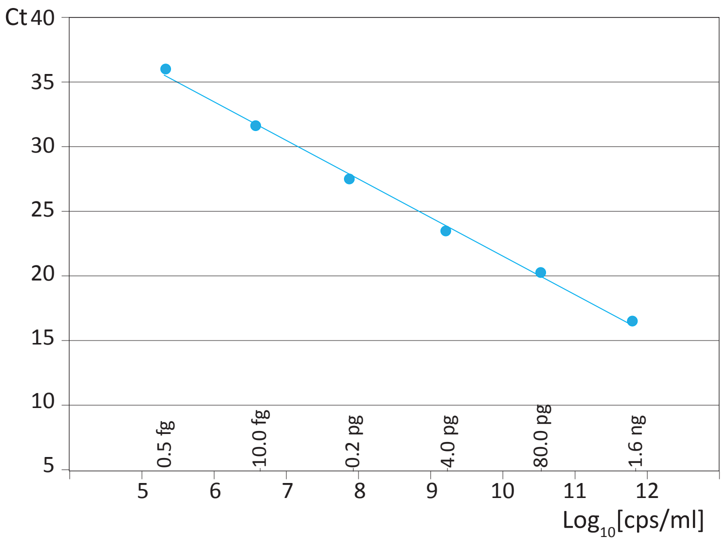

y = -2.9537x + 51.198

R^2 = 0.9976 / E=118.0

Supplementary Figure S2.- Cont.

C/ BFNNV type strain

a- Standard curves for each replica and repeat

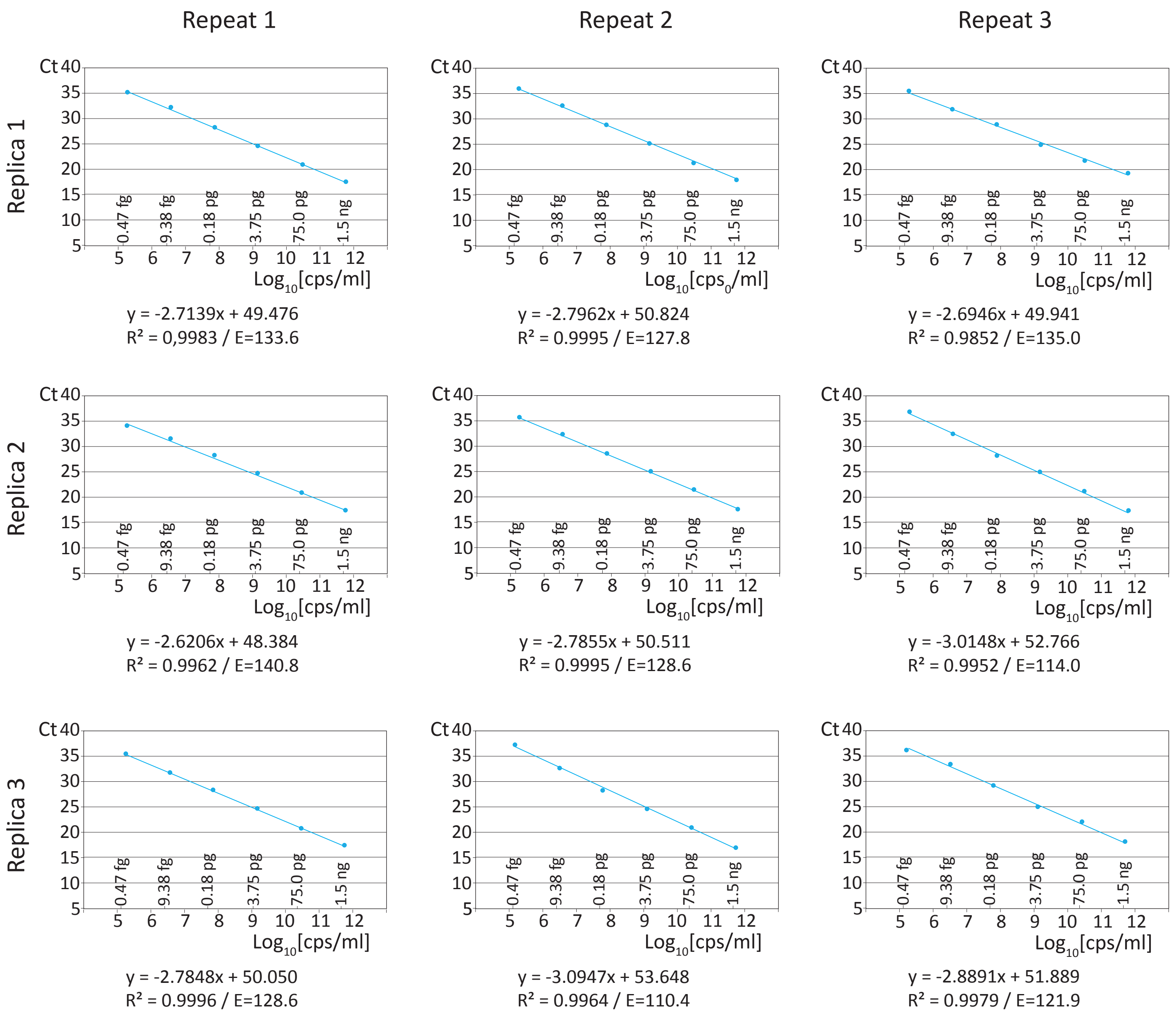

b- Standard curves for each repeat

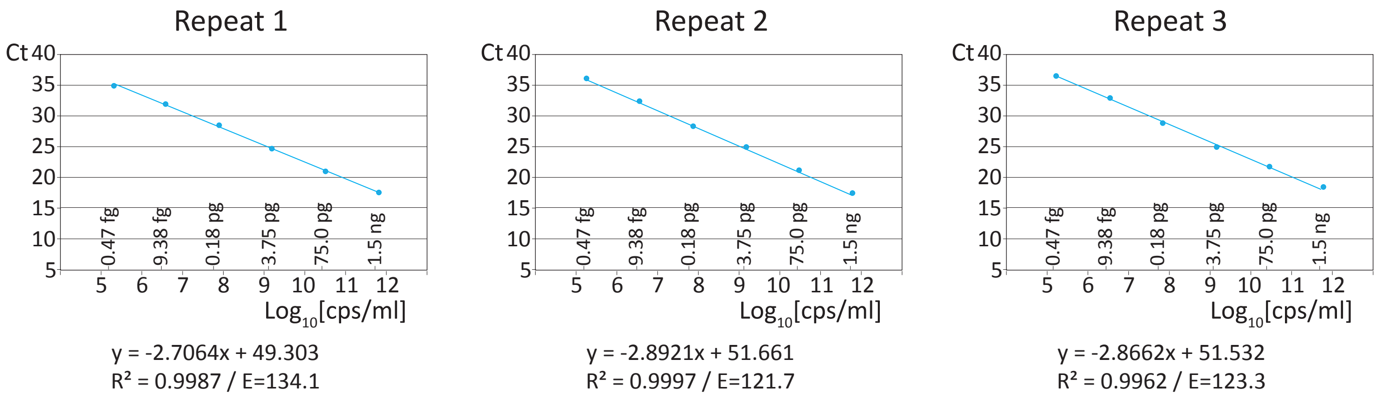

c- BFNNV RNA copies general standard curve

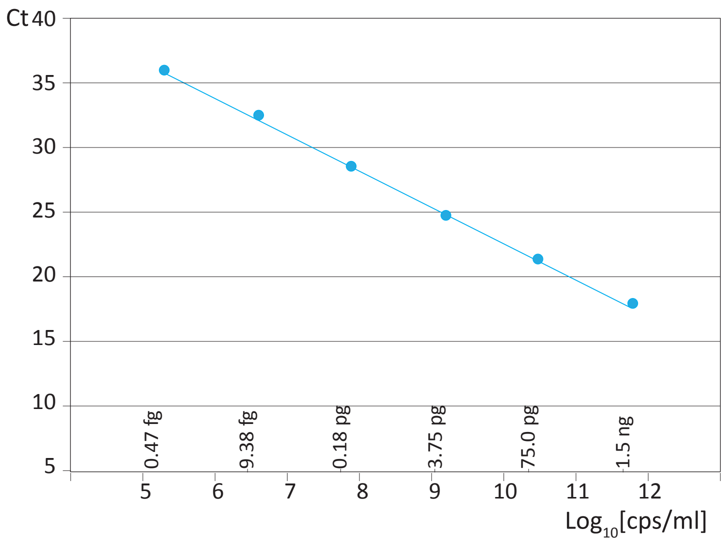

y = -2.8216x + 50.832

R^2 = 0.9993 / E=126.2

Supplementary Figure S2.- Cont.

D/ TPNNV type strain

a- Standard curves for each replica and repeat

Repeat 1 | Repeat 2 | Repeat 3

Replica 1

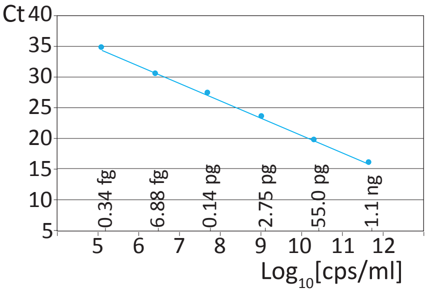

y = -2.843x + 40.310
R^2 = 0.9991 / E=124.8

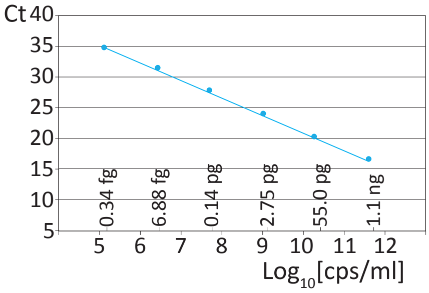

y = -2.8544x + 41.145
R^2 = 0.9952 / E=124.0

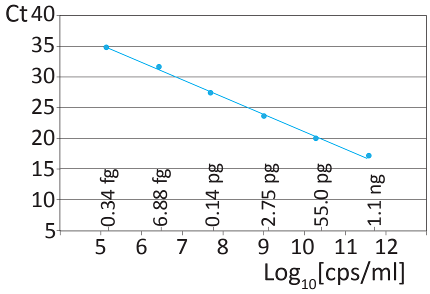

y = -2.7385x + 40.372
R^2 = 0.9949 7 E=131.8

Replica 2

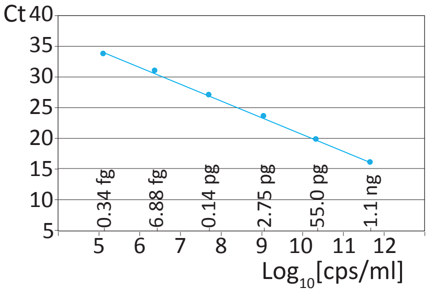

y = -2.7205x + 39.441
R^2 = 0.9972 / E=133.1

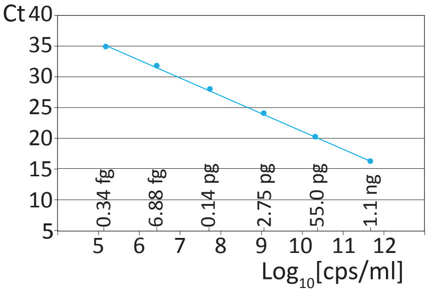

y = -2.8749x + 41.481
R^2 = 0.9990 / E=122.8

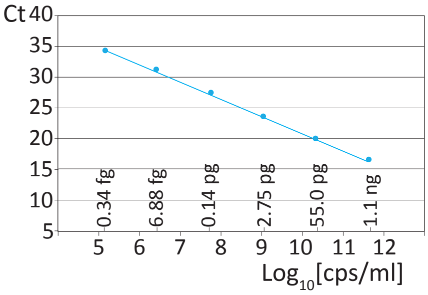

y = -2.7824x + 40.429
R^2 = 0.9968 7 E=128.8

Replica 3

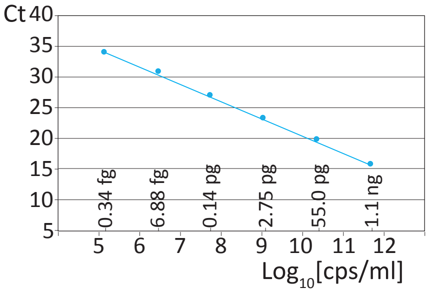

y = -2.7846x + 39.754
R^2 = 0.9985 / E=128.6

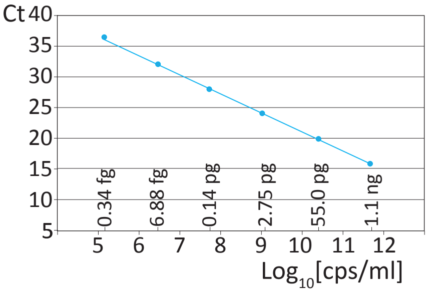

y = -3.0934x + 42.944
R^2 = 0.9981 / E=110.5

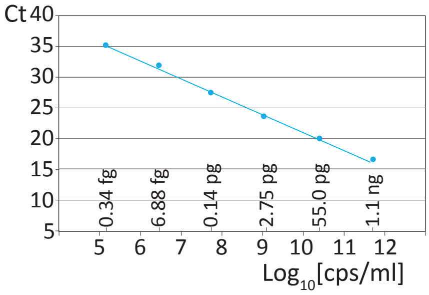

y = -2.8874x + 41.220
R^2 = 0.9972 / E=122.0

b- Standard curves for each repeat

Repeat 1

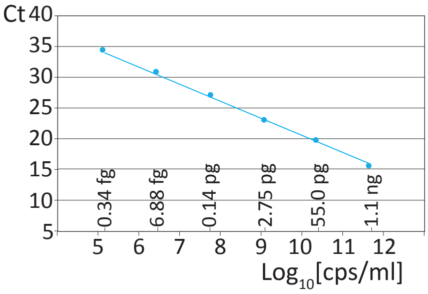

y = -2.7827x + 39.835
R^2 = 0.9996 / E=128.8

Repeat 2

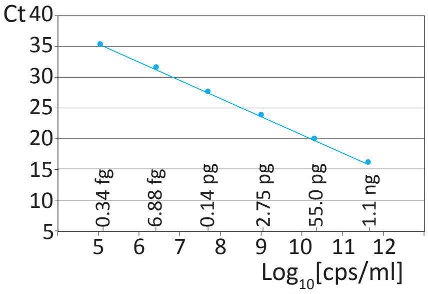

y = -2.9409x + 41.856
R^2 = 0.9996 / E=118.8

Repeat 3

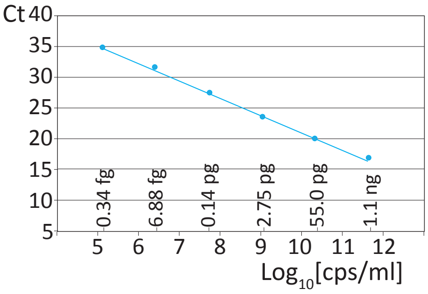

y = -2.8028x + 40.674
R^2 = 0.9971 / E=127.4

c- TPNNV RNA copies general standard curve

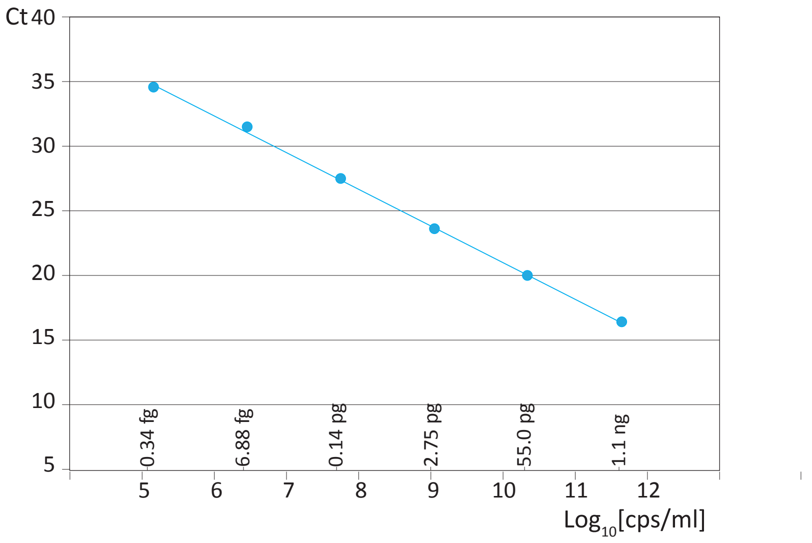

$y = -2.8421x + 40.788$
$R^2 = 0.9992$ / E=124.8

Supplementary Figure S2.- Cont.

E/ Standard curved averaged from the 4 strains

a- Standard curves for each replica and repeat

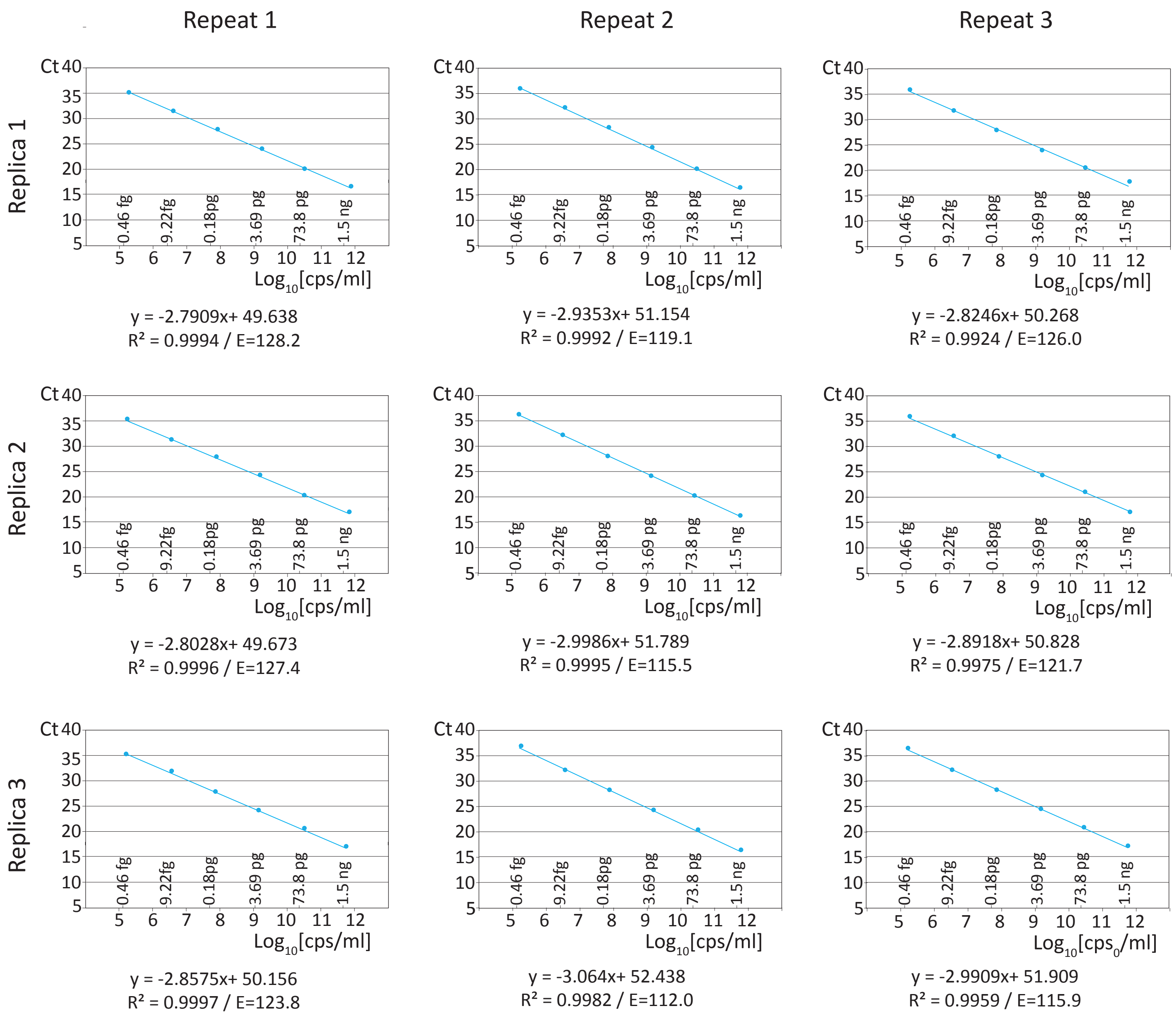

b- Standard curves for each repeat

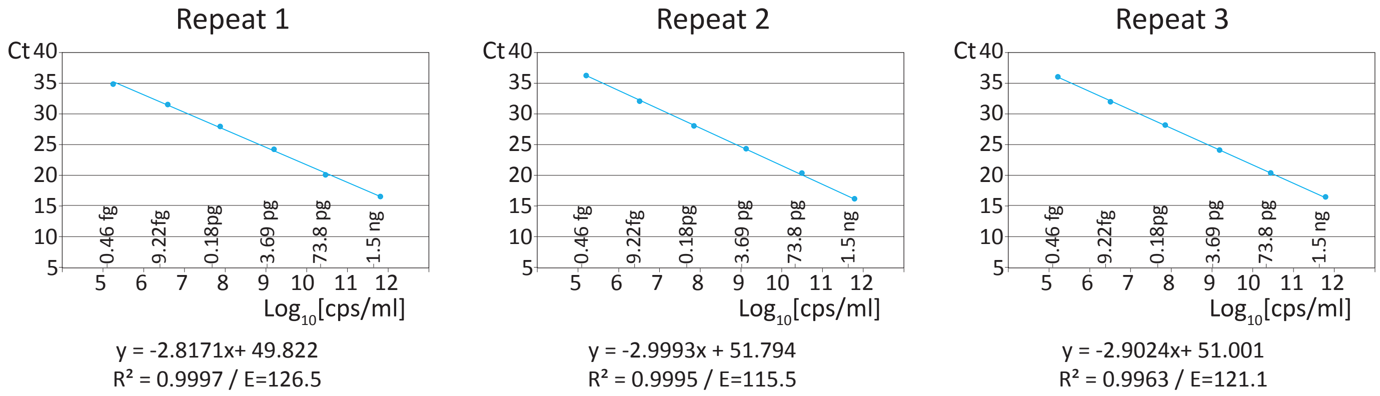

c- RNA copies (averaged from the 4 strains) general standard curve

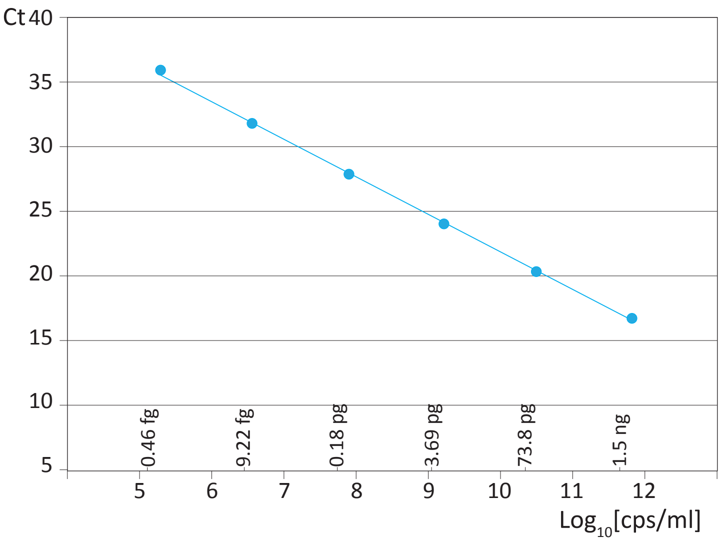

y = -2.9063x + 50.873
R^2 = 0.9993 / E=120.8

Figure S2.- Standard curves using RNA copies from crude virus: For each curve, the equation and its reliability (given as the coefficient of determination (R^2) and the efficiency (E) of the amplification) is shown. The graphs are organized in 5 sections: sections **A** to **D** with the curves obtained with each type strain, and calibration curves averaging the data from the 4 strains are shown in section **E**. In each section, the curves are shown in 3 ways: **a**-All the curves obtained with the corresponding viral type in every replica and repeat; **b**- the standard curve for each repeat (averaging the 3 replicas), and **c**- the general standard curve (averaging data from all replicas and repeats).

Supplementary Figure S3

A/ SJNNV type strain

a- Standard curves for each replica and repeat

Repeat 1

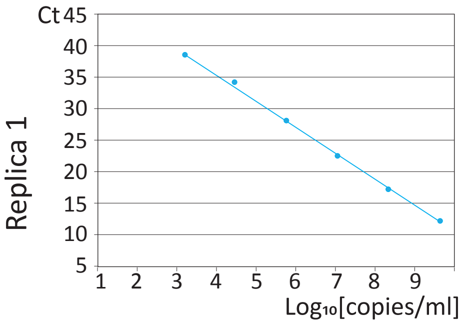

y = -4.1226x + 51.985
R^2 = 0.9986 / 84.1

Repeat 2

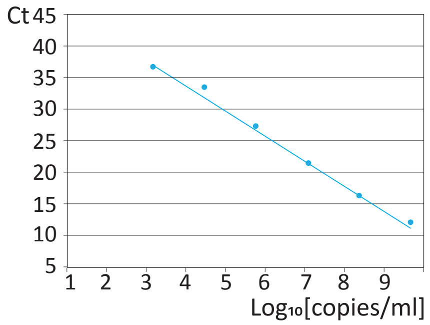

y = -3.9841x + 49.953
R^2 = 0.9950 / E=80.7

Repeat 3

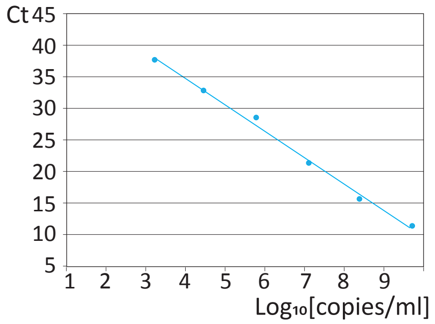

y = -4.2042x + 51.521
R^2 = 0.9953 / E=80.2

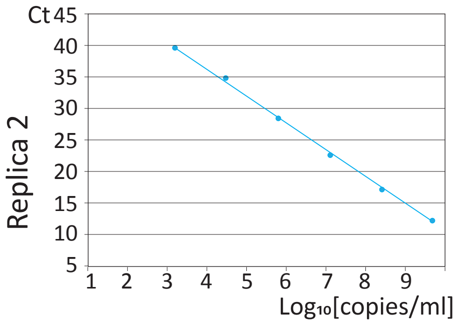

y = -4.2536x + 52.994
R^2 = 0.9973 / 82.9

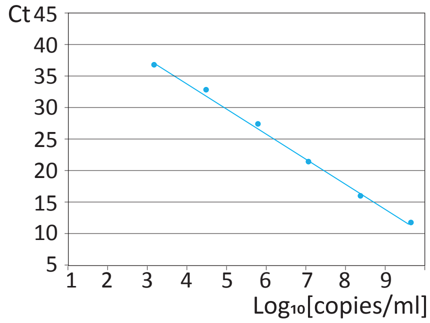

y = -3.997x + 49.842
R^2 = 0.9961 / E=87.0

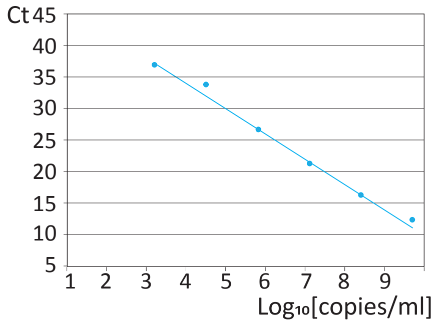

y = -3.9937x + 50.109
R^2 = 0.9925 / E=88.2

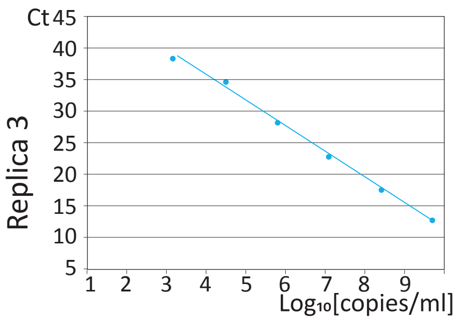

y = -4.1157x + 52.084
R^2 = 0.9955 / E=85.5

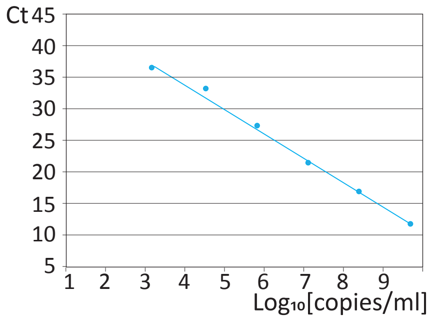

y = -3.9384x + 49.464
R^2 = 0.9954 / E=87.7

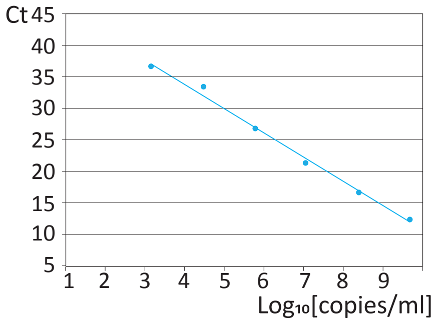

y = -3.9098x + 49.523
R^2 = 0.9944 / E=85.4

b- Standard curves for each repeat

Repeat 1

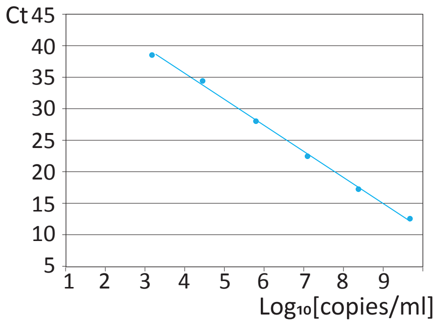

y = -4,164x + 52,354
R^2 = 0,9974 / E=84.2

Repeat 2

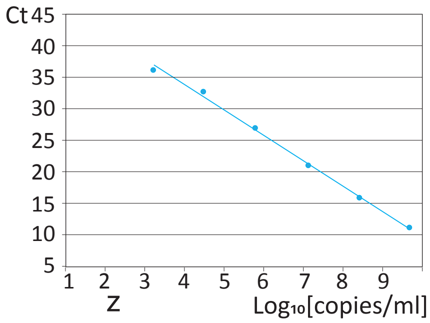

y = -3,9732x + 49,753
R^2 = 0,9957 / E=85.0

Repeat 3

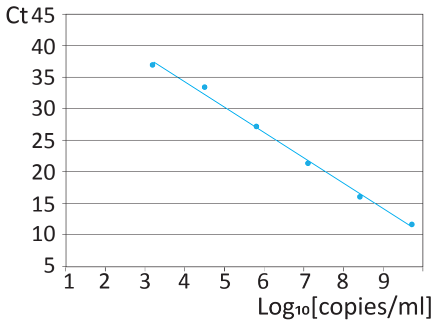

y = -4,0359x + 50,384
R^2 = 0,9958 / E=84.5

Supplementary Figure S3.- Standard curves using RNA copies from purified virus

c- RNA copies from purified SJNNV (averaged from all replicas and repeats): General standard curve

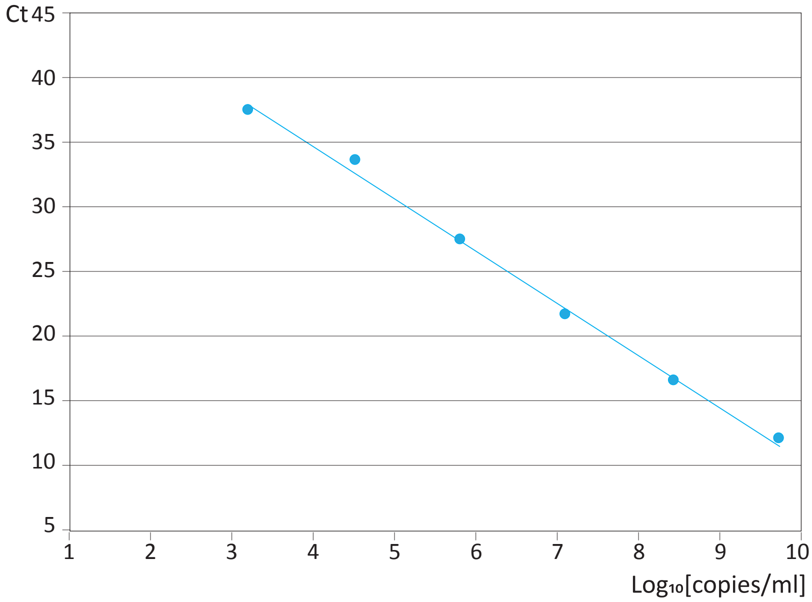

y = -4,0577x + 50,83
R^2 = 0,9965 / E=84.5

Supplementary Figure S3.- Cont.

B/ RGNNV type strain

a- Standard curves for each replica and repeat

Replica 1

Repeat 1

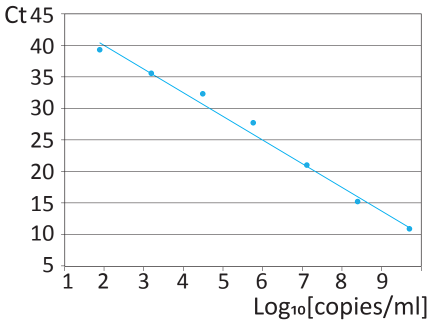

$y = -3.7717x + 47.643$
$R^2 = 0.9889$ / E=74.8

Repeat 2

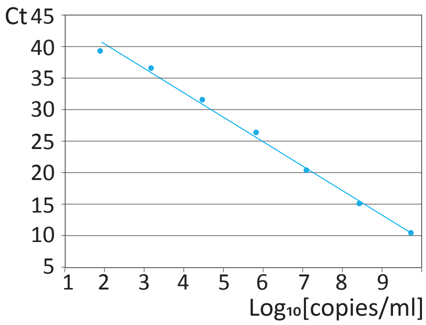

$y = -3.8935x + 48.071$
$R^2 = 0.9924$ / E=78.2

Repeat 3

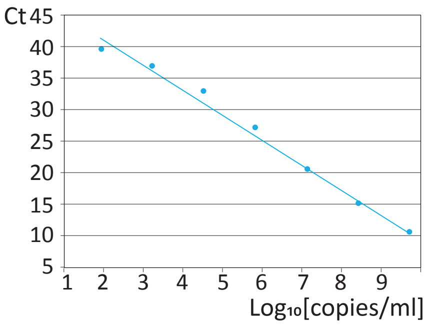

$y = -3.9089x + 48.655$
$R^2 = 0.9870$ / E=72.9

Replica 2

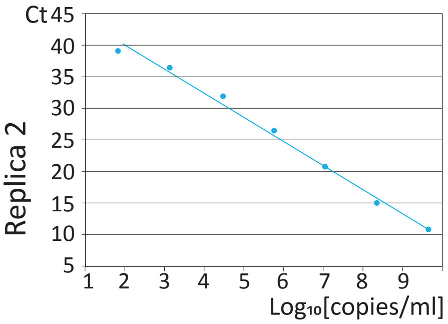

$y = -3.8137x + 47.866$
$R^2 = 0.9920$ / E=71.8

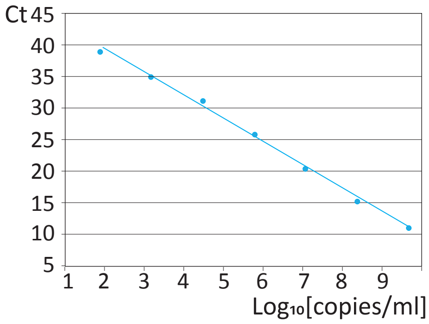

$y = -3.6794x + 46.569$
$R^2 = 0.9964$ / E=77.9

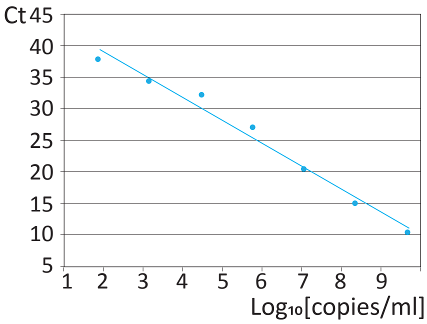

$y = -3.6422x + 46.603$
$R^2 = 0.9819$ / E=78.0

Replica 3

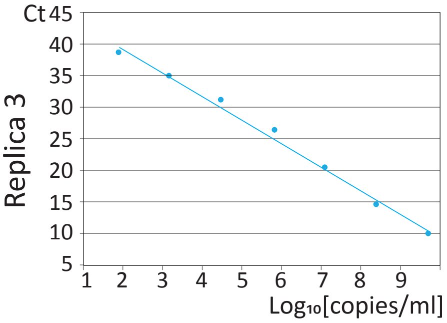

$y = -3.7268x + 46.87$
$R^2 = 0.9915$ / E=75.0

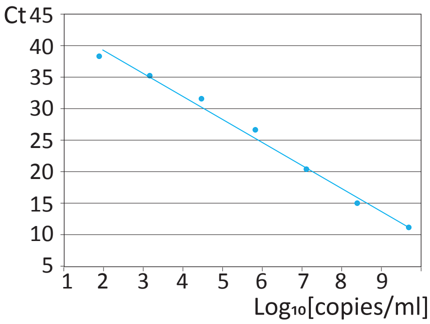

$y = -3.6564x + 46.437$
$R^2 = 0.9913$ / E=79.4

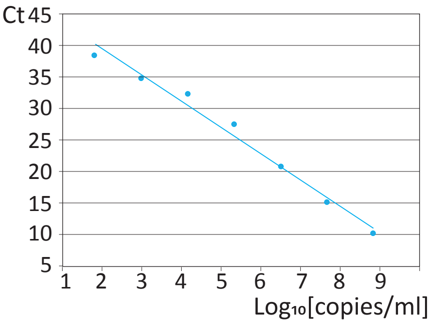

$y = -3.7297x + 47.248$
$R^2 = 0.9821$ / E=80.2

b- Standard curves for each repeat

Repeat 1

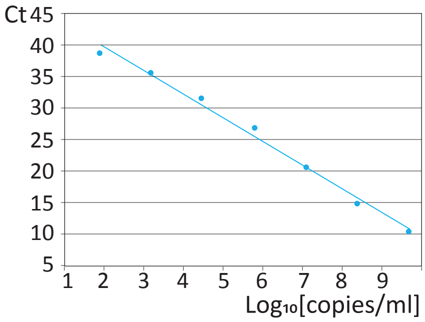

$y = -3,7707x + 47,46$
$R^2 = 0,9913$ / E=73.8

Repeat 2

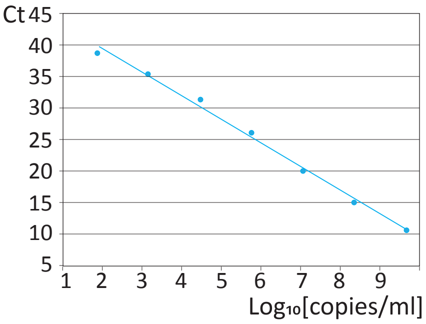

$y = -3,7431x + 47,026$
$R^2 = 0,9938$ / E=78.5

Repeat 3

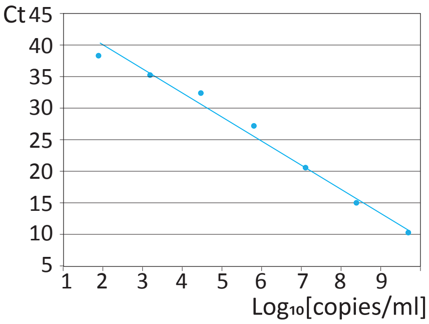

$y = -3,7603x + 47,502$
$R^2 = 0,9847$ / E=76.9

c- RNA copies from purified RGNNV (averaged from all replicas and repeats): General standard curve

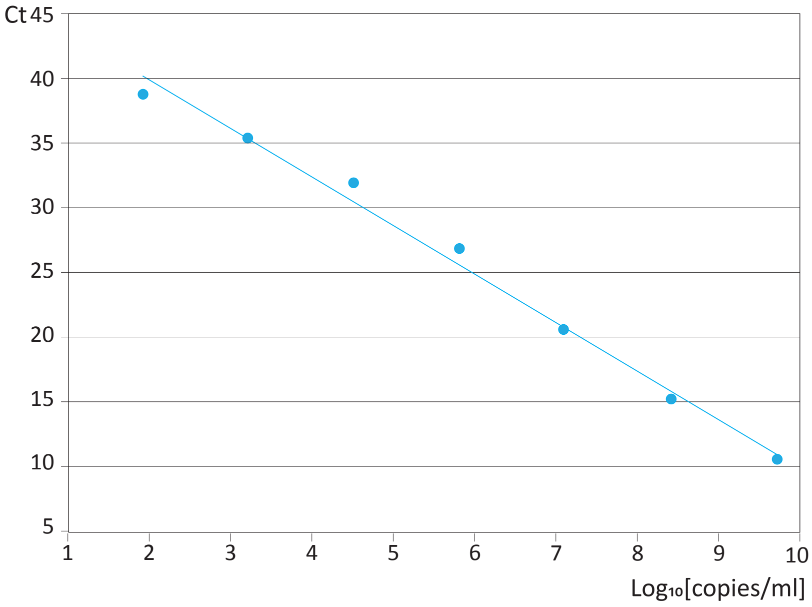

y = -3,758x + 47,329
R^2 = 0,9905 / E=76.4

Supplementary Figure S3.- Cont.

C/ Standard curves averaged from both strains

a- Standard curves for each replica and repeat

Repeat 1 | Repeat 2 | Rpeat 3

Replica 1

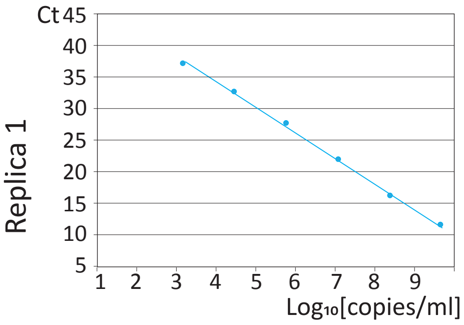

y = -4.0548x + 50.627
R^2 = 0.9965 / E=76.4

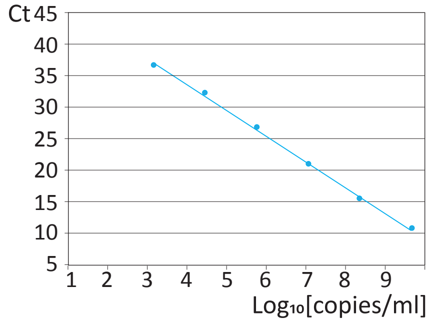

y = -4.0615x + 49.938
R^2 = 0.9974 / E=76.3

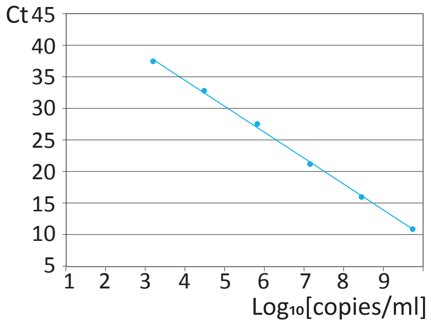

y = -4.2091x + 51.239
R^2 = 0.9961 / E=72.8

Replica 2

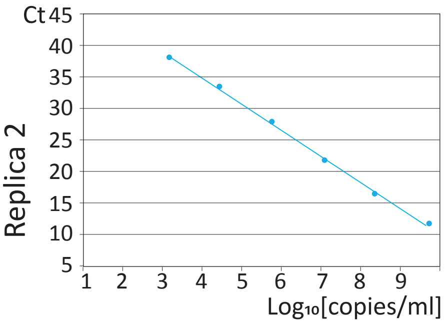

y = -4.1523x + 51.326
R^2 = 0.9977 / E=74.1

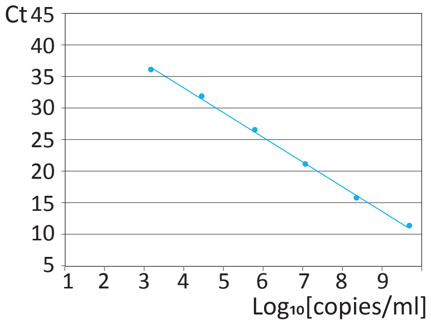

y = -3.8987x + 48.663
R^2 = 0.9968 / E=80.5

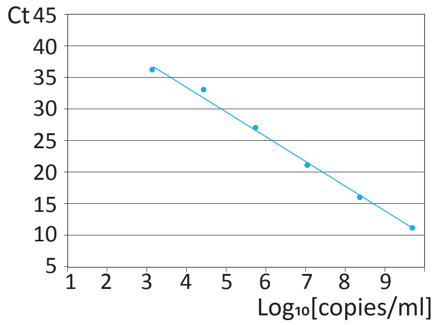

y = -3.9441x + 49.309
R^2 = 0.9917 / E=79.3

Replica 3

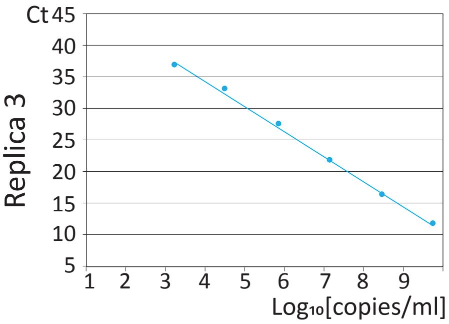

y = -4.0209x + 5.0229
R^2 = 0.9960 / E=77.3

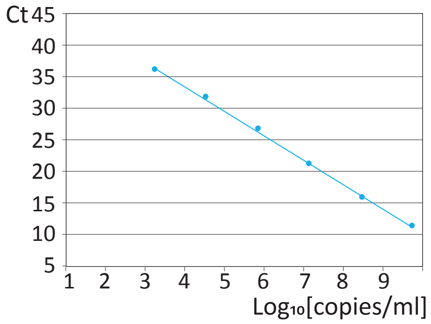

y = -3.9076x + 48.782
R^2 = 0.9968 / E=80.3

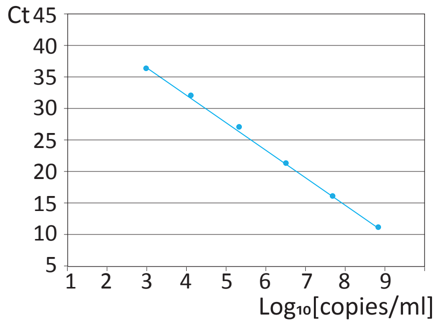

y = -3.9443x + 49.325
R^2 = 0.9927 / E=79.3

b- Standard curves for each repeat

Repeat 1 | Repeat 2 | Repeat 3

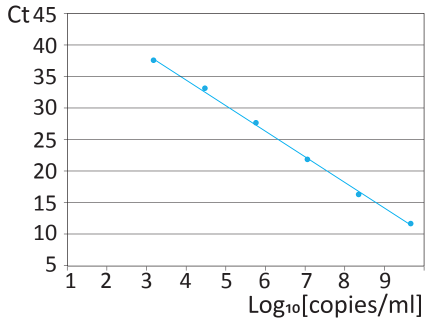

y = -4.0760x + 50.727
R^2 = 0.9970 / E=75.9

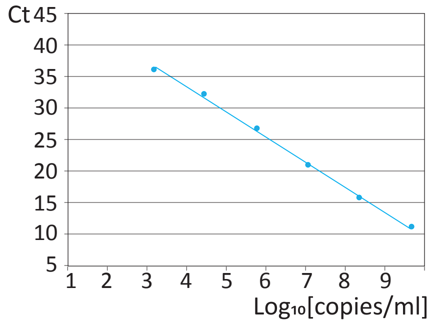

y = -3.9559x + 49.127
R^2 = 0.9969 / E=79.0

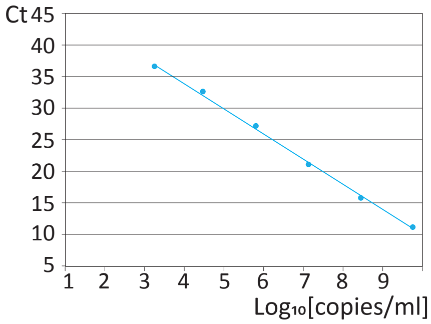

y = -4.0325x + 49.958
R^2 = 0.9942 / E=77.0

c- RNA copies (averaged from both strains) general standard curve

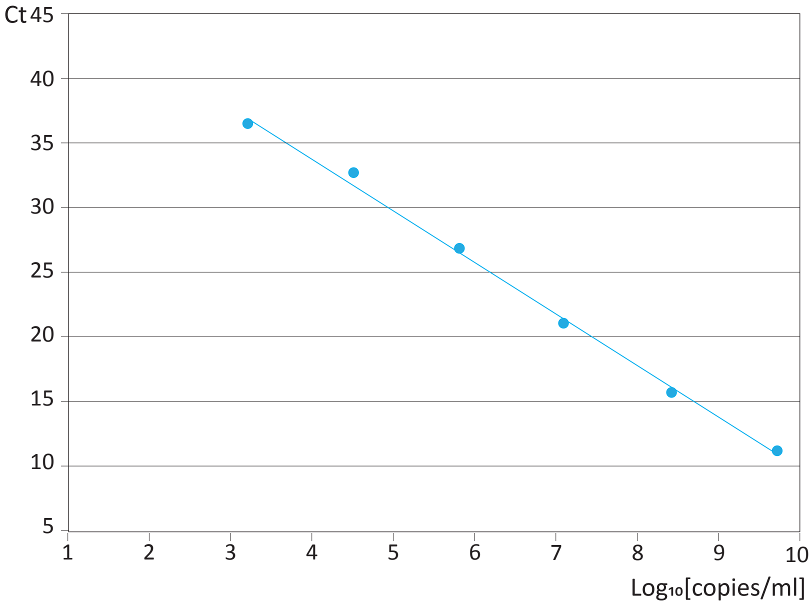

y = -4.0215x + 49.938
R^2 = 0.9962 / E=77.3

Supplementary Figure S3.- Standard curves using RNA copies from purified virus: For each curve, the equation and its reliability (given as the coefficient of determination (R^2) and the efficiency (E) of the amplification) is shown. The graphs are organized in 3 sections: sections **A** and **B** with the curves obtained with SJNNV and RGNNV type strains, and calibration curves averaging the data from both are shown in section **C**. In each section, the curves are show in 3 ways: **a**-All the curves obtained with both viral types in every replica and repeat; **b**- The standard curve for each repeat (averaging the 3 replicas), and **c**- the general standard curve (averaging data from all replicas and repeats).

Supplementary Figure S4

a- Standard curves for each replica and repeat

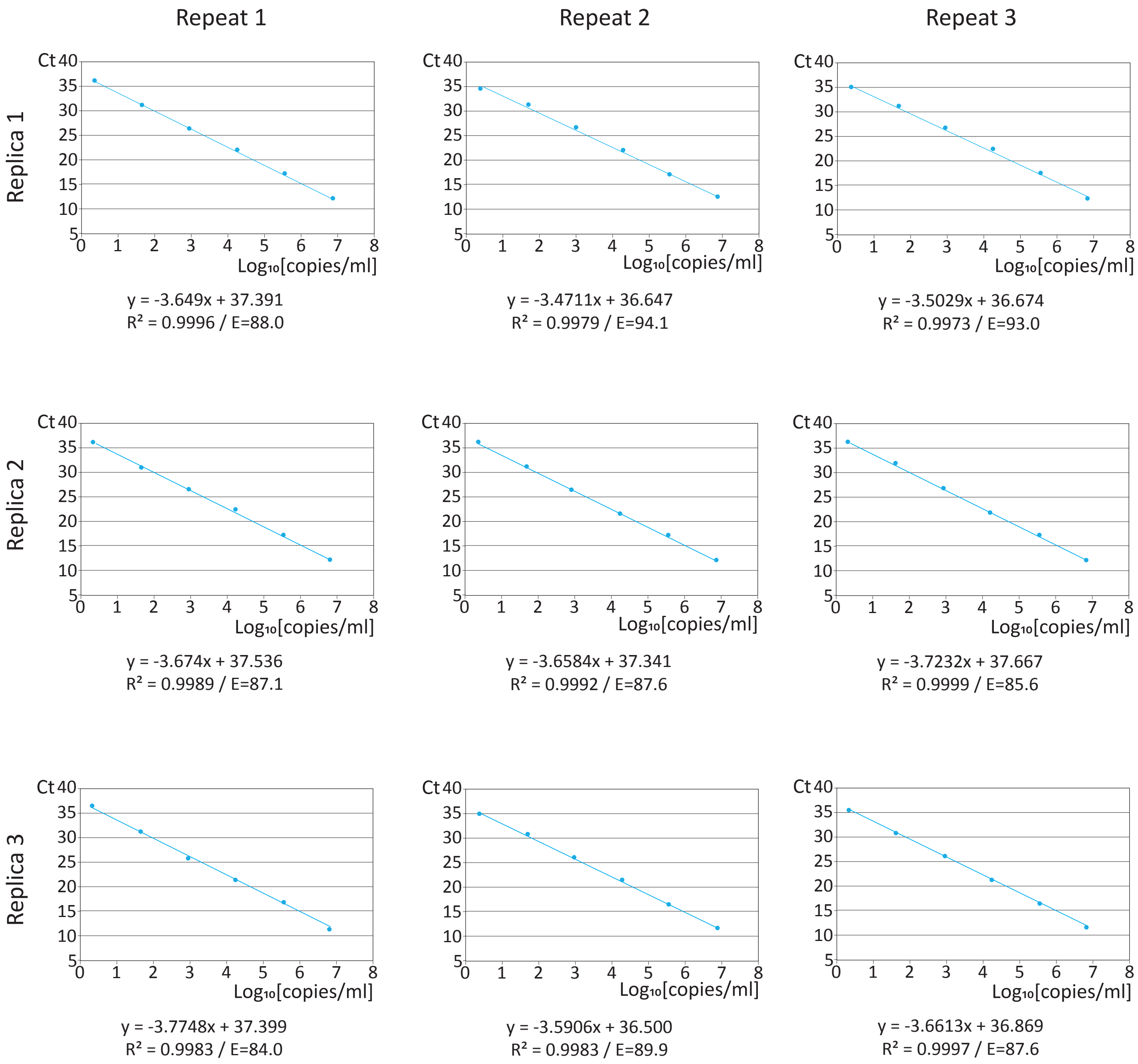

b- Standard curves for each repeat

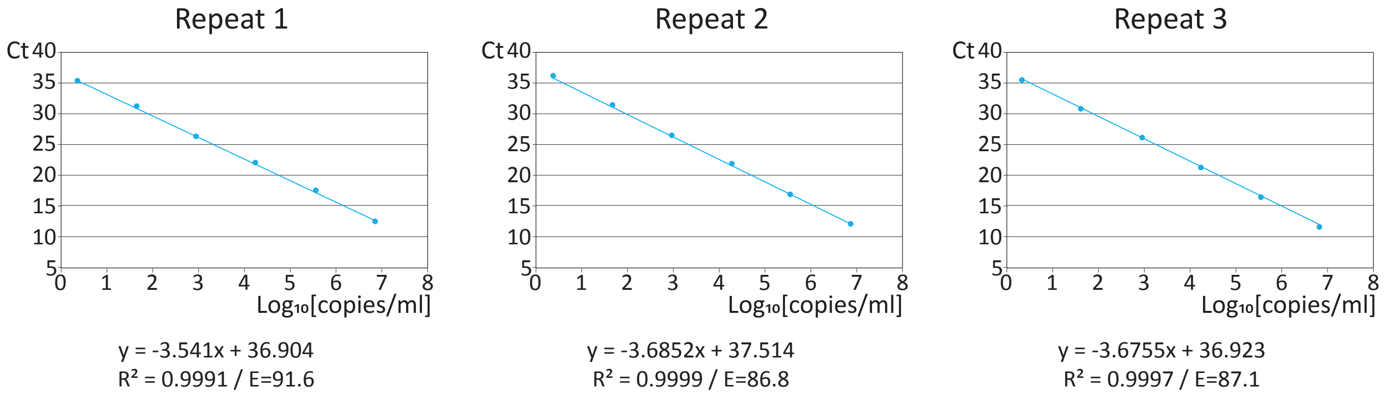

c- Plasmid copies (averaged from all replicas and repeats): General standard curve

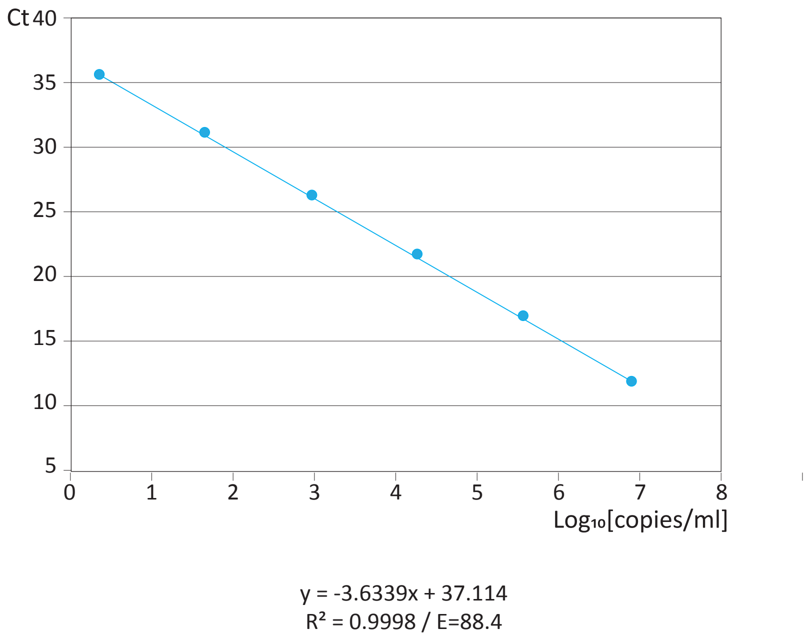

Supplementary Figure S4.- Standard curves with plasmid DNA corresponding to the RGNNV type: For each curve, the equation and its reliability (given as the coefficient of determination (R^2) and the efficiency (E) of the amplification) is shown. The curves are show in 3 ways: **a**-All the curves obtained in every replica and repeat; **b**- The standard curve for each repeat (averaging the 3 replicas), and **c**- the general standard curve (averaging data from all replicas and repeats).

Supplementary Figure S5

a- Standard curves for each replica and repeat

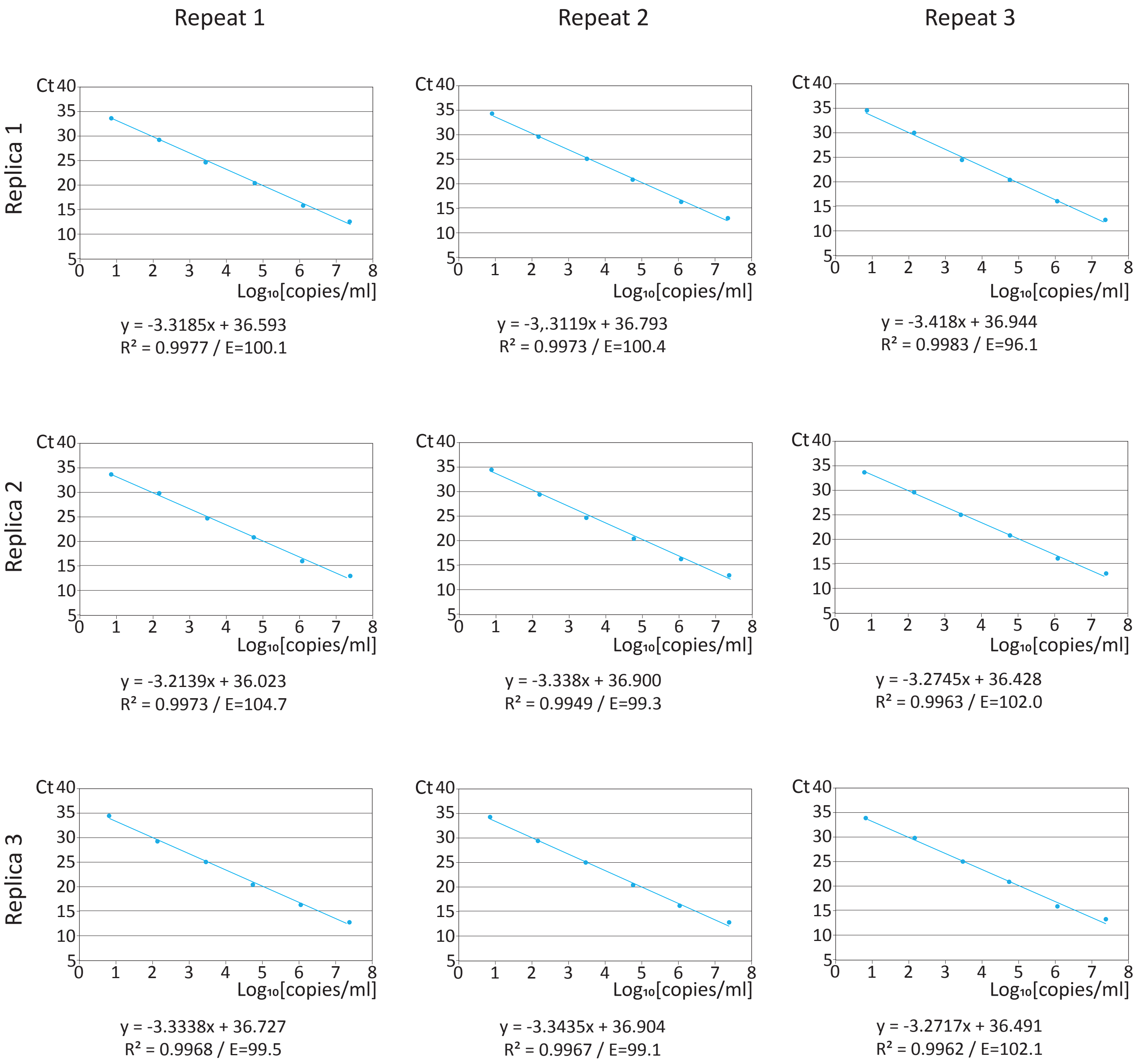

b- Standard curves for each repeat

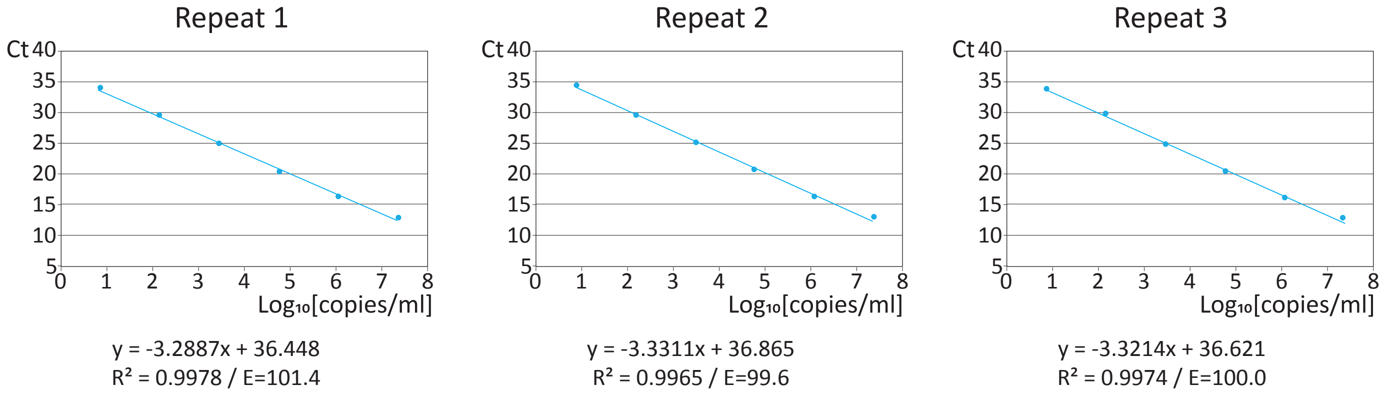

c- Plasmid copies (averaged from all replicas and repeats): General standard curve

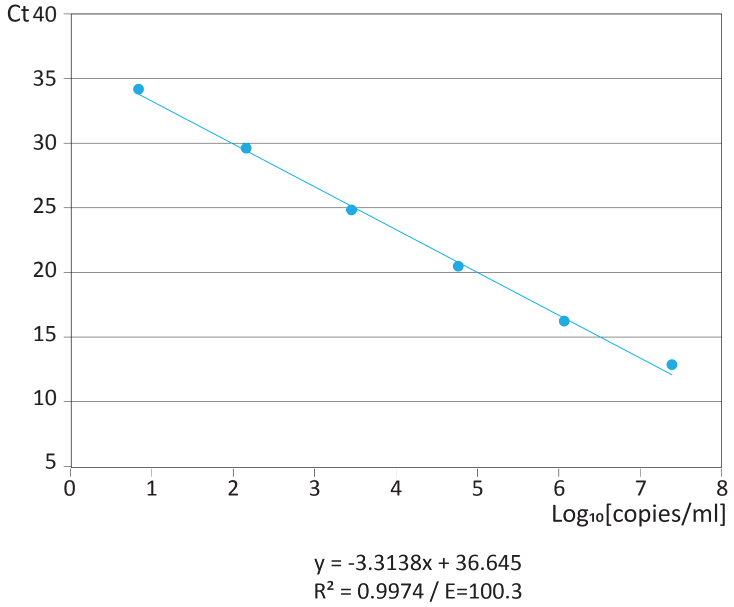

Supplementary Figure S5.- Standard curves with in vitro transcribed RNA corresponding to the RGNNV type: For each curve, the equation and its reliability (given as the coefficient of determination (R^2) and the efficiency (E) of the amplification) is shown. The curves are show in 3 ways: **a**-All the curves obtained in every replica and repeat; **b**- The standard curve for each repeat (averaging the 3 replicas), and **c**- the general standard curve (averaging data from all replicas and repeats).

Supplementary Figure S6

A/ RNA extraction A

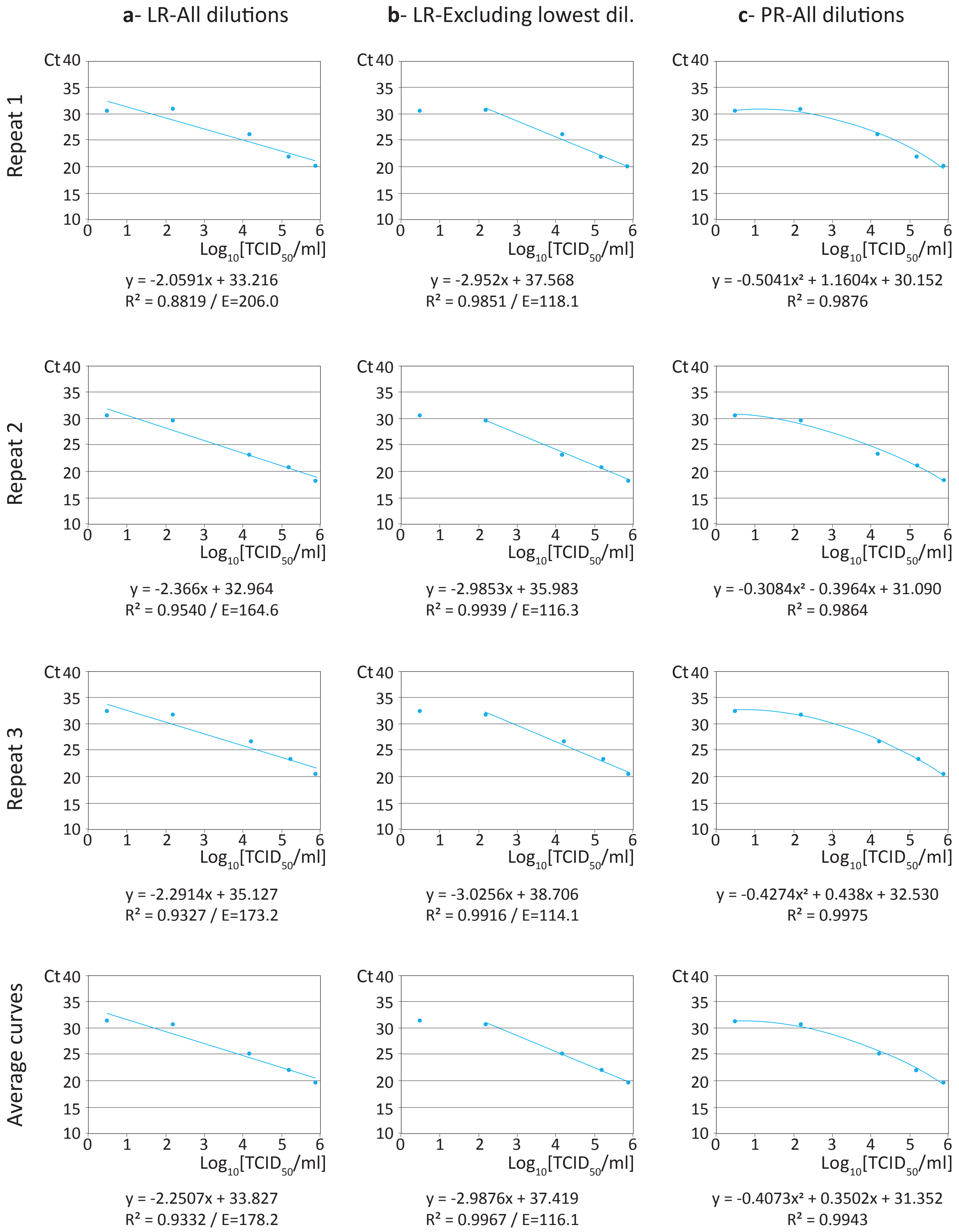

Supplementary Figure S6.- Cont.

B/ RNA extraction B

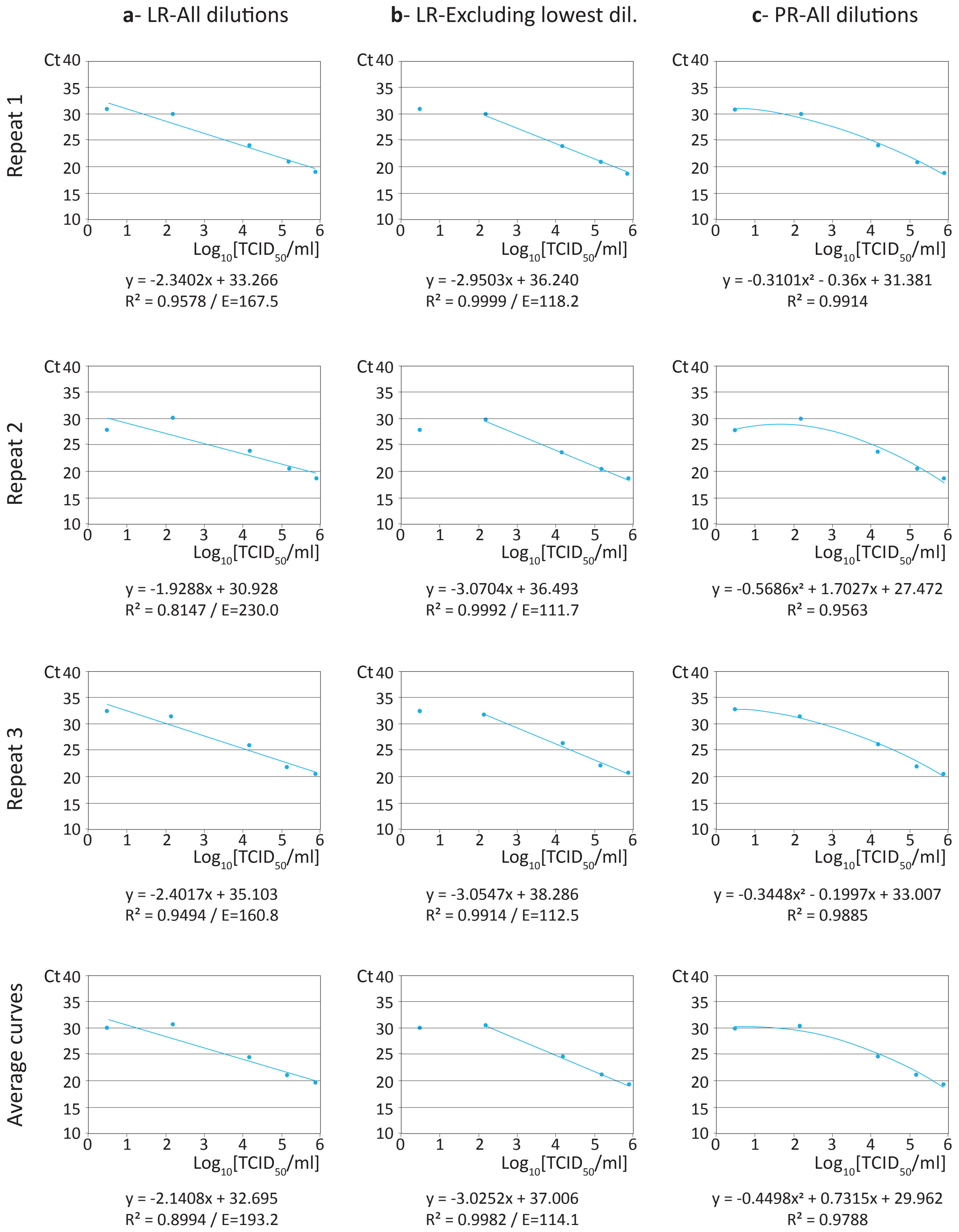

Supplementary Figure S6.- Cont.

C/ RNA extraction C

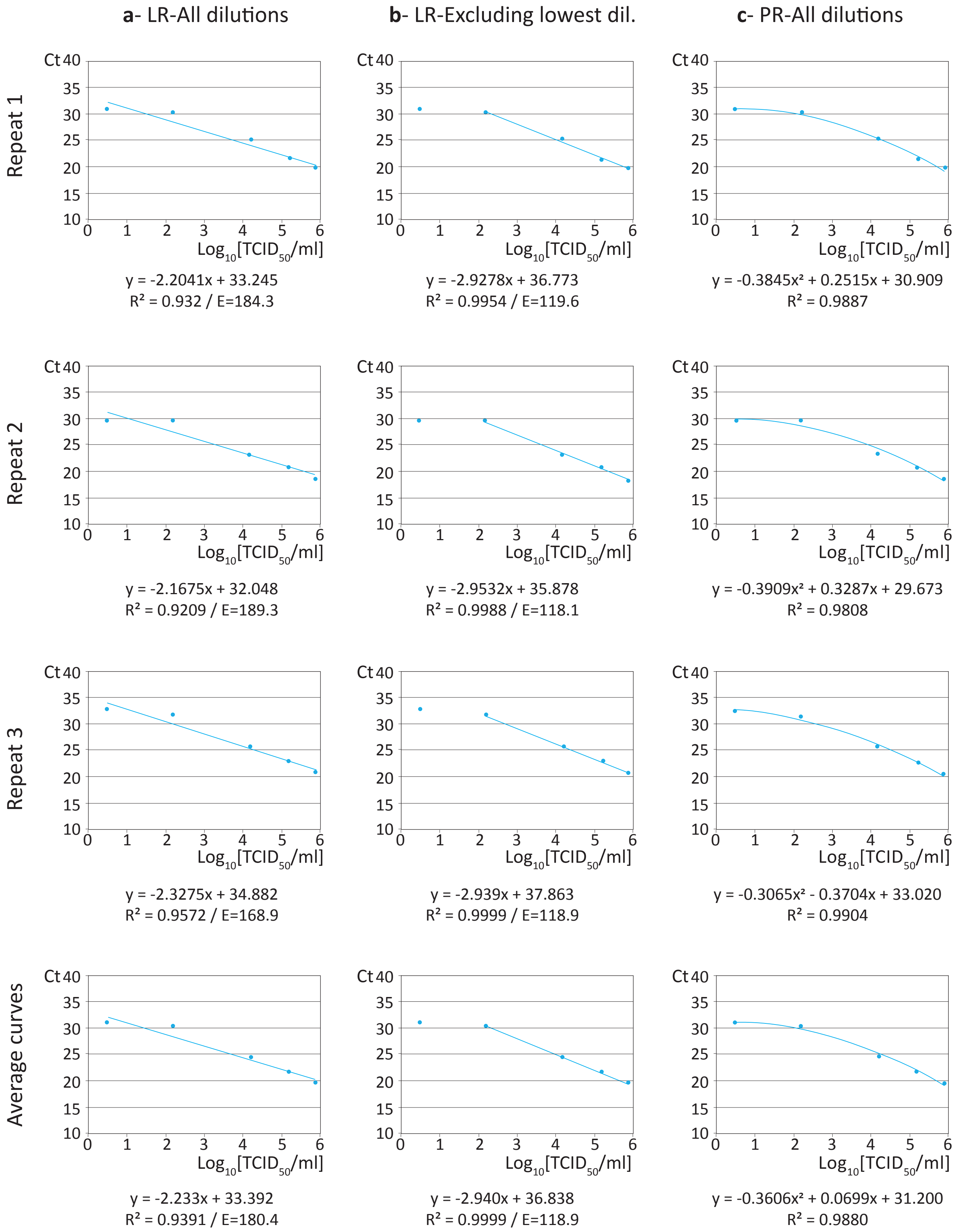

Supplementary Figure S6.- Cont.

D/ Averaged from the 3 RNA extractions

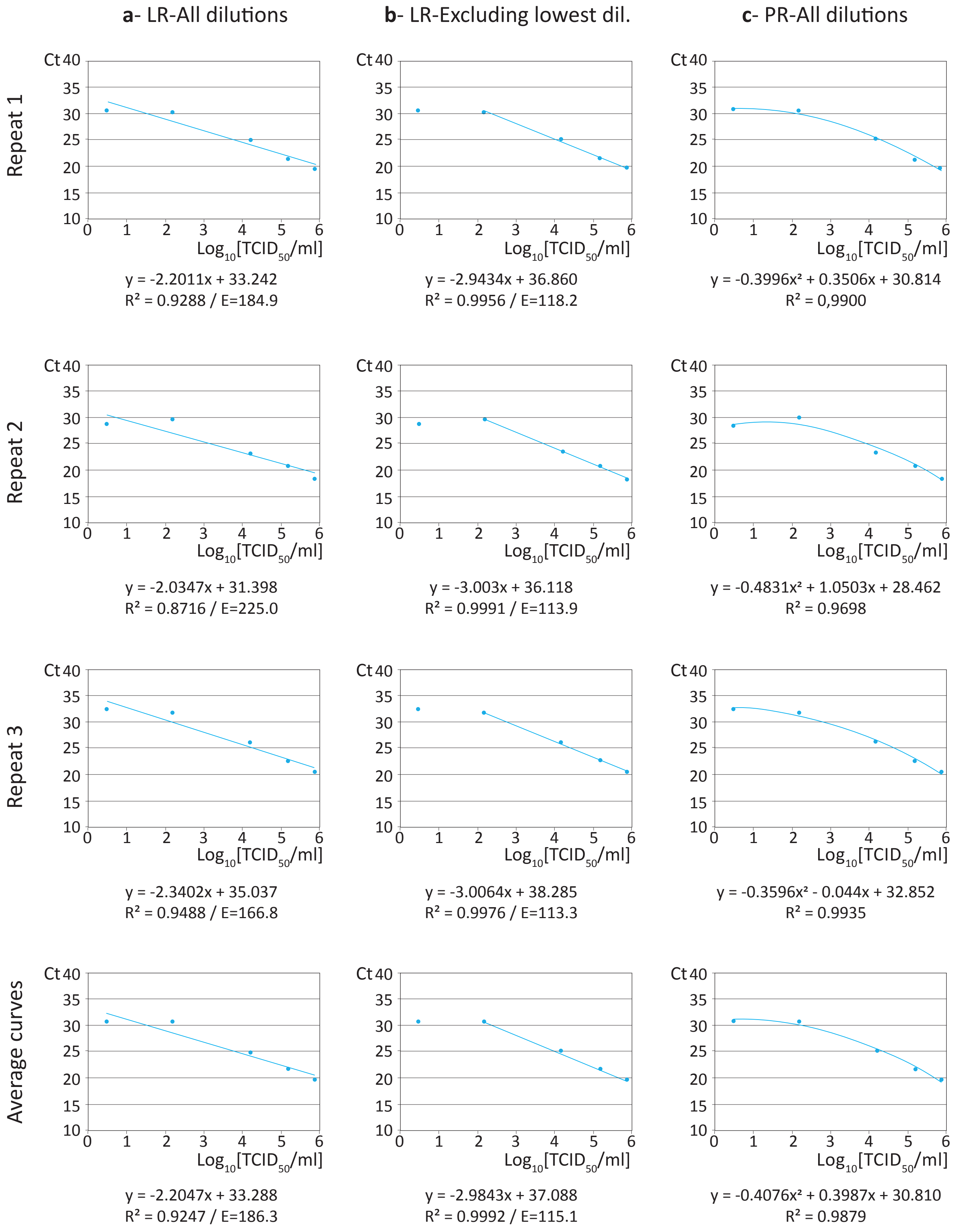

Supplementary Figure S6.- Detection and quantification of viral RNA from infected fish tissues: For each curve, the equation and its reliability (given as the coefficient of determination (R^2) and the efficiency (E) of the amplification) is shown. The results from the three RNA extractions performed are shown in sections A to C; Section D shows the average curves from the 3 extractions. For each extraction, the curves for each repeat (3 replicas per each) and the average curves are shown in 3 ways: a-Linear regression including all the dilutions; b-Linear regression excluding the lowest dilution, and c-a second degree polynomial regression, including all dilutions.
